# Supplementary material for: Cryptic mitochondrial DNA mutations coincide with mid-late life and are pathophysiologically informative in single cells across tissues and species
Source: Nat Commun. 2025 Mar 6;16:2250. doi: 10.1038/s41467-025-57286-8 (PMC11885543; doi:10.1038/s41467-025-57286-8)
Supplement: Supplementary file 1 — Supplementary Information [file 41467_2025_57286_MOESM1_ESM.pdf]

Supplementary Information: Cryptic mitochondrial ageing coincides with mid-late life and is pathophysiologically informative in single cells across tissues and species

Alistair P. Green*[1], Florian Klimm*[1,2], Aidan S. Marshall[1], Rein Leetmaa[1], Juvid Aryaman[1,2], Aurora Gómez-Durán[2,3], Patrick F. Chinnery[2], and Nick S. Jones†[1,4]

[1]Department of Mathematics & Centre for the Mathematics of Precision Healthcare, Imperial College London, South Kensington, London SW7 2AZ, United Kingdom
[2]Department of Clinical Neuroscience & Medical Research Council Mitochondrial Biology Unit, School of Clinical Medicine, University of Cambridge, Cambridge CB2 0QQ, UK
[3]MitoPhenomics Lab, Centro Singular de Investigación en Medicina Molecular y Enfermedades Crónicas (CiMUS), Universidade de Santiago de Compostela, Campus Vida Avenida Barcelona, s/n, 15782, A Coruña (Spain).
[4]I-X Centre for AI in Science, Imperial White City Campus, 84 Wood Lane, London W12 7SL, UK

February 5, 2025

Contents

S1 Supplementary Discussion: Truncated coalescent theory **S3**
S1.1 Moran model and coalescent theory . . . S3
S1.2 Application to extensions of the Moran model . . . S3
S1.3 The expected site frequency spectrum . . . S6
S1.3.1 Distribution of $S^W_{k,n}$ . . . S6
S1.3.2 Expectation of $T^W_{k,n}$ . . . S6
S1.3.3 Expected branch lengths . . . S7
S1.4 Distribution of number of mutations in a single cell . . . S8
S1.5 Multiple cells . . . S9
S1.6 Hierarchical Bayesian Inference . . . S9
S1.7 Comparison to the cryptic site frequency spectrum . . . S9

S2 Supplementary Discussion: Model inferences **S10**
S2.1 Comparison to simulation . . . S10
S2.2 Hierarchical simulation comparison . . . S10
S2.3 Model fitting to Data . . . S13
S2.4 Fitting to human data . . . S13
S2.5 Fitting to mouse data . . . S19
S2.6 Investigating sex-specific effects of mitochondrial ageing . . . S19
S2.7 Comparing diabetic to healthy tissue . . . S19

S3 Supplementary Discussion: Fisher's method for DEG identification **S22**
S3.1 Fisher's method for donors . . . S22
S3.2 Fisher's method for cell types . . . S22

S4 Supplementary Discussion: The role of the number of mitochondrial reads **S25**

S5 Parkinson's disease and Alzheimer's disease single-nucleus RNA-seq data **S25**

*Both authors contributed equally.
†Corresponding author: nick.jones@imperial.ac.uk

S6 Supplementary Discussion: Variant Calling from RNA **S27**
S6.1 Empirical corroboration . . . S27
S6.2 Robustness to variant calling errors . . . S27
S6.3 The effect of UMI collapsing on heteroplasmy calls . . . S28
S6.4 Mutations detected in snATAC data recapitulate results found from scRNA derived mutations . . . S29

S7 Supplementary Tables **S31**

S8 Supplementary Figures **S32**
S8.1 Differing sequencing techniques have varying coverage of the mitochondrial genome resulting in fewer possible variant locations . . . S32
S8.2 Increasing age difference evolves the cSFS further apart . . . S37
S8.3 Restricting to a single cell type leaves results unchanged . . . S38
S8.4 Mitochondrial ageing has multiple eras corresponding to mutations accumulating at different heteroplasmy levels S39
S8.5 Correlation between donor age and mitochondrial load as a function of the heteroplasmy thresholds . . . S40
S8.6 Mammalian lung single-cell RNA data . . . S42
S8.7 Volcano plots for full-length human pancreas data at different heteroplasmy thresholds h . . . S43
S8.8 Volcano plots for all data sets . . . S44
S8.9 Processing pipeline . . . S51
S8.10 Differentially expressed genes for location-specific cryptic mtDNA mutations in human pancreas . . . S53

S1 Supplementary Discussion: Truncated coalescent theory

S1.1 Moran model and coalescent theory

In this supplementary discussion, we construct a mathematical model that allows us to describe and predict the accumulation of somatic mtDNA mutations throughout ageing. We seek an analytic formula describing the expected time-evolution of the expected number of mutations at every heteroplasmy (the site frequency spectrum, SFS). We extend the typical notion of the SFS to include mutations at 100 % heteroplasmy (homoplasmy) using tools from population genetics (see (*1*) for an introduction).

A cell's mtDNA copy number is regulated by mitophagy and replication in order to satisfy cellular energy requirements (*2*). A simple forwards-in-time model for the mtDNA population of a single post-mitotic cell is a birth–death model with mutation assuming a fixed population size (*3*). Crucially, in the following, we consider the scenario that, at some start time, W, no mutations (unique to that cell) are present in the system: a model for post-mitotic *de novo* mutation. This means that mutations gradually accumulate in the cell and the SFS, is out of equilibrium, and evolves from its form at start-time W to the present. This means that mutations gradually accumulate in the cell and the SFS, is out of equilibrium, and evolves from its form at start-time W to the present. In the Moran model, birth–death events, hereafter Moran events, are linked so that at all times the population size N remains fixed, and every time an event occurs one copy of the mtDNA is randomly chosen for replication and another is randomly chosen for death (Fig. S1a). As replication can occur on a much shorter timescale, compared to the half-life of mtDNA, we let the exponential rate of Moran events be $\Lambda = \frac{N\ln(2)}{t_{1/2}}$, where $t_{1/2}$ is the half life of mtDNA. During replication of an mtDNA, we assign a probability of mutation $P(\text{mut}) = u$ which can be linked to the error rate ν per replication per base of POLG by assuming a mutation can occur during replication on any base with equal probability. Under this assumption the number m of mutations during replication of mtDNA with B_{mtDNA} bases is binomially distributed, $m \sim \text{Binomial}\big(B_{\text{mtDNA}}, \nu\big)$, and so yields a probability of an mtDNA being mutated after replication $u = 1 - P(m = 0)$. For a sufficiently small base mutation rate ν, $u = \nu B_{\text{mtDNA}}$.

By using the Moran model as our forward-in-time model for mtDNA in a single cell, we can formulate a backwards-in-time equivalent process based on the *Kingman coalescent* (*4*) to derive the properties of an out of equilibrium SFS. The Kingman coalescent focuses on deriving the tree structure which relates a sample of n individuals from a population N. Consider a single Moran event, one member of the population of n individuals is chosen to replicate producing two offspring, and another to die (this can be the same member). The only way for a pair of lineages to coalesce is if they both belong to the two offspring of the Moran event, and if we randomly select two members of the population just after this event, the probability that both are offspring is $2/N^2$ giving us the coalescence probability. Extending this to a sample of k individuals, the probability that any two of them are offspring of the event is $k(k-1)/N^2$. Using the time distribution between Moran events, multiplied by the probability that a Moran event resulted in a coalescence tell us that the time it takes k lineages to coalesce to $k-1$ lineages is exponentially distributed with a rate of $\Lambda k(k-1)/N^2$ where Λ is the rate Moran events. It is typical in coalescent theory to re-scale time such that the coalescence rate of k lineages is $\binom{k}{2}$, and we work in this re-scaled time when working with the coalescent throughout this derivation.

We wish to produce a theory describing the evolution of the SFS over time supposing that cells initially have no mutations present. We do this by adapting the standard Kingman coalescent with mutation in the following way:

- The coalescent process runs as standard for times $t < W$, and
- At time $t = W$ all remaining coalescence occurs.

This modification is the same as assuming that for all times $t \geq W$, there is no turnover in a population of identical DNA sequences, only for a Moran process with mutation to switch on at $t = W$ and begin introducing mutations into the population. It is also equivalent analytically to the case where there are Moran dynamics, but zero mutation rate happening at $t \geq W$ (Fig. S1a-e). . We call this modified coalescent the *W-Truncated coalescent*, and any quantities with a superscript W refer to this process, while quantities without a superscript are results from standard coalescent theory. To convert any of the following results into proper time τ, we substitute $W = \frac{2\ln 2}{N t_{1/2}}\tau$.

S1.2 Application to extensions of the Moran model

Though we have formulated our forward-in-time model in the simplest case that the copy number of mtDNA stays constant and no mutation is allowed to recur, the coalescent also gives a good approximation in the case that copy number is allowed to fluctuate, so long as those fluctuations are fast compared to the coalescent timescale (*5*). In this case the coalescent dynamics are obeyed if time is suitably rescaled using an effective population size. Assuming that the population is allowed to vary according to a discrete time Markov chain, the effective population size will be equal to the average population size, calculated by the harmonic mean of possible population sizes with the averaging done over the stationary distribution (*6*, *7*). This use of an effective population size is common in statistical genetics, and there could be many reasons why the observed number of mtDNA in a cell will not necessarily correspond to the effective population size, for instance (*8*) fusion in the mitochondrial network can modulate dynamics. When mutations are allowed to occur on a genome of finite size (including the possibility

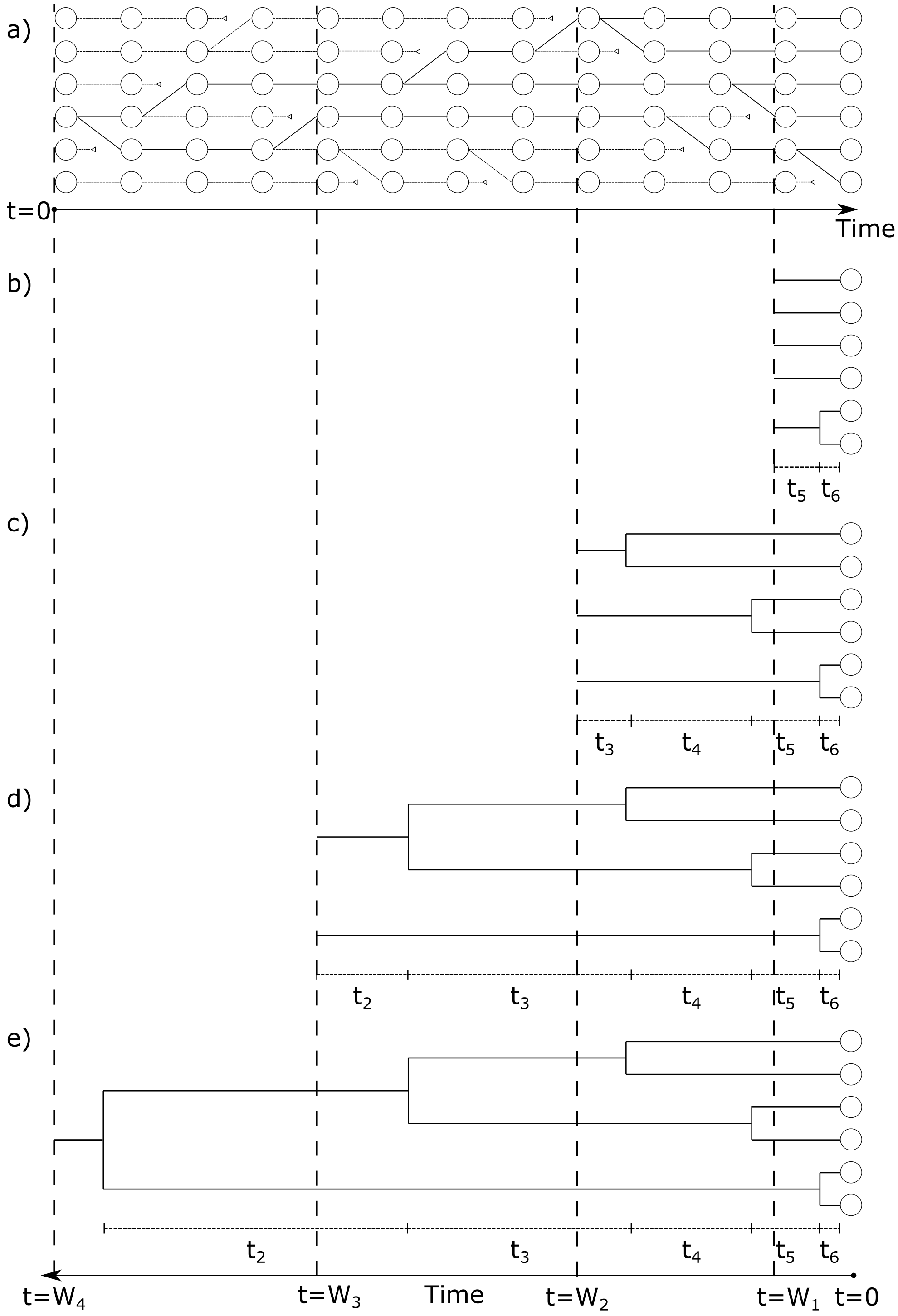

Supplementary Figure S1: **The dynamics of the W-truncated coalescent.** (a) A Moran process where at every time step one member of the population is chosen to replicate and another chosen to die. (b-e) A Moran process can run for an arbitrary period of time, but for the W-truncated coalescent we are only interested in the dynamics backwards-in-time up to a time W. When working backwards in time we call the length of time the Moran process was allowed to run for the 'truncation time', as it is at this point we stop all remaining dynamics of the coalescent process. By increasing the truncation time W from W_1 to W_4, we look progressively further backwards in time along the Moran process shown in (a). The resulting coalescent trees drawn from samples at different time points are clearly different, with earlier samples resulting in shorter coalescent trees with coalescent times t_k dependent on the truncation time W. At the very short Ws shown in (b-d), not enough time has passed for all coalescent events to have occurred, and we see that for shorter Ws there are far fewer coalescent times. Increasing W further in (e) has allowed all coalescent events to have occurred, and only after this has happened can homoplasmic mutations begin to be seen in the sampled population.

of a mutation returning a mutant to the wildtype state), provided that the number of mutations in the population remains low compared to the size of the genome the distribution of the site frequency spectrum will be very similar to the infinite sites case. To demonstrate this, we performed simulations using both the standard infinite sites Moran model, and a model where birth and death are not linked and mutations occur on a genome of finite size. In these simulations the birth rate, λ, is set to fluctuate around the death rate, μ, in order to control the copy number, N, to a steady state value, N_{ss}. The equation for this is given as $\lambda = \mu + c(N - N_{ss})$. The parameter values for these simulations were: $\mu = 0.046$ days (*9*, *10*), $\nu = 4 \times 10^{-8}$ per base per replication (*11*), and $N_{ss} = 1000$ (*12*). We tested simulations across three orders of magnitude for the control parameter $c = 0.01, 0.001, 0.0001$ and found consistent results across all simulations. In figure S2 we show a comparison between the site frequency spectra of a Moran model simulation compared with that of the more complicated model with control $c = 0.0001$ to allow the largest fluctuations in population size. 3000 cells were simulated for 100 years under each model, and we find no significant difference in the site frequency spectra at any time points.

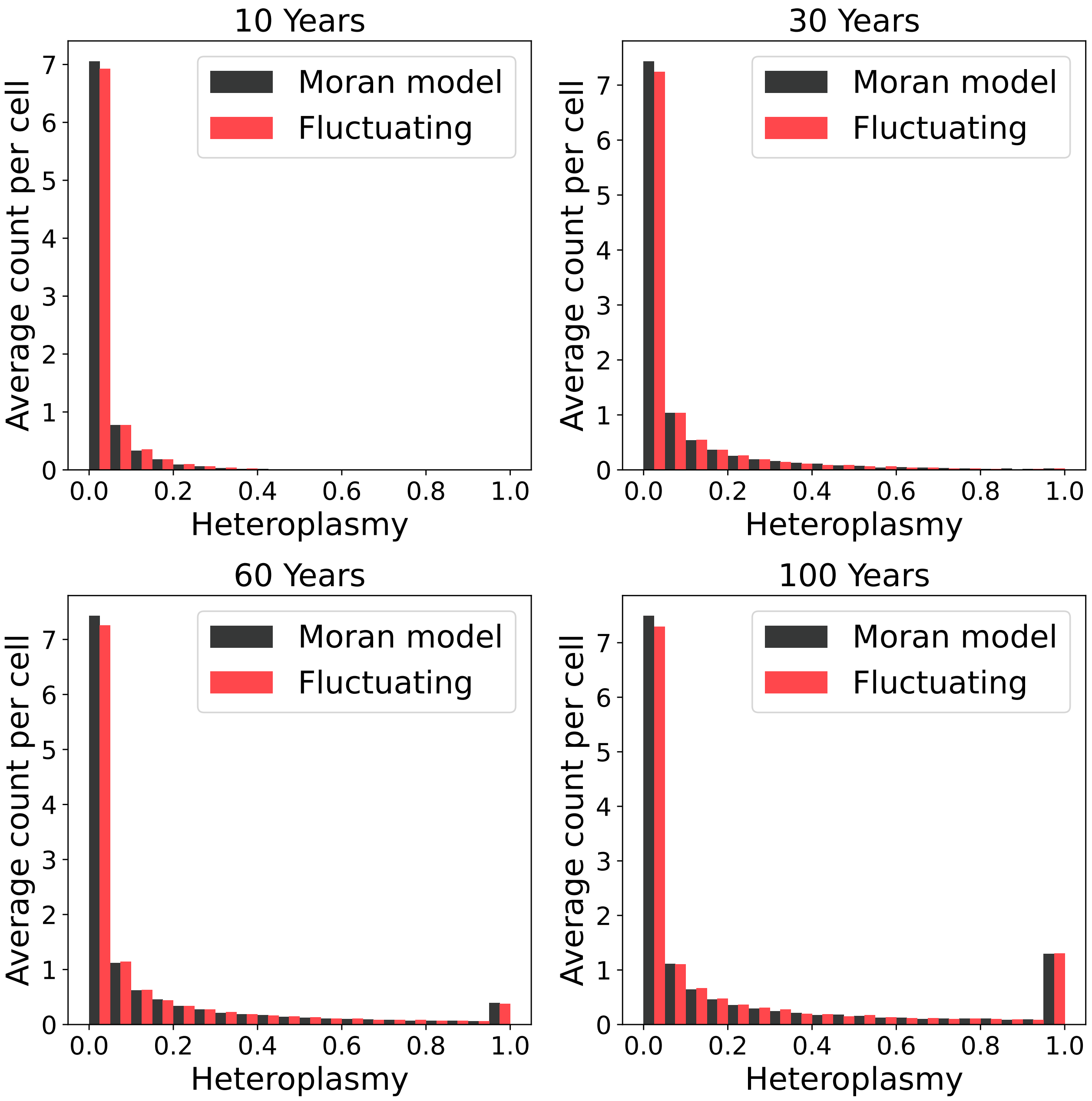

Supplementary Figure S2: **The infinite sites Moran model accurately matches more complicated population and mutation dynamics.** With a model allowing birth and death events independently of each other, with rates controlled to a steady-state population size, and mutations to occur on a genome of finite length (16569 bases), we find no significant difference with the site frequency spectra produced by the infinite sites Moran model.

S1.3 The expected site frequency spectrum

To find the form of the SFS we need to first find the distribution of coalescence times in the *W-Truncated coalescent*. We do this through finding $S^W_{k,n}$, the random variable giving the time at which k lineages coalesce in the *W-Truncated coalescent* process with a sample size of n. From this we can find the coalescence times using the expectation of the difference between time k and $k+1$ lineages coalesce: $\mathbb{E}(T^W_{k,n}) = \mathbb{E}(S^W_{k,n}) - \mathbb{E}(S^W_{k+1,n})$.

S1.3.1 Distribution of $S^W_{k,n}$

$S^W_{k,n}$ is distributed as in the conventional coalescent (the random variable $S_{k,n}$) for $t < W$ but for any $t \geq W$ all coalescence happens at W.

$$S^W_{k,n} = \begin{cases} S_{k,n} & 0 \leq t < W \\ W & t \geq W \end{cases} \tag{1}$$

$$P(S^W_{k,n} = t) = \begin{cases} P(S_{k,n} = t) & 0 \leq t < W \\ P(S_{k,n} \geq W) & t = W \end{cases} \tag{2}$$

$S_{k,n}$ is hypo-exponentially distributed so:

$$P(S_{k,n} = t) = \sum_{i=k}^{n} \binom{i}{2} Z^{\mathrm{n}}_{\mathrm{k,i}} e^{-\binom{i}{2}t}$$

where

$$Z^{\mathrm{n}}_{\mathrm{k,i}} := \prod_{\substack{j=k \\ j \neq i}}^{n} \frac{\binom{j}{2}}{\binom{j}{2} - \binom{i}{2}} \tag{3}$$

$Z^{\mathrm{n}}_{\mathrm{k,i}}$ has the identities:

$$\sum_{i=k}^{n} Z^{\mathrm{n}}_{\mathrm{k,i}} = 1, \tag{4}$$

$$\sum_{i=k}^{n} Z^{\mathrm{n}}_{\mathrm{k,i}} \binom{i}{2}^{-1} = \sum_{i=k}^{n} \binom{i}{2}^{-1}, \tag{5}$$

$$\sum_{i=k}^{n} Z^{\mathrm{n}}_{\mathrm{k,i}} \binom{i}{2}^{a} = \begin{cases} 0 & 1 \leq a < n-k+1 \\ (-1)^{n-k} \prod_{j=k}^{n} \binom{j}{2} & a = n-k+1 \end{cases} \tag{6}$$

Continuing the derivation, we look for $P(S_{k,n} \geq W)$:

$$\begin{aligned} P(S_{k,n} \geq W) &= \int_W^\infty P(S_{k,n} = t)dt, \\ &= \sum_{i=k}^{n} Z^{\mathrm{n}}_{\mathrm{k,i}} e^{-\binom{i}{2}W} \end{aligned}$$

This gives us the distribution of times to k^{th} coalescence as:

$$P(S^W_{k,n} = t) = \begin{cases} \sum_{i=k}^{n} \binom{i}{2} Z^{\mathrm{n}}_{\mathrm{k,i}} e^{-\binom{i}{2}t} & 0 \leq t < W \\ \sum_{i=k}^{n} Z^{\mathrm{n}}_{\mathrm{k,i}} e^{-\binom{i}{2}W} & t = W \end{cases} \tag{7}$$

S1.3.2 Expectation of $T^W_{k,n}$

The difference between the time k and $k+1$ lineages coalesce is defined as $T^W_{k,n} \triangleq S^W_{k,n} - S^W_{k+1,n}$.

$$\mathbb{E}(T^W_{k,n}) = \mathbb{E}(S^W_{k,n}) - \mathbb{E}(S^W_{k+1,n})$$

We first compute the expectation of the distribution of times to k^{th} coalescence as

$$
\begin{aligned}
\mathbb{E}(S_{k,n}^W) &= \int_0^W tP(S_{k,n}=t)dt + WP(S_{k,n}^W = W), \\
&= \sum_{i=k}^{n} Z_{\mathrm{k,i}}^{\mathrm{n}} \left(\int_0^W \binom{i}{2} t e^{-\binom{i}{2}t} dt + W e^{-\binom{i}{2}W} \right), \\
&= \sum_{i=k}^{n} Z_{\mathrm{k,i}}^{\mathrm{n}} \left(\binom{i}{2}^{-1} (1 - \binom{i}{2} W e^{-\binom{i}{2}W} - e^{\binom{i}{2}W}) + W e^{-\binom{i}{2}W} \right), \\
&= \sum_{i=k}^{n} Z_{\mathrm{k,i}}^{\mathrm{n}} \binom{i}{2}^{-1} (1 - e^{-\binom{i}{2}W}).
\end{aligned} \tag{8}
$$

Eq. 8 can be simplified further using Eq. 5 leaving:

$$
\begin{aligned}
\mathbb{E}(S_{k,n}^W) &= \sum_{i=k}^{n} \binom{i}{2}^{-1} (1 - Z_{\mathrm{k,i}}^{\mathrm{n}} e^{-\binom{i}{2}W}), \\
&= \mathbb{E}(S_{k,n}) - \sum_{i=k}^{n} \binom{i}{2}^{-1} Z_{\mathrm{k,i}}^{\mathrm{n}} e^{-\binom{i}{2}W}.
\end{aligned} \tag{9}
$$

From Eq. 9 follows the anticipated behaviour in the limits of W

$$
\begin{aligned}
\lim_{W\to\infty} \mathbb{E}(S_{k,n}^W) &= \mathbb{E}(S_{k,n}), \\
\lim_{W\to 0} \mathbb{E}(S_{k,n}^W) &= \mathbb{E}(S_{k,n}) - \sum_{i=k}^{n} \binom{i}{2}^{-1} Z_{\mathrm{k,i}}^{\mathrm{n}} (1 - \binom{i}{2} W), \\
&= W \sum_{i=k}^{n} Z_{\mathrm{k,i}}^{\mathrm{n}}, \\
&= W.
\end{aligned}
$$

As we allow $W \to \infty$ the *W-Truncated coalescent* converges to results expected under standard coalescent theory, and when $W \to 0$ the time to k^{th} coalescence is limited by W.

We now expand $\mathbb{E}(T_{k,n}^W) = \mathbb{E}(S_{k,n}^W) - \mathbb{E}(S_{k+1,n}^W)$ and obtain

$$
\begin{aligned}
\mathbb{E}(T_{k,n}^W) &= \left(\mathbb{E}(S_{k,n}) - \sum_{i=k}^{n} \binom{i}{2}^{-1} Z_{\mathrm{k,i}}^{\mathrm{n}} e^{-\binom{i}{2}W} \right) - \left(\mathbb{E}(S_{k+1,n}) - \sum_{i=k+1}^{n} \binom{i}{2}^{-1} Z_{\mathrm{k+1,i}}^{\mathrm{n}} e^{-\binom{i}{2}W} \right), \\
&= \mathbb{E}(T_{k,n}) - \binom{k}{2}^{-1} Z_{\mathrm{k,k}}^{\mathrm{n}} e^{-\binom{k}{2}W} - \sum_{i=k+1}^{n} \binom{i}{2}^{-1} e^{-\binom{i}{2}W} (Z_{\mathrm{k,i}}^{\mathrm{n}} - Z_{\mathrm{k+1,i}}^{\mathrm{n}}), \\
&= \mathbb{E}(T_{k,n}) - \binom{k}{2}^{-1} Z_{\mathrm{k,k}}^{\mathrm{n}} e^{-\binom{k}{2}W} - \sum_{i=k+1}^{n} \binom{i}{2}^{-1} e^{-\binom{i}{2}W} Z_{\mathrm{k+1,i}}^{\mathrm{n}} \left(\frac{\binom{k}{2}}{\binom{k}{2} - \binom{i}{2}} - 1 \right), \\
&= \mathbb{E}(T_{k,n}) - \binom{k}{2}^{-1} Z_{\mathrm{k,k}}^{\mathrm{n}} e^{-\binom{k}{2}W} - \sum_{i=k+1}^{n} \binom{k}{2}^{-1} e^{-\binom{i}{2}W} Z_{\mathrm{k+1,i}}^{\mathrm{n}} \left(\frac{\binom{k}{2}}{\binom{k}{2} - \binom{i}{2}} \right), \\
&= \mathbb{E}(T_{k,n}) - \binom{k}{2}^{-1} Z_{\mathrm{k,k}}^{\mathrm{n}} e^{-\binom{k}{2}W} - \sum_{i=k+1}^{n} \binom{k}{2}^{-1} e^{-\binom{i}{2}W} Z_{\mathrm{k,i}}^{\mathrm{n}}, \\
&= \binom{k}{2}^{-1} \left(1 - \sum_{i=k}^{n} Z_{\mathrm{k,i}}^{\mathrm{n}} e^{-\binom{i}{2}W} \right),
\end{aligned} \tag{10}
$$

where we use the result from conventional coalescent theory that $\mathbb{E}(T_{k,n}) = \mathbb{E}(S_{k,n}) - \mathbb{E}(S_{k+1,n}) = \binom{k}{2}^{-1}$.

S1.3.3 Expected branch lengths

We aim to get a distribution of the expected number of mutations found at a given heteroplasmy. To do this we first need to find the expected branch length of all branches in the tree which carry b descendants ($\mathbb{E}(L_{b,n}^W)$). At a level with k lineages,

we wish to know the probability that any one of those lineages will carry b descendants where $1 \leq b \leq n-k+1$. To do this we consider the vector of the number of descendants of the k lineage, $(J_1^k, ..., J_k^k)$ with $J_m^k \geq 1$. This vector is uniformly distributed over all vectors where $\sum_{m=1}^{k} J_m^k = n$. The number of such vectors is $\binom{n-1}{k-1}$. The number of these vectors for which a single lineage m carries b descendants is equal to the number of ways the remaining $n-b$ descendants can be split into $k-1$ non-empty groups, which is a known result from combinatorics of $\binom{n-b-1}{k-2}$. This leaves the probability of a lineage carrying b descendants $P(J_m^k = b) = \binom{n-b-1}{k-2}/\binom{n-1}{k-1}$. Given there are k lineages at this level, and the level is present for a time $\mathbb{E}(T_{k,n}^W)$, we find the contribution from all levels to the length of all branches carrying b descendants to be:

$$\mathbb{E}(L_{b,n}^W) = \sum_{k=2}^{n} \mathbb{E}(T_{k,n}^W) \cdot k \cdot \frac{\binom{n-b-1}{k-2}}{\binom{n-1}{k-1}} \tag{11}$$

Substituting in the expected times to coalescence gives us a formula of

$$\mathbb{E}(L_{b,n}^W) = \sum_{k=2}^{n} \binom{k}{2}^{-1} \left(1 - \sum_{j=k}^{n} Z_{\mathrm{k,j}}^{\mathrm{n}} e^{-\binom{j}{2}W}\right) \cdot k \cdot \frac{\binom{n-b-1}{k-2}}{\binom{n-1}{k-1}}.$$

Using the identity

$$\frac{\binom{n-b-1}{k-2}}{\binom{n-1}{k-1}} \frac{1}{k-1} = \frac{\binom{n-k}{b-1}}{\binom{n-1}{b}} \frac{1}{b},$$

we can get the most simplified form of the $\mathbb{E}(L_{b,n}^W)$:

$$\mathbb{E}(L_{b,n}^W) = \frac{2}{b}\left(1 - \sum_{k=2}^{n} \frac{\binom{n-k}{b-1}}{\binom{n-1}{b}} \cdot \sum_{j=k}^{n} Z_{\mathrm{k,j}}^{\mathrm{n}} e^{-\binom{j}{2}W}\right) \qquad b < n \tag{12}$$

To find the extended site frequency spectrum we must identify the length of the 'root' of the coalescent tree $\mathbb{E}(L_{n,n}^W)$. This is done by finding the difference between $S_{2,n}^W$ and W.

$$\begin{aligned}\mathbb{E}(L_{n,n}^W) &= W - \mathbb{E}(S_{2,n}^W) \\ &= W - (\mathbb{E}(S_{2,n}) - \sum_{j=2}^{n} \binom{j}{2}^{-1} Z_{2,\mathrm{j}}^{\mathrm{n}} e^{-\binom{j}{2}W}) \\ &= W - 2(1 - \frac{1}{n}) + \sum_{j=2}^{n} \binom{j}{2}^{-1} Z_{2,\mathrm{j}}^{\mathrm{n}} e^{-\binom{j}{2}W}\end{aligned} \tag{13}$$

Combining these results we get:

$$\mathbb{E}(L_{b,n}^W) = \begin{cases} \frac{2}{b}\left(1 - \sum_{k=2}^{n} \frac{\binom{n-k}{b-1}}{\binom{n-1}{b}} \cdot \sum_{j=k}^{n} Z_{\mathrm{k,j}}^{\mathrm{n}} e^{-\binom{j}{2}W}\right) & b < n \\ W - 2(1 - \frac{1}{n}) + \sum_{j=2}^{n} \binom{j}{2}^{-1} Z_{2,\mathrm{j}}^{\mathrm{n}} e^{-\binom{j}{2}W} & b = n \end{cases} \tag{14}$$

We see from equation 14 that while the expected branch length for heteroplasmic mutations reach a steady state as $W \to \infty$, the equivalent for homoplasmic mutations will constantly increase as time goes on. This is because homoplasmic mutations have no avenue to be lost from the population due to the infinite sites assumption, whereas heteroplasmic mutations are either lost or fix at homoplasmy. This constant accumulation of homoplasmies is what allows us to use the extended site frequency spectrum to infer a tissue's age when its heteroplasmies have already reached equilibrium. For an alternate derivation of this distribution, consult section 3.8.1 of Green 2022 (*13*).

S1.4 Distribution of number of mutations in a single cell

The distribution of the number of mutations M that occur on a given length of a coalescent tree, L, is distributed as a Poisson with rate θL, where $\theta = Nu/2$. We wish to find the probability of a given number of mutations at a heteroplasmy b/n, $M_{b,n}$ given the truncation time, W, and the mutation rate, θ, which can be found by integrating over the probability distribution of all potential branch lengths of the tree.

$$P(M_{b,n} = m|\theta, W) = \int_0^{\lfloor \frac{n}{b} \rfloor W} \frac{1}{m!}\left(\theta L_{b,n}^W\right)^m \exp\left(-\theta L_{b,n}^W\right) P(L_{b,n}^W|W) \mathrm{d}L_{b,n}^W, \tag{15}$$

where $L_{b,n}^W$ is the total branch length of the coalescent tree which carries b descendants. Doing this integral analytically is computationally intractable, so we turn to Monte-Carlo integration to approximate the integral. In brief, we sample S

coalescent trees under the truncated coalescent process parameterized by W, take the lengths $(L^W_{b,n})_i$ from these samples and evaluate:

$$P(M_{b,n} = m|\theta, W) \approx \frac{1}{S}\sum_{i=1}^{S}\frac{1}{m!}\Big(\frac{\theta(L^W_{b,n})_i}{2}\Big)^m \exp\Big(-\frac{\theta(L^W_{b,n})_i}{2}\Big). \tag{16}$$

S1.5 Multiple cells

Now that we have $P(M_{b,n} = m|\theta, W)$ for a single cell we can use this for our likelihood of a set of C cells carrying $M^c_{b,n}$ mutations each. If $C_{m,b}$ is the number of cells with m mutations at heteroplasmy b/n, then:

$$P(\{M^c_{b,n}\}|\theta, W) = \prod_c P(M^c_{b,n}|\theta, W) \tag{17}$$

$$= \prod_m P(M_{b,n} = m|\theta, W)^{C_{m,b}}. \tag{18}$$

Looking at the number of mutations across all heteroplasmies we find the likelihood of data D to be:

$$P(D|\theta, W) = \prod_{b=1}^{n}\prod_{m=0}^{\infty} P(M_{b,n} = m|\theta, W)^{C_{m,b}}. \tag{19}$$

S1.6 Hierarchical Bayesian Inference

In order to account for potential inter-individual differences in mtDNA copy number, turnover rate, and mutation rate, as well as the variable number of bases surveyed in each sequencing experiment, we apply a hierarchical model structure to our inference. The conversion between proper time and the truncation time W is $W = \frac{2\ln 2}{Nt_{1/2}}\tau$. To account for differences between the copy number and mtDNA half-life of different individuals we will attempt to infer the mitochondrial ageing rate $\alpha_j = \frac{2\ln 2}{N_j t_{1/2,j}}$ for each individual j, such that an individual of age τ_j has a mitochondrial age $W_j = \alpha_j\tau_j$. We must also account for differences in copy number, N_j, mutation rate per base per replication, ν_j, and number of bases surveyed B_j which will all contribute to the coalescent mutation rate $\theta_j = N_j\nu_j B_j/2$. We will attempt to infer a parameter $\Theta_j = N_j\nu_j/2$ for each individual.

The hierarchical structure of the model will be as follows: each individual has two target inference parameters α_j, Θ_j which are drawn from population distributions $\alpha_j \sim \text{LogNormal}(\mu_\alpha, \sigma_\alpha)$, $\Theta_j \sim \text{LogNormal}(\mu_\Theta, \sigma_\Theta)$. The parameters μ_α and μ_Θ are the expected values of α_j and Θ_j, while σ_α and σ_Θ are the standard deviations of the logarithm of these two random variables.

For a set of K donors $X_1, ..., X_K$, each with C_d cells each, our final joint posterior for the hyperparameters will be:

$$P(\{\alpha_d\}, \{\theta_d\}, \mu_\alpha, \sigma_\alpha, \mu_\Theta, \sigma_\Theta|D) = \tag{20}$$

$$\left(\prod_{d=1}^{K}\prod_{b=1}^{n}\prod_{m=0}^{\infty} P(M_{b,n} = m|\theta_d, W_d)^{C^d_{m,b}} P(W_d|\mu_\alpha, \sigma_\alpha, \tau_d) P(\theta_d|\mu_\Theta, \sigma_\Theta, B_d)\right) \times P(\mu_\alpha, \sigma_\alpha, \mu_\Theta, \sigma_\Theta) \tag{21}$$

S1.7 Comparison to the cryptic site frequency spectrum

The similarity between the cryptic mutations we identify from experiment and the model of *de novo* mutations that occur post-mitotically is affected by any replication those cells experience throughout an organisms life, as well as by tissue sampling effects. We limit ourselves to studying tissue types which undergo relatively slow turnover: the Enge dataset (*14*) is made up from majority alpha, acinar, ductal, and beta cells, so as from (*15*) we expect ductal cells to be largely non-proliferative, and alpha, acinar and beta cells to be 83 %, 24 % and 61 % non-proliferative respectively at the time points we consider. Ref. (*15*) uses neurons as the reference slow tissue; we consider neural datasets (*16–18*). Our last dataset (*19*) is taken from the retinal epithelium, which has cellular lifespan equivalent to that of neurons (*15*).

In the event that a cell has undergone additional rounds of cellular replication (and yet has no sister cells contained within the sample) the cell will have a site frequency spectrum with a higher average heteroplasmy than a cell with the same mtDNA birth-death rate and no cellular replication: for the cell that replicates, the theory we outline above is still broadly applicable, but the interpretation of the parameters associated with turnover will be different.

As cryptic mutations are defined as those that occur in only one cell *within the dataset*, we could classify a mutation which occurs late in development or after a cell turnover event as cryptic if we only sample one cell in which this mutation occurs. Our sample-specific definition of cryptic also prevents us from considering post-mitotic *de novo* mutations which independently recur multiple times in an individual, however, in the datasets used through this paper $< 1\,\%$ of mutations will be recurrent (simplifying and assuming the mutation probability is constant along the length of mtDNA).

S2 Supplementary Discussion: Model inferences

S2.1 Comparison to simulation

We now show that the model specified above can properly predict the mitochondrial age, W, and mutation rate, θ, from data generated using simulations of the Moran model using the parameters: $t_{1/2} = 15$ days (*9*, *10*), $\nu = 4 \times 10^{-8}$ per base per replication (*11*) and $N = 1000$ (*12*). We do not initially impose a hierarchical structure on our model, as we first wish to verify that our simple likelihood can recapitulate simulation parameters. Simulating 300 different cells for different lengths of time, and sampling 200 mtDNA from each cell, we apply the model to the resulting SFS with a uniform prior for $W \in [0, 5]$ and $\theta \in [10^{-2}, 10^{2}]$. Given that when we analyse real data we filter our heteroplasmies below 10 %, we apply the same filter to this simulated data. This makes the final likelihood:

$$P(D|\theta, W) = \prod_{b=20}^{200} \prod_{m=0}^{\infty} P(M_{b,n} = m|\theta, W)^{C_{m,b}}. \quad (22)$$

We see in tables S1 and S2 that the true W and θ lie inside the 95 % credible interval of the marginal posteriors inferred for W and θ for each time point. The full marginals for W and θ are shown in Fig. S3 and we also show the SFS predicted from the maximum a posteriori (MAP) estimate of W compared to the simulated (Figure S4). From this we can have confidence that our inference can credibly infer parameters from realistic amounts of data.

Age Simulated (Years)	True W Value	95 % Credible Interval	MAP Estimate W
5	0.17	(0.07, 0.18)	0.12
10	0.34	(0.20, 0.36)	0.27
25	0.84	(0.64, 0.86)	0.78
50	1.68	(1.5, 1.94)	1.67
75	2.52	(2.23, 2.77)	2.49
100	3.36	(3.32, 4.08)	3.65

Table S1: The credible intervals and MAP estimate of W for different ages and their associated W values.

Age Simulated (Years)	True θ Value	95 % Credible Interval	MAP Estimate θ
5	0.33	(0.21, 2.04)	0.58
10	0.33	(0.27, 0.65)	0.42
25	0.33	(0.28, 0.41)	0.34
50	0.33	(0.28, 0.35)	0.32
75	0.33	(0.30, 0.38)	0.35
100	0.33	(0.28, 0.35)	0.32

Table S2: The credible intervals and MAP estimate of θ for different ages and their associated θ value.

S2.2 Hierarchical simulation comparison

To confirm that the parameters of the hierarchical model as outlined in section S1.6 can be recovered using inference, we now simulate 10 donors with different ages between 10 and 100 years old, with parameters $\alpha_d \sim \text{LogNormal}(\mu_\alpha, \sigma_\alpha)$, $\Theta_d \sim \text{LogNormal}(\mu_\Theta, \sigma_\Theta)$ where $\mu_\alpha = 0.03$, $\sigma_\alpha = 0.3$, $\mu_\Theta = 10^{-5}$, and $\sigma_\Theta = 0.5$. Each donor had a random number of bases from the genome sampled, $B_d \in \text{Uniform}(9000, 11000)$, and a random number of cells $C_d \in \text{Uniform}(200, 400)$. We find that the model is able to infer the hyperparameters, as well as the individual donor parameters (see table S3, Fig. S5).

Parameter	True Value	95 % Credible Interval	MAP Estimate
μ_α	0.03	(0.028, 0.038)	0.033
σ_α	0.3	(0.06, 0.42)	0.15
μ_Θ	10^{-5}	$(6.61 \times 10^{-6}, 1.51 \times 10^{-5})$	9.55×10^{-6}
σ_Θ	0.5	(0.28, 0.35)	0.32

Table S3: The credible intervals and MAP estimate for the hyperparameters. The true hyperparameters are contained in the 95 % confidence interval in all cases.

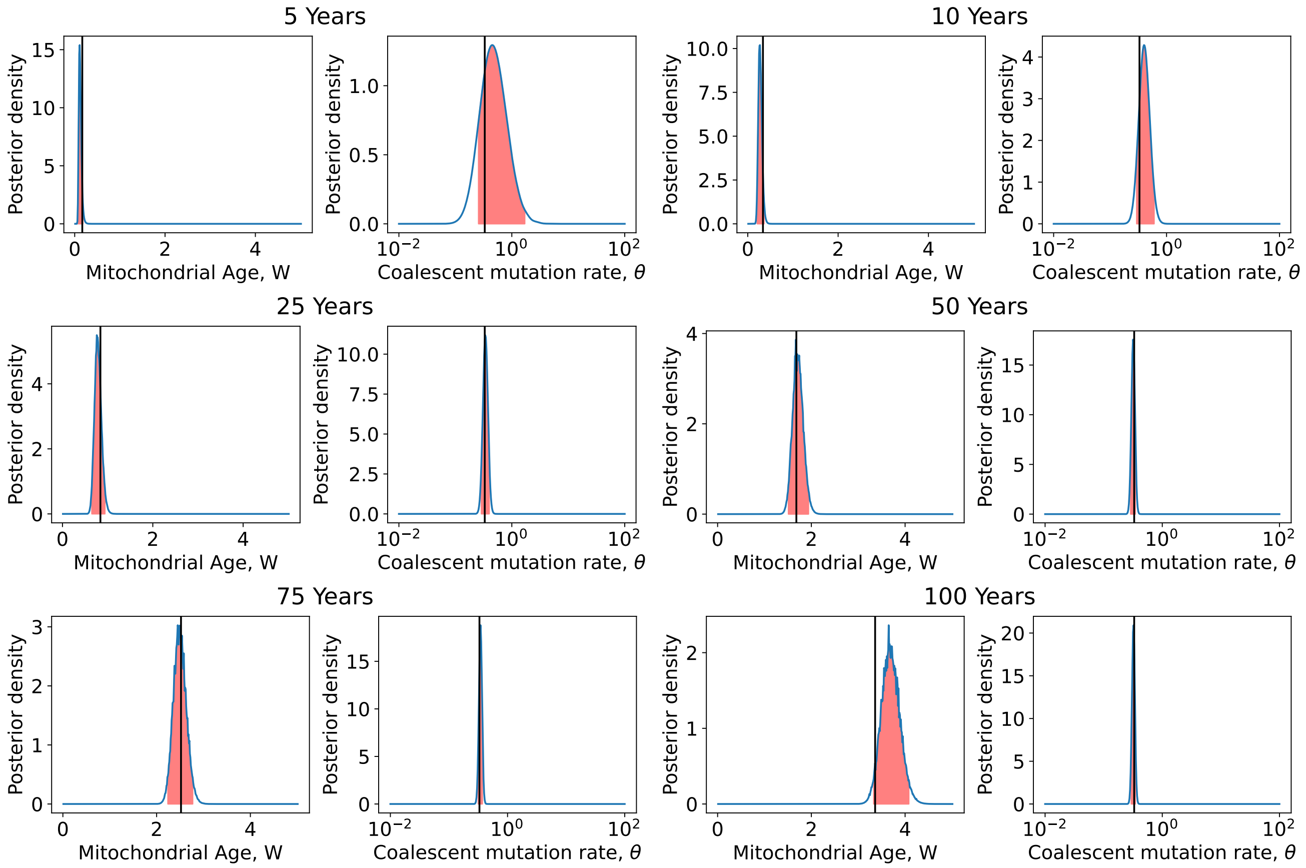

Supplementary Figure S3: **The posterior distribution of the mitochondrial age W and coalescent mutation rate θ found for each time point.** We find for every donor that the true W and θ values (indicated with the black vertical line) lie inside the 95 % credible interval, shown in red.

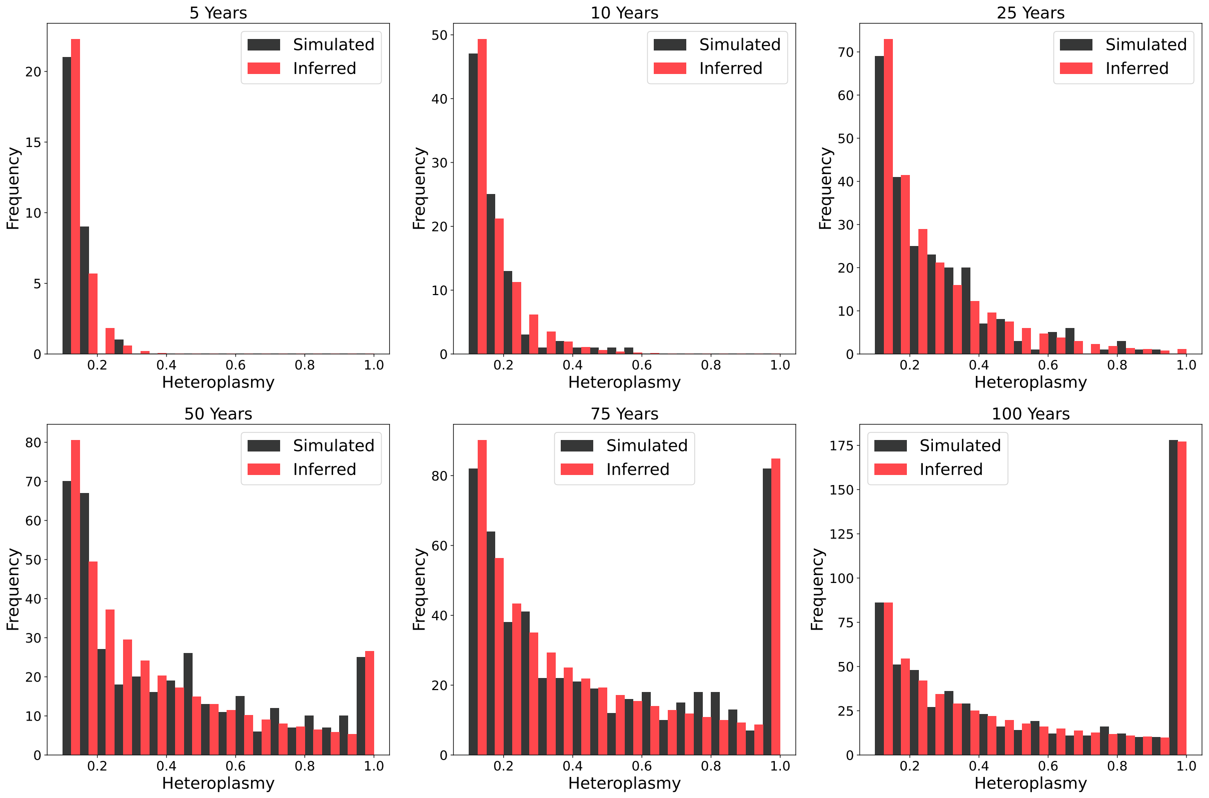

Supplementary Figure S4: **The predicted expected cSFS for the MAP estimates of W and θ, compared to the simulated cSFS for different ages.** We find that simulated and inferred cSFS match well for different ages.

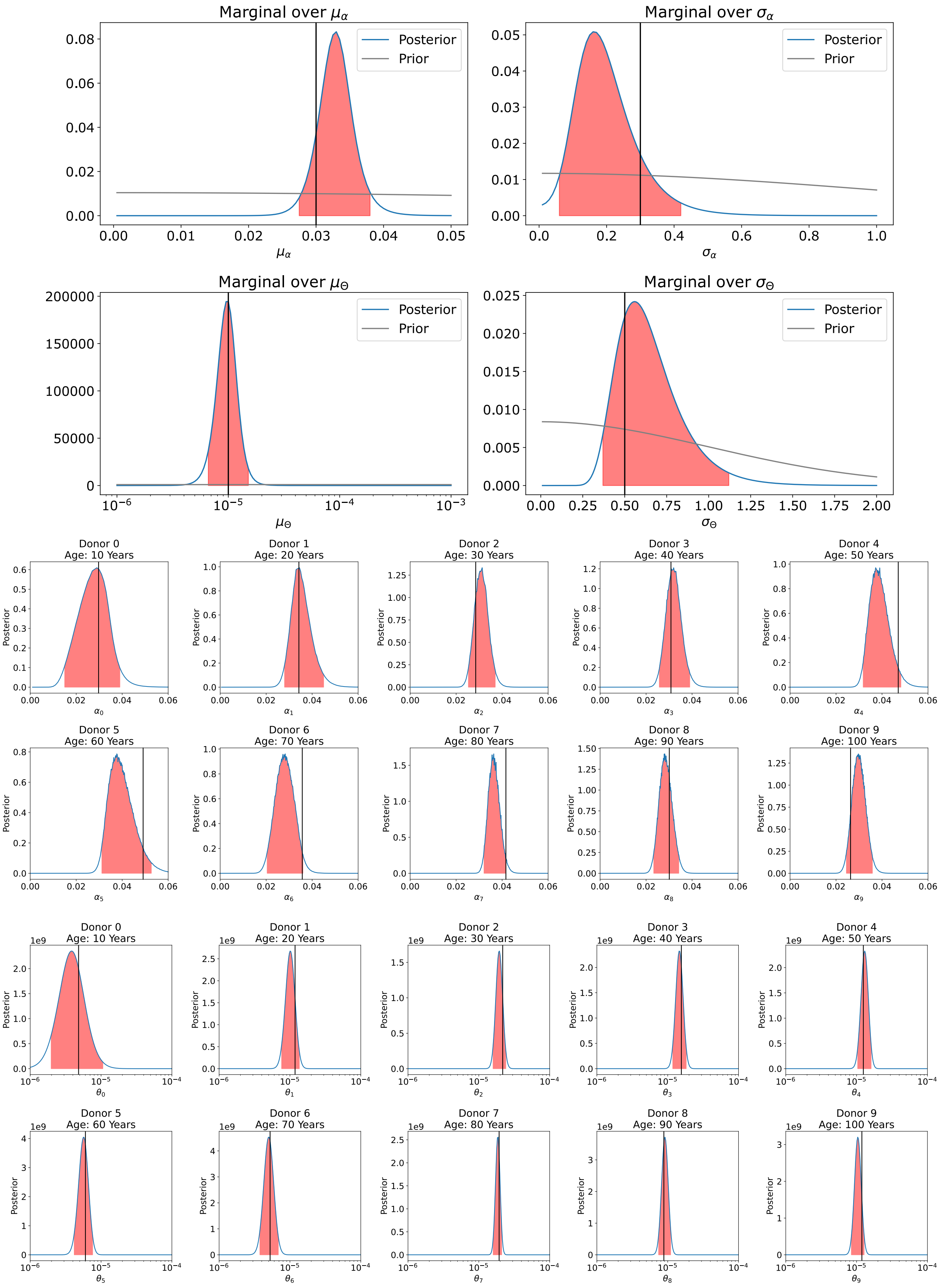

Supplementary Figure S5: **The marginal posterior distributions of the model parameters.** The 95 % confidence intervals are marked in red, with the true parameter values shown with a black line. We see that the true value is contained by the 95 % confidence interval in all cases.

S2.3 Model fitting to Data

We have established that the model is able to recapitulate parameters from simulated data accurately, and we now wish to apply it to our collected data. With this we have two goals - to show that mitochondrial age W, as inferred from the data, can be used as a marker of tissue age, and to find an estimate of the average mitochondrial mutation rate. We assume that all mutations which could be generated during sample and library preparation are found at heteroplasmies below 10% (see section S6.2), and that the sample size n should correlate with the average number of mtDNA a mutation is found from. Following the arguments from section S6.2 we expect there to be 7 reads for every true mtDNA sequenced. The average read depth of mutations is ≈ 640 reads and so we use $n = 90$ as the number of true mtDNA in our sample from the mtDNA population of the cell, though we find equivalent results for other values of n. In order to fit the data using our discrete likelihood, we round our heteroplasmy to values of $h = \frac{i}{90}$ where $i \in [1, .., 90]$, and then compute the full likelihood given all the data. The final likelihood form is:

$$P(D|\theta, W) = \prod_{b=9}^{90} \prod_{m=0}^{\infty} P(M_{b,n} = m|\theta, W)^{C_{m,b}}. \tag{23}$$

where $C_{m,b}$ is the number of cells with m mutations at heteroplasmy $\frac{b}{90}$.

S2.4 Fitting to human data

For the human data we picked broad hyperprior distributions of μ_α ~Half-Normal(0.1), μ_Θ ~Half-Normal(0.01), σ_α ~Half-Normal(1), σ_Θ ~Half-Normal(1). Shown in Fig. 1h are the 95 % confidence intervals of the inferred mitochondrial age of every donor, which, as expected, increases with biological age. The fit shown on this plot is the MAP estimate of μ_α and its 95 % confidence interval. In Fig. 1i we show the marginal distribution of μ_Θ, scaled by an estimate of mtDNA copy number, $N = 1000$ (*12*) such that we are seeing the posterior of the median mutation rate per base per replication, μ_ν. The full posteriors of both the hyperparameters and the donor specific parameters are shown in Figs. S6, S7, S8. We find that similar parameter values (though with broader posteriors) are recapitulated when the model is fit only to the most abundant cell type from the Enge pancreas dataset (*14*) (see Fig. S26c).

Using our MAP estimates of W and θ for each donor, we can compare the expected site frequency spectrum for these values to the observed (Figure S9). We find that there is broad agreement between our expected site frequency spectra and those observed. Adding an explicit error model of sequencing and PCR to the likelihood, and allowing thresholds to vary based on the data quality would improve the inference, though the trends seen would likely remain unchanged. The fits presented in the main text for changes in relative probability of high heteroplasmy mutations (Fig. 1j) and for changes in the number of homoplasmic mutations (Fig. 1k-m) are done using the MAP estimates of μ_α and μ_Θ, corresponding to the median of population distribution.

Parameter	Data used	MAP estimate	95 % Credible Interval
μ_α	All human (*14*, *19*, *20*)	0.036	(0.029, 0.045)
μ_α	Mouse liver (*21*)	0.456	(0.300, 0.768)
μ_α	Mouse pancreas (*21*)	0.252	(0.156, 0.528)
μ_Θ	All human (*14*, *19*, *20*)	2.29×10^{-5}	$(1.38 \times 10^{-5}, 4.79 \times 10^{-5})$
μ_Θ	Mouse liver (*21*)	2.19×10^{-4}	$(1.58 \times 10^{-4}, 3.31 \times 10^{-4})$
μ_Θ	Mouse pancreas (*21*)	1.51×10^{-4}	$(9.55 \times 10^{-5}, 2.51 \times 10^{-4})$
σ_α	All human (*14*, *19*, *20*)	0.42	(0.33, 0.67)
σ_α	Mouse liver (*21*)	0.54	(0.39, 1.14)
σ_α	Mouse pancreas (*21*)	0.67	(0.49, 1.42)
σ_Θ	All human (*14*, *19*, *20*)	1.25	(0.99, 1.82)
σ_Θ	Mouse liver (*21*)	0.34	(0.22, 0.85)
σ_Θ	Mouse pancreas (*21*)	0.31	(0.16, 1.28)

Table S4: The credible intervals and MAP estimate for the hyperparameters for all datasets. We see that mice have significantly higher mitochondrial ageing rate, μ_α, and coalescent mutation rate, μ_Θ.

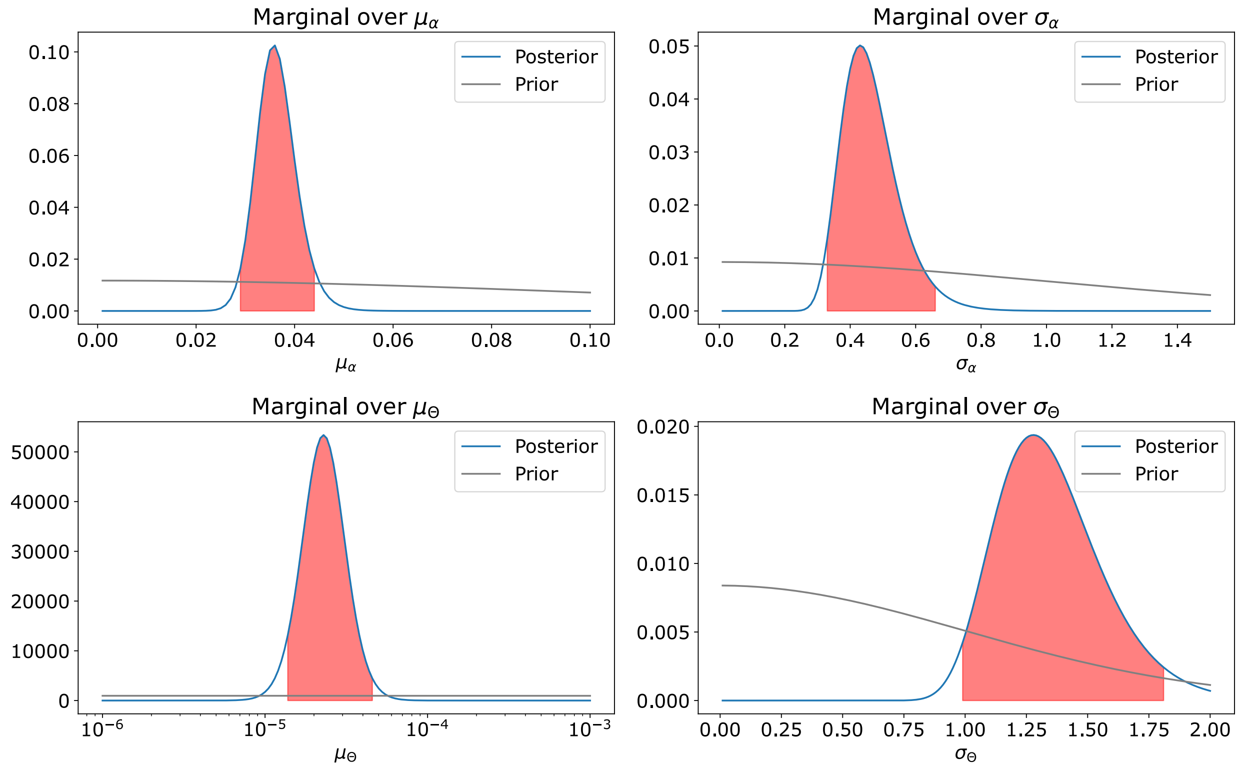

Supplementary Figure S6: **Unnormalised posteriors of the human hyperparameters.** Marked in red are the 95 % confidence intervals. The large inferred variance on the mutation rate, σ_Θ, is driven by the youngest donors' large inferred mutation rate. Our model is set to assume no effects from development, and so we also perform the inference with the non-adult donors excluded and find most parameters remain the same, with σ_Θ reducing significantly (see Fig. S10 for full posteriors in this case).

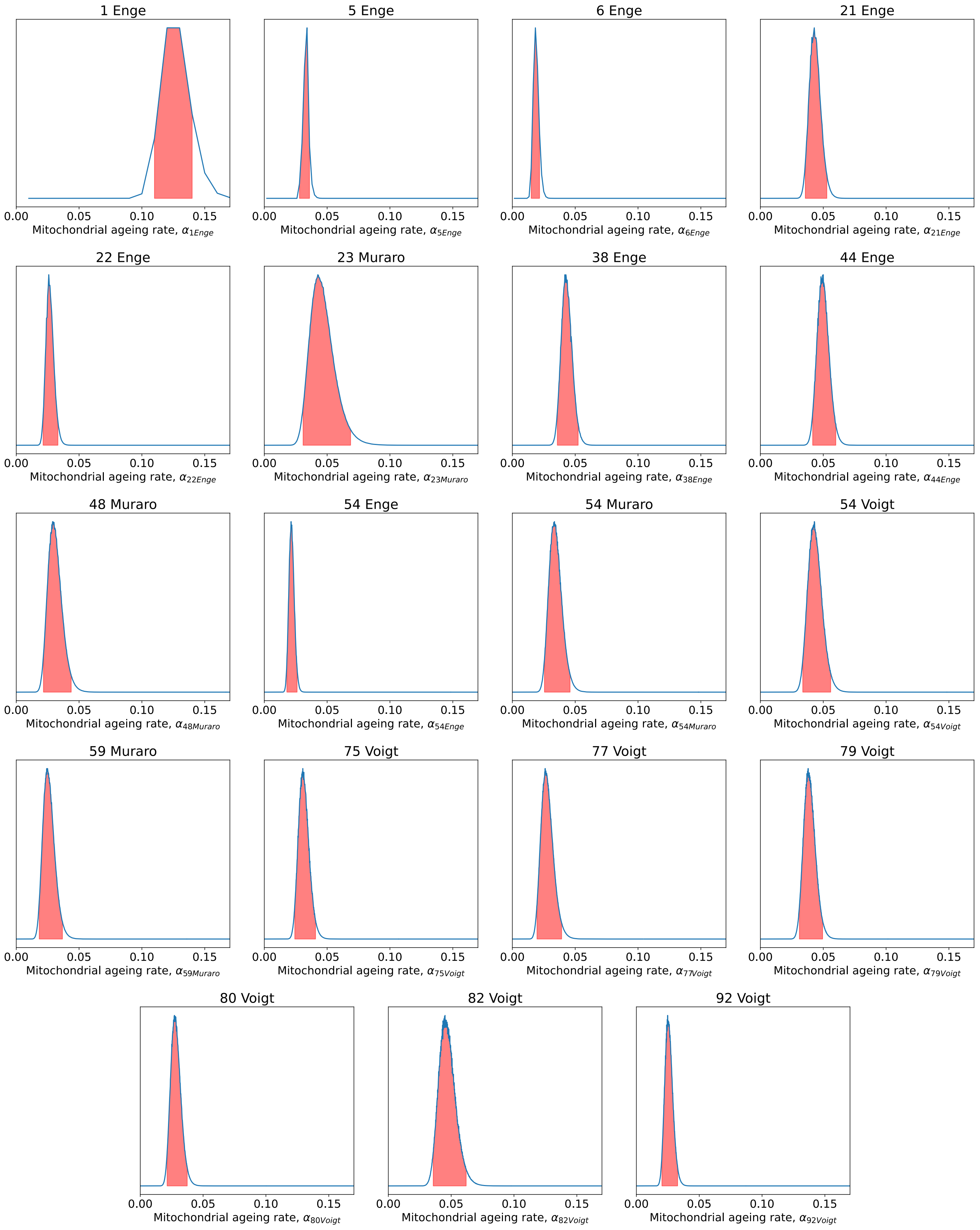

Supplementary Figure S7: **Unnormalised posteriors of the donor specific mitochondrial ageing rates.** Marked in red are the 95 % confidence intervals. To convert these posteriors into effective mitochondrial ages (as seen in Fig. 1h, we multiply α_d by the donor age. Due to the grid evaluation of the posterior being over mitochondrial age, we see fewer evaluated points across the same range of α for younger donors.

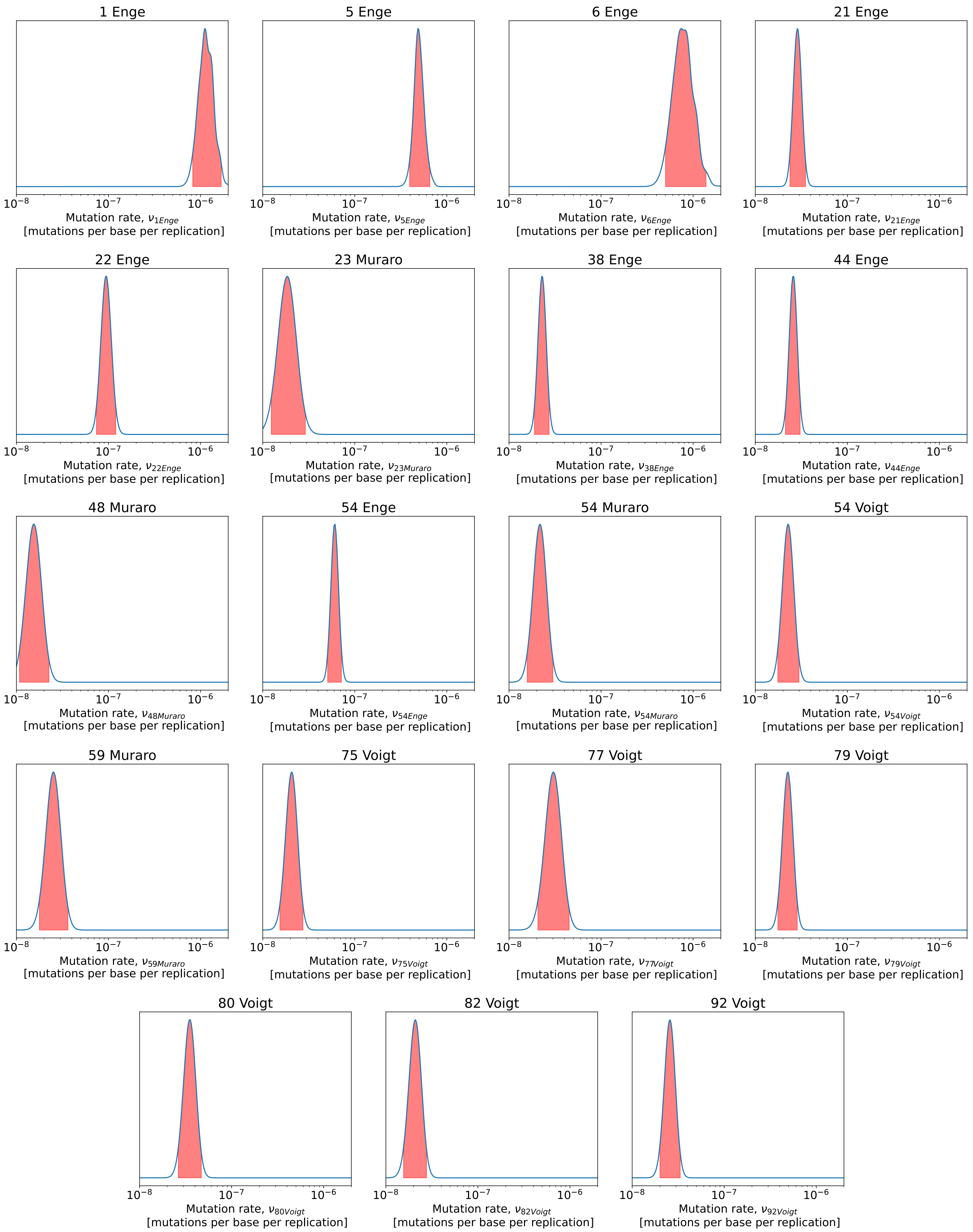

Supplementary Figure S8: **Unnormalised posteriors of the donor specific mutation rates.** Marked in red are the 95 % confidence intervals. We see that the youngest 3 donors have much higher inferred mutation rates that the other donors. This could be due to low heteroplasmy mutations being dominated by errors from PCR, and very few high heteroplasmy mutations being present which can be used to compensate due to their young age.

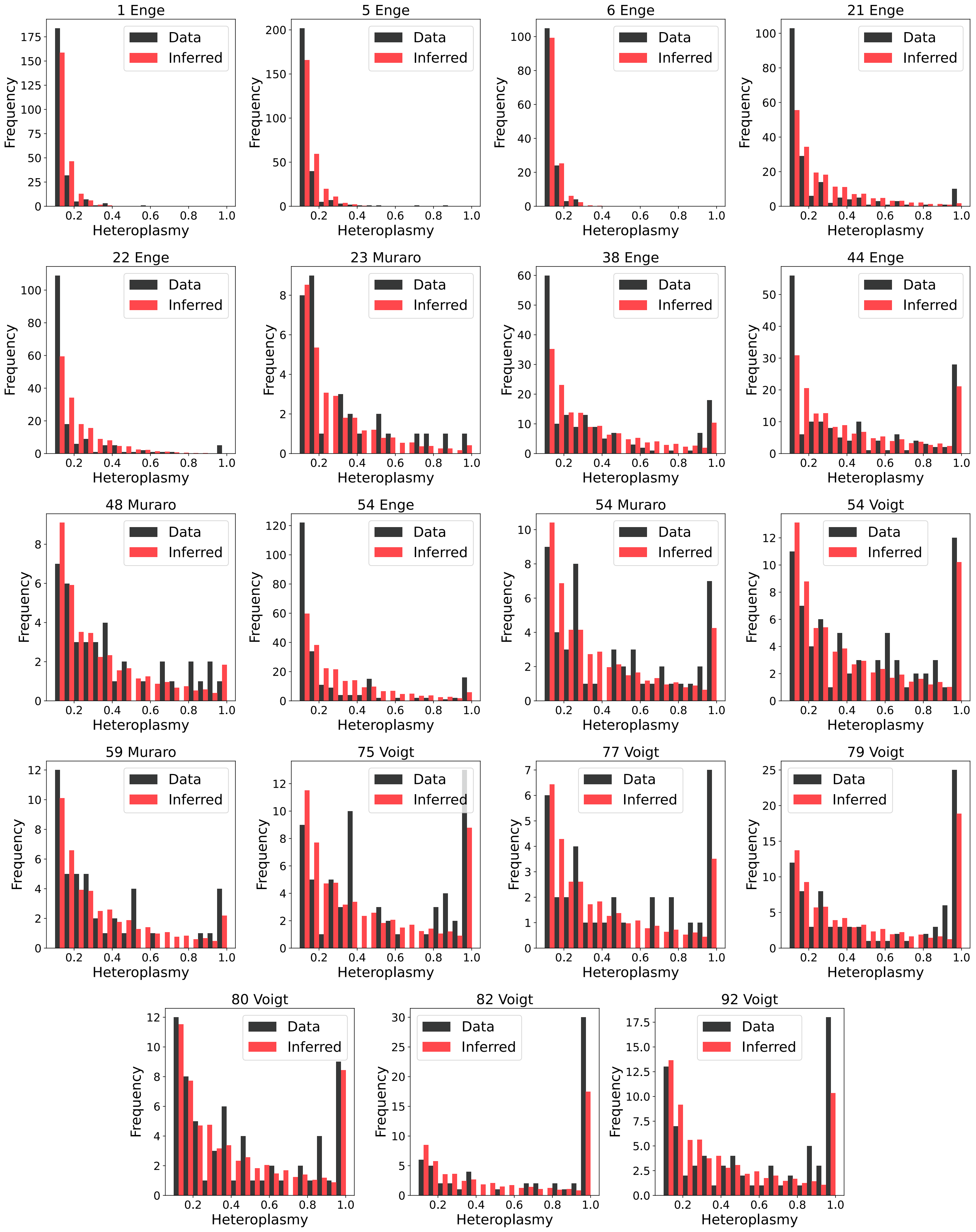

Supplementary Figure S9: **Comparison between the site frequency spectrum of our donors and the expected site frequency spectrum given our MAP estimate of W.** The title of each subplot in the format *Age (Years) Dataset*. We see that the model performs better for Muraro and Voigt datasets, possibly due to the additional rounds of PCR in the high depth Enge dataset introducing more errors at low heteroplasmies.

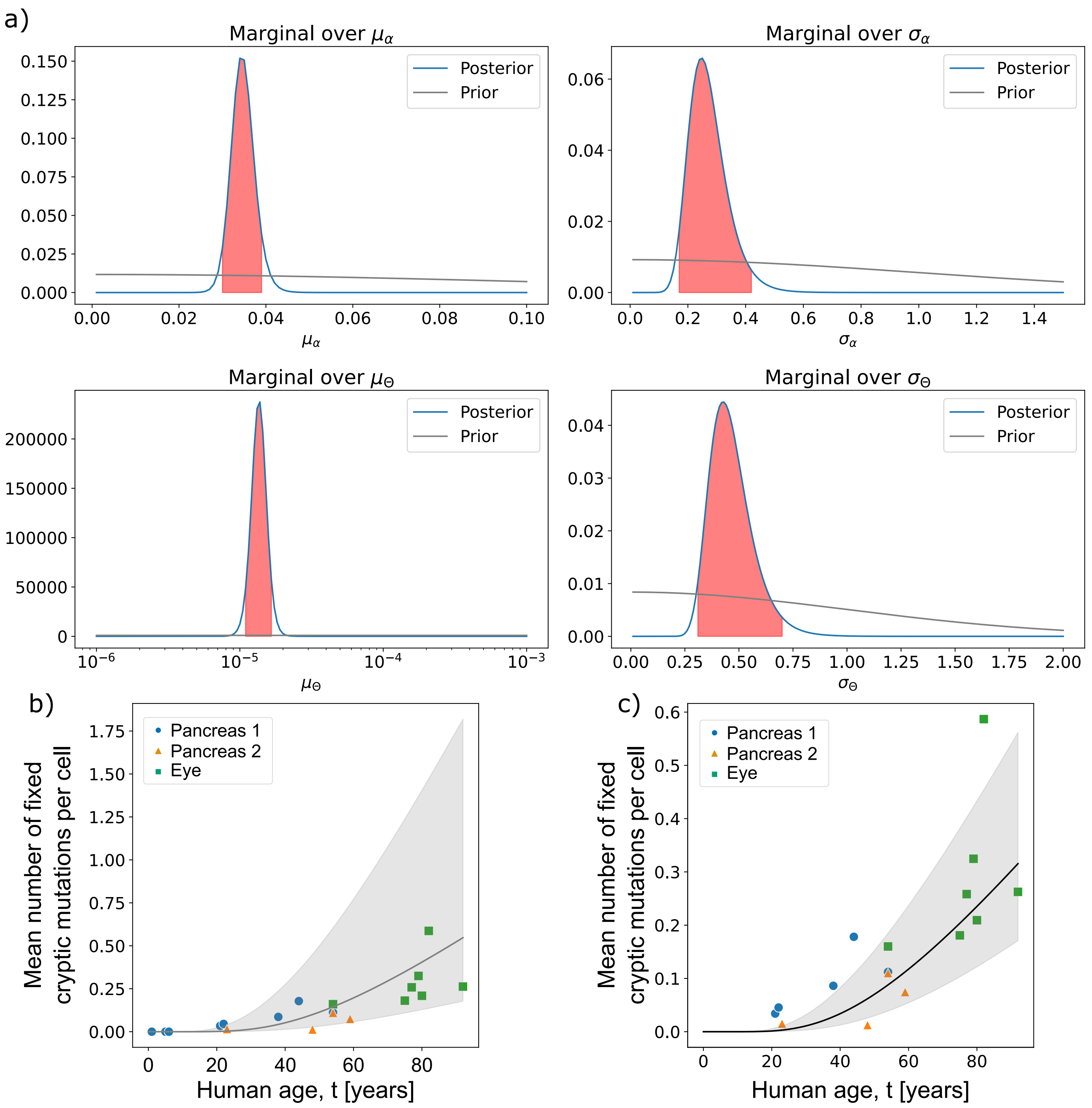

Supplementary Figure S10: **Exclusion of non-adult donors does not impact main trends.** In order to investigate if our results are driven by developmental effects, we repeated our inference after exclude the three non-adult donors. By excluding the youngest donors in the dataset, we see that posterior inferences tighten and trends remain unchanged, indicating our results are not driven by developmental effects. (a) The posteriors on the hyperparameters when the youngest donors are excluded from the dataset (see supplementary figure S6 for original fit). We find that the posterior inferences tighten slightly when these donors are excluded. These youngest donors carry few mutations, which can be achieved with a wide range of turnover rates when the mutation rate is low, whereas when only the older donors are included the parameters can be inferred more precisely. (b-c) Shown are the fits to the expected number of cryptic homoplasmic mutations. Marked in grey are the 95 % confidence intervals on the median of this fit. The fit in (b) is done using all donors, whereas in (c) we exclude the youngest 3 donors. We see that by excluding the youngest donors the confidence interval shrinks substantially. We note that these are not fits done only to the homoplasmic mutations, but to the full set of mutations across the cSFS.

S2.5 Fitting to mouse data

Because of the difference in proliferation rate between pancreas and liver tissue, we fitted the two mouse tissue types separately. For both mouse tissues we picked hyperprior distributions of μ_α ∼Half-Normal(1.2), μ_Θ ∼Half-Normal(0.01), σ_α ∼Half-Normal(1), σ_Θ ∼Half-Normal(1). We found that both the population mitochondrial ageing rate, μ_α, and the population coalescent mutation rate, μ_Θ of both tissues to be much higher than that of the human data (see table S4). Both parameters depend on multiple biological parameters such as copy number, turnover rate and mutation rate, and so while we see the net effect of any differences between humans and mice results in a faster mitochondrial ageing rate and a higher coalescent mutation rate, we cannot disambiguate what is causing this difference. The posterior inferences for the hyperparameters of mouse data are particularly broad due to the lack of sufficient aged data (see Fig. S11), however the difference between the mouse and human parameters are still evident.

S2.6 Investigating sex-specific effects of mitochondrial ageing

The human datasets we analysed all contained both male and female donors, and so in addition to jointly inferring the mitochondrial parameters, we set out to find if there is a difference between the mitochondrial ageing rates. By performing the inference on the 13 male and 7 female donors, we find no evidence for a difference in the mitochondrial ageing rates μ_α of men and women (Fig S12).

S2.7 Comparing diabetic to healthy tissue

To further demonstrate the applicability of our model to comparative studies, we analysed single cell RNA seq data taken from both healthy and diabetic pancreas tissue (*22*). We fit the model separately on mutations from 1977 cells from 8 healthy donors and 1732 cells from 6 diabetic donors, processed in the same way as the rest of our human data (see Methods). First we found that, for the healthy donors, we credibly recapitulated the parameter values that we found in table S4. We further found there was evidence for a difference in the mitochondrial ageing rate, with a 93 % probability that μ_α was lower for the diabetic donors (Fig. S13b). To increase the power of the study, we merged the healthy donors from (*22*) with one other healthy human pancreas dataset already analysed (*14*) and found that the trend was preserved, with an improved 99 % probability that the mitochondrial ageing rate of the pancreas is slower for patients with diabetes compared to controls (Fig. S13c).

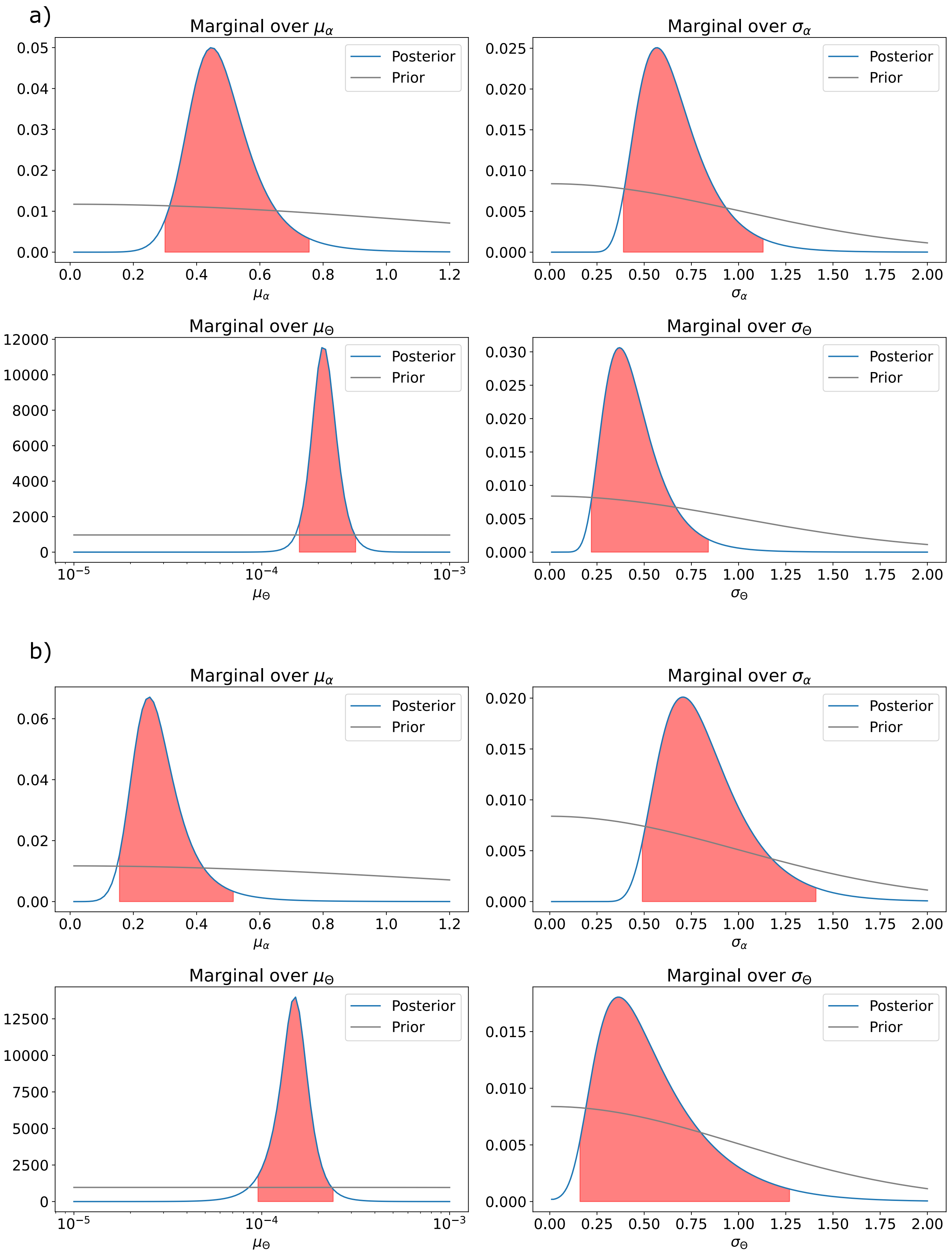

Supplementary Figure S11: **The posteriors on the hyperparameters for both mouse tissues.** We see that for both tissues the posteriors are wider than those inferred for the human datasets, due to a reduced amount of single cell data from these tissues. a) The marginal posterior distributions for the mouse liver. b) The marginal posterior distributions for the mouse pancreas.

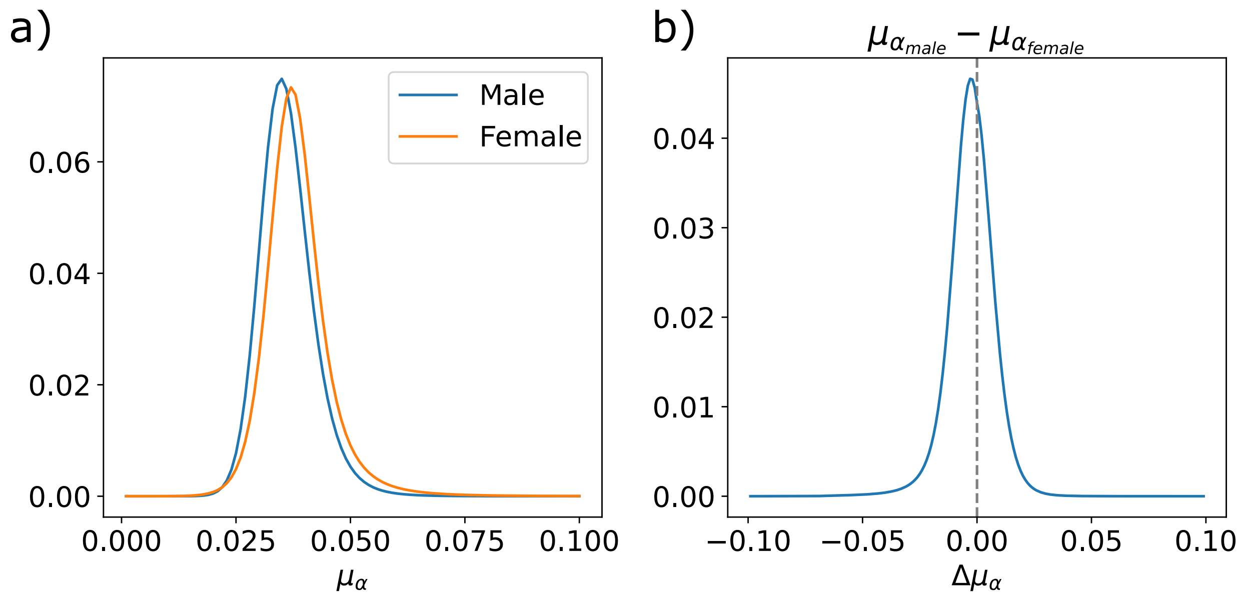

Supplementary Figure S12: **There is no marked difference in the mitochondrial ageing rate of men and women.** a) The marginal posterior distributions of the mitochondrial ageing rate, μ_α. b) The distribution of the difference in μ_α between the male and female human donors. There is no evidence of a shift in this ageing rate.

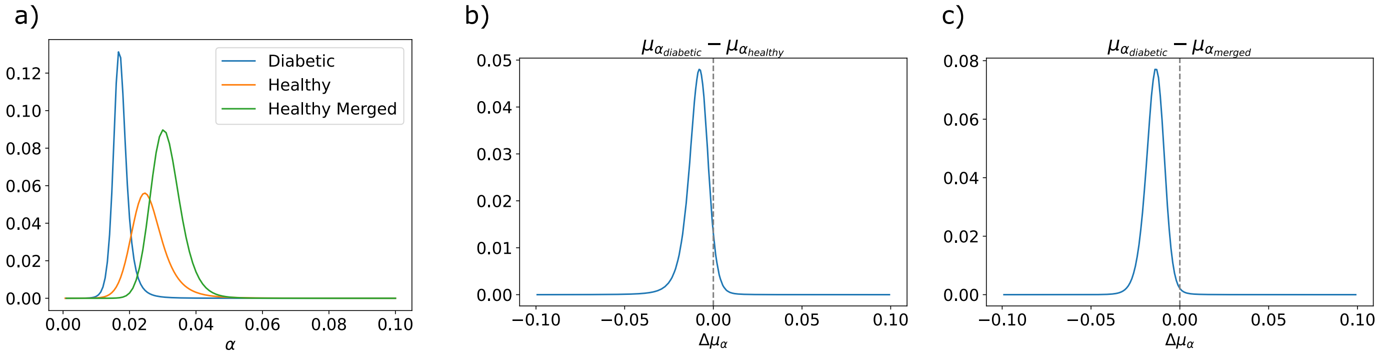

Supplementary Figure S13: **Evidence that diabetic pancreas tissue has a decreased mitochondrial ageing rate compared to healthy tissue.** a) The marginal posterior distributions of the mitochondrial ageing rate, μ_α. Shown are the posteriors on the diabetic donors (*22*), the healthy donors (*22*), and the healthy donors merged with data taken from healthy human pancreas from (*14*). b) The distribution of the difference in μ_α between the diabetic and healthy tissue from (*22*). 93 % of the probability mass lies below zero. c) When the healthy data (*22*) was merged with another healthy pancreas dataset (*14*), we found an increased effect on the value of μ_α. For this merged difference, 99 % of the probability mass lies below zero, raising the hypothesis of a difference in ageing rate between healthy and diabetic pancreas.

S3 Supplementary Discussion: Fisher's method for DEG identification

S3.1 Fisher's method for donors

In the maintext, we identified differentially expressed genes (DEGs) aggregated for all cells. This has the advantage that even modest shifts in gene expression are identifiable. It comes with the disadvantage, however, that confounding factors might create stronger shifts than actually present. While the consistency of our significant GO terms across different organs, species, sequencing techniques, and ages (see Fig. 3 in the main text) suggests that the aggregate approach we use in the maintext is appropriate, we nonetheless, here, present a complementary approach to eliminate the possibility that the DEGs that we identified in the main manuscript are exclusively age-dependent genes. For this, we calculate DEGs for each donor separately and then aggregate the p-values of differential expression in a 'meta'-analysis. Specifically, for a gene with p_d indicating the p-value of differential expression for donor d, we aggregate these p-values with Fisher's combined probability test to a test statistic

$$S_F = -2 \sum_{d \in \text{donor}} \ln p_d \,, \tag{24}$$

where S_F follows a χ^2 distribution, which we may lookup to obtain a p-value.

We use this approach for the Enge (human pancreas data) and the two mice data sets (liver and pancreas). This means that *we only make comparisons between cells which have the same age* (in the narrow sense of all the cells coming from the same individual). For all three datasets we obtain significant genes that are differentially expressed (after multiple-testing correction). We then perform a GO-term enrichment analysis for the obtained DEGs and demonstrate that we obtain similar GO-terms (see Fig. S14).

Finding similar GO terms enriched indicates that the identified biological perturbations are not predominantly driven by a difference between donors. Rather, it leaves open the possibility that mitochondrial mutation load could induce a genetic perturbation that is identifiable at the resolution of each donor.

S3.2 Fisher's method for cell types

Similarly to the 'meta'-analysis in Subsection S3.1 that only compares cells of the same donor, we can also perform a analysis that only compares cells of the same cell type. Specifically, for a gene with p_c indicating the p-value of differential expression for cell type c, we aggregate these p-values with Fisher's combined probability test to a test statistic

$$S_F = -2 \sum_{c \in \text{cell type}} \ln p_c \,, \tag{25}$$

where S_F follows a χ^2 distribution, which we may lookup to obtain a p-value.

Here, we analyse the Human pancreas data (Enge), for which the authors provide annotations that associate each cell with one of seven cell types ('acinar', 'alpha', 'beta', 'delta', 'ductal', 'mesenchymal', and 'unsure'). Using this method, we find 628 differentialy expressed genes after multiple-testing correction. We then perform a GO-term enrichment analysis for the obtained DEGs and demonstrate that we obtain similar GO-terms to the maintext analysis (see Fig. S14). This indicates that the mitochondrial mutation load could induce a genetic perturbation that is identifiable at the resolution of each cell type. We, note, of course that this simple approach has the substantial caveat that cell-type assignments are themselves extracted from the full gene-expression data and do not constitute independent experiments: for this reason, combining across cell-types, our results combining p-values should be understood as indicative.

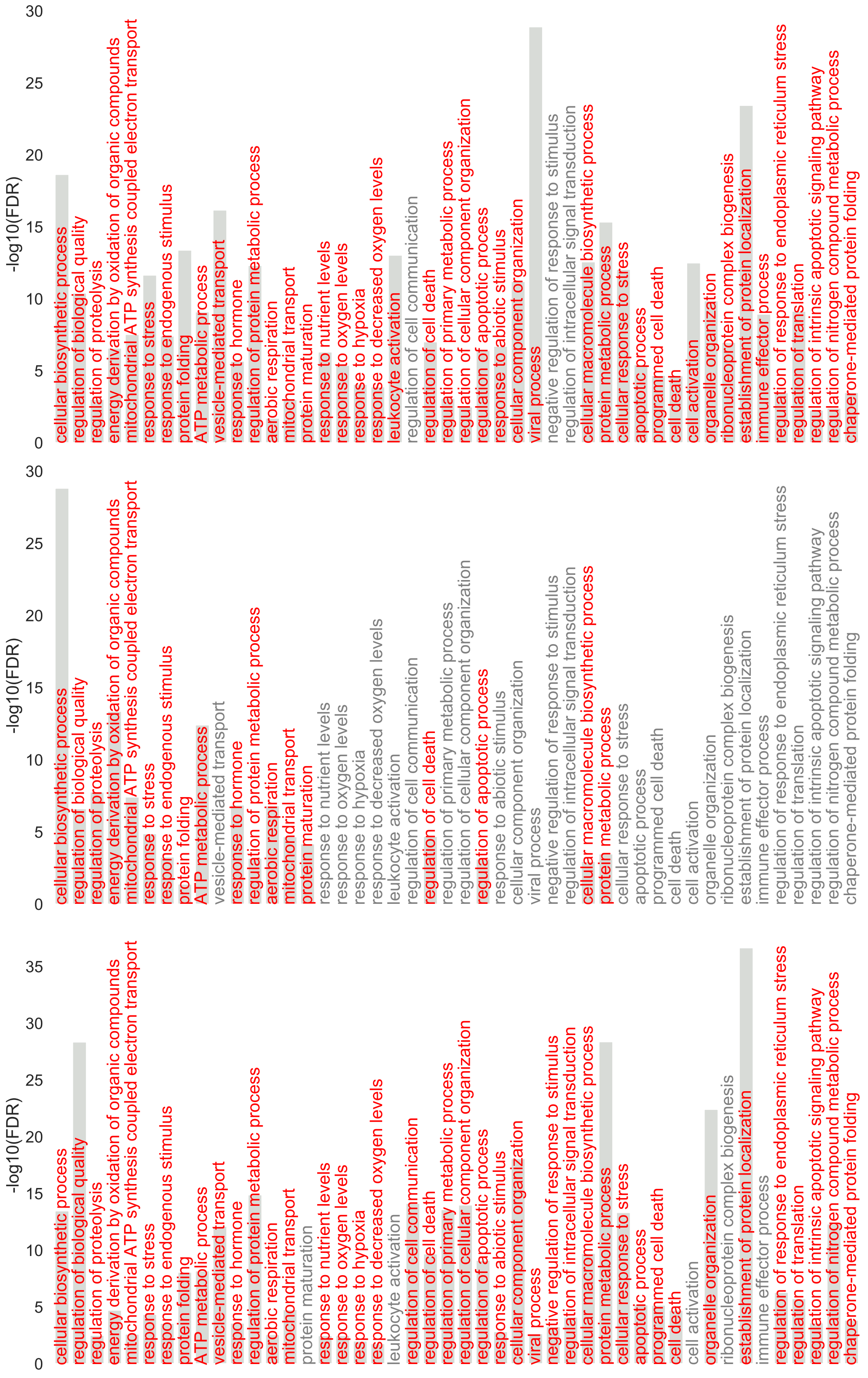

Supplementary Figure S14: **Transcriptional change correlated to the presence of cryptic mtDNA mutations is also present when making comparisons between cells of the same donor.** Human pancreas (Enge, top panel) data, the mice liver (middle panel), and the mice pancreas (bottom panel). We show the highlighted GO terms from the main manuscript and highlight them in red if significant with the Fisher's method.

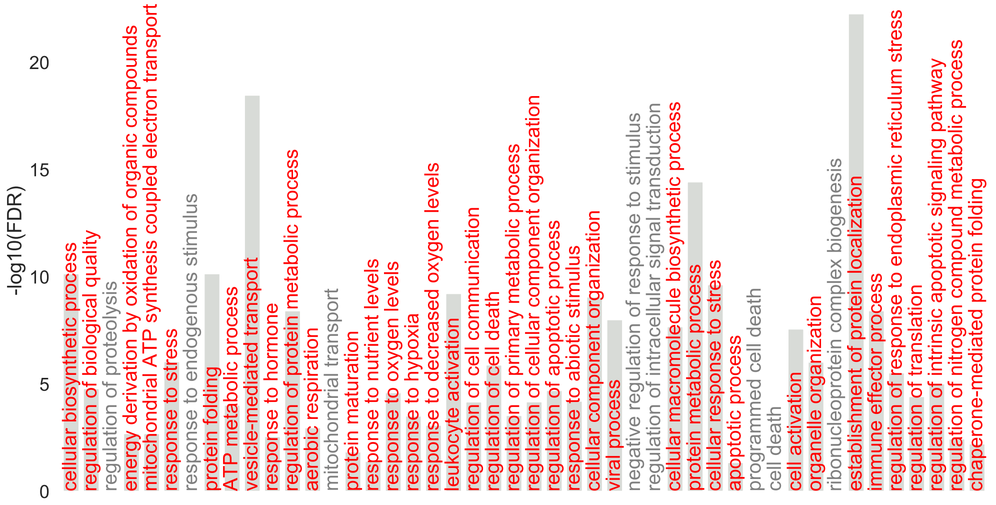

Supplementary Figure S15: **Restricting to a single cell type still allows the detection of transcriptional changes due to cryptic mtDNA mutations.** Transcriptional change correlated to the presence of cryptic mtDNA mutations is also present when making comparisons between cells of the same celltype for the Human pancreas data (Enge). We show the highlighted GO terms from the main manuscript and highlight them in red if significant with the Fisher's method.

S4 Supplementary Discussion: The role of the number of mitochondrial reads

Cells vary in their ratio Γ of the number of mitochondrial reads to the total number of reads, which could be a sign of varying mtDNA copying numbers. It is known that external factors, such as, oxygen tension may modulate the amount of mtDNA, as well as, heteroplasmy e.g. (*23*). It is also long known that mtDNA mutations can alter copy-number (*24*). The interplay between copy-number and mtDNA mutation is thus nuanced. For the human pancreas data (Enge *et al.*), we find that cells with $\mu > 0$ (i.e., cells with a cryptic mutation above 10 %) tend to have a slightly higher percentage of mitochondrial reads than cells without such a mutation ($\langle\Gamma\rangle_{\mu=0} \approx 11.07\,\%$ and $\langle\Gamma\rangle_{\mu>0} \approx 12.63\,\%$). Therefore, it is a hypothesis that the differences in gene expression that we identify is exclusively driven by the variability of the ratio Γ between these two groups.

To test this hypothesis, we use a sampling procedure that constructs a new data set with $\langle\Gamma\rangle_{\mu=0,\text{sampled}} \approx \langle\Gamma\rangle_{\mu>0} \approx 12.63\,\%$ (i.e. both sets of cells are controlled to have the same average number of reads) but otherwise the same characteristics (i.e., the same number of cells with and without cryptic mutations). To achieve this, we keep all mutated cells but sample with replacement from the cells without mutations such that $\langle\Gamma\rangle_{\mu=0,\text{sampled}} \approx \langle\Gamma\rangle_{\mu>0}$. Specifically, we construct a histogram with $n_{\text{bin}} = 8$ bins, depending on their Γ and then use an importance sampling procedure where the sampling weight for each bin is given by the ratio of the number of cells with $\mu > 0$ to the number of cells with $\mu = 0$ in this bin. This procedure yields a random sample with $\langle\Gamma\rangle_{\mu=0,\text{sampled}} \approx 12.61$. Repeating this procedure or increasing the number n_{bin} of bins yields similar results.

With this sampled data, we obtain a list of 1836 DEGs and the GO enrichment shows similar biological processes enriched as without the importance sampling procedure (see Fig. S16). This rejects the hypothesis the identification of DEGs is exclusively driven by a difference in the ratio Γ of the number of mitochondrial reads to the total number of reads.

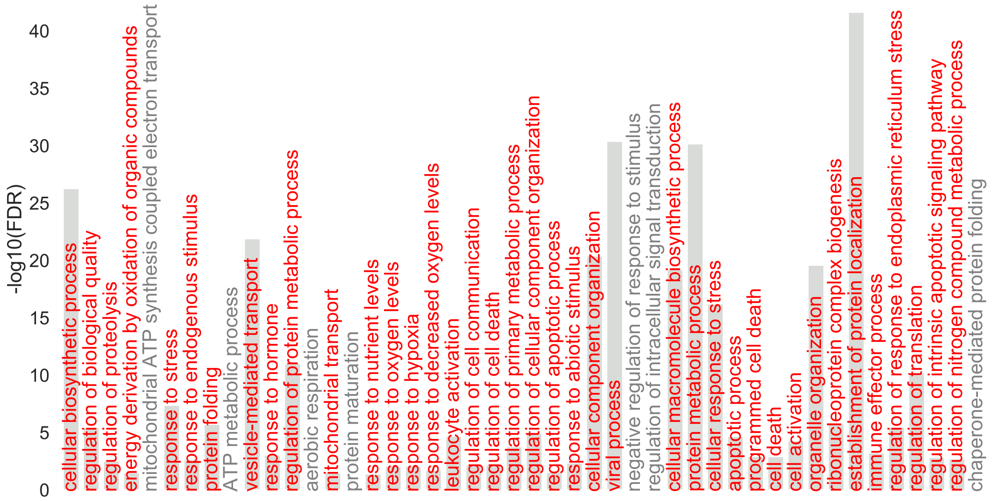

Supplementary Figure S16: **Correcting for mitochondrial coverage does not remove transcriptional changes due to cryptic mtDNA mutations.** Transcriptional change from presence of cryptic mtDNA mutations is also present after correcting for the ratio Γ of the number of mitochondrial reads to the total number of reads (human pancreas data, Enge). We show the highlighted GO terms from the main manuscript and highlight them in red if significant after the correction.

S5 Parkinson's disease and Alzheimer's disease single-nucleus RNA-seq data

In the main manuscript, we link cryptic mitochondrial load to gene expression in neuronal cells and in particular with genes linked to neurodegeneration. We showed this for a single-nucleus RNA-seq (snRNA-seq) data consisting of donors with Parkinson's disease (PD) and age-matched controls. Here, we investigate a second data set consisting of patients with Alzheimer's disease (AD) donors and age-matched controls (*25*). Given the small number of mitochondrial reads in snRNA-seq data (see Fig. S23), we call heteroplasmies at 10 reads and mark cells with a cryptic mutation which is not synonymous above 95 % as 'mutated'. Our aim is to identify whether the expression of genes is linked to the presence of cryptic mutations.

This data set is considerably smaller (3,759 cells after quality control, in comparison to 27,539 in the PD data) and therefore, we investigate only the top 6000 genes, to reduce the influence of the Benjamini–Hochberg procedure for multiple testing correction.

We find only MALAT1 to be differentially expressed after the multiple-testing correction. The functional role of the lncRNA MALAT1 was first identified in cancer (*26*) but has since then also been associated with neurodegenerative diseases (*27*). More generally, the identification of differentially expressed lncRNAs in the different data sets indicates that their

involvement in response to cryptic mutations might be similar to their dysregulation in human disorders (*28*).

Heat shock 70 kDa protein 1 (HSPA1A) and Glial fibrillary acidic protein (GFAP) are differentially expressed but not significant after the multiple testing correction. For both, however, there is external evidence that suggests a neurodegenerative function. The expression of GFAP in astrocytes is linked with old age and the onset of AD (*29*). Heat shock proteins interfere with apoptosis and these homeostatic functions are especially important in proteinopathic neurodegenerative diseases (*30*).

Overall, our results demonstrate that one can call high-heteroplasmy mutations from nuclei instead of whole cells. Despite the low coverage of mtDNA reads in snRNA-seq, we are able to identify cells with a high mitochondrial mutation load. We find evidence of a link between gene expression and cryptic mutations. In particular, the identified DEGs indicate that there is a change in inflammatory processes and lncRNAs which may be linked to neurodegeneration and senescence.

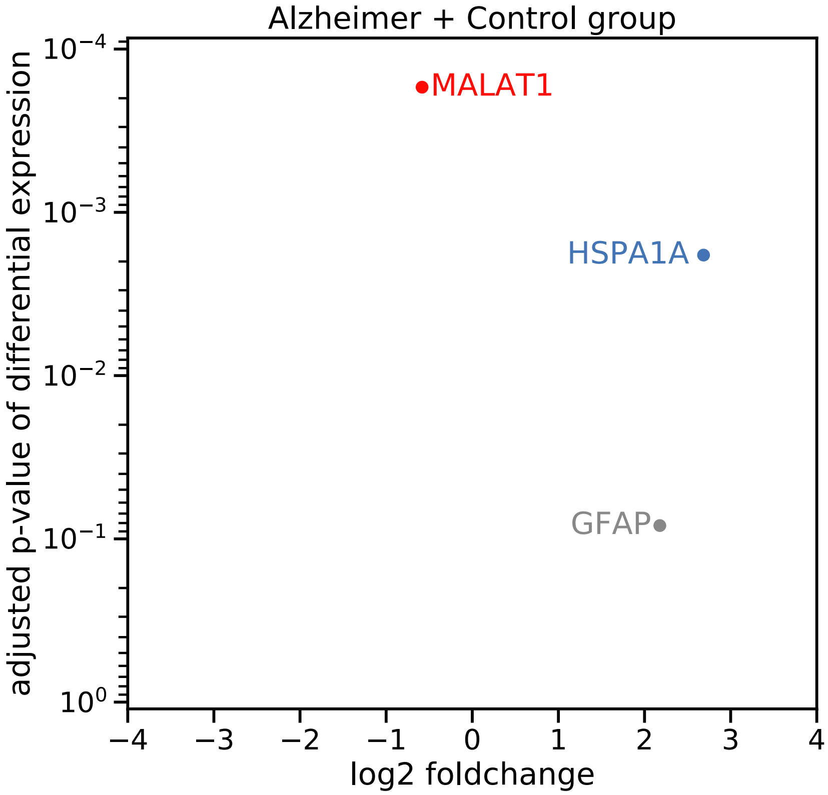

Supplementary Figure S17: **Differentially expressed genes in the Alzheimer's disease (AD) data.** We aggregate AD and Control group cells to identify genes that are perturbed in both groups. For greater statistical power, we only investigate the top 6000 most variable genes, which reduces the influence of the Benjamini–Hochberg procedure for multiple testing correction.

S6 Supplementary Discussion: Variant Calling from RNA

S6.1 Empirical corroboration

Throughout the manuscript, as well as using scATAC-seq, we analyse mitochondrial variants identified using scRNA-seq data, following similar efforts using scRNA-seq to do lineage tracing with mtDNA variants (e.g., (*31–33*)). We perform our own analysis of how accurately scRNA data reflects variants on the underlying DNA using paired scATAC and scRNA data from the 384 cells taken cultured from 4 different cell lines (*34*). Both scATAC-seq and scRNA-seq libraries were aligned using STAR (*35*) and then we identified variants using our custom variant calling script. The libraries were generated with a paired sequencing protocol which involved 7 more rounds of PCR that the standard smartseq2 protocol, and these rounds were held at higher temperatures for longer, which will results in more thermal errors (*36*) spreading to higher heteroplasmies than the other datasets considered in the main text. In addition to our usual quality control filters, we only take forward positions for variant analysis if they pass were covered by at least 200 reads in both the scATAC-seq and scRNA-seq libraries. After all quality control was applied there were 314 cells taken forward for analysis. Despite the quality of the dataset, we found that 92.2 % of variants with heteroplasmy above 10 % identified using scATAC-seq were also found using scRNA-seq, and there was a strong correlation of heteroplasmy between mutations identified by both techniques (Pearson test $r^2 = 0.76$).

In accord with Ludwig *et al.* (*31*) we also find many RNA specific mutations which likely come from RNA editing events or sequencing errors. 76.9 % of these mutations occur in more than one cell, likely reflecting common RNA mutations and so will be filtered out when we look at cryptic mutations. In this study of data from (*34*) we could (as we do in the maintext) improve the correlation between heteroplasmy called from RNA and DNA by using more stringent depth or heteroplasmy thresholds, but these trade off against the number of mutations detected reducing statistical power.

S6.2 Robustness to variant calling errors

Our study calls variants from scRNA-seq and snATAC-seq and these are termed cryptic mutations: its conclusions are robust to errors in calling any specific variant. We ensure robustness through comparative and aggregate analysis and careful selection of quality-control thresholds to eliminate noise sources. Our results are consistent with theory and are consistent across multiple tissues and species.

Aggregation and comparison: Our principal conclusions depend on comparisons of the cryptic site frequency spectrum (cSFS) and on creating populations of cells which are enriched in cryptic mutations. The process of calling variants, and assigning heteroplasmies, convolves a latent 'true' SFS distribution with an error kernel (existing mutations are assigned erroneous heteroplasmies through sampling effects and equivalents) and then adds a separate error distribution (non-existent mutations are erroneously identified). By making comparisons *between* cSFS distributions we control for common additive noise effects and our rational use of quality control (discussed below) and success at discriminating between ages of individuals suggests that the noise kernel has sufficiently bounded-variance. In a similar manner our differential expression analysis (comparing cells with and without cryptic mutations) does not require that we accurately identify cryptic mutations in every cell: only that we successfully create a set of cells which have an enriched rate of cryptic mutations.

Quality control: As noted by others there exist recurring RNA-mutations which may or may not be functional (*37*): we are able to eliminate this source of confounding from our study by restricting our study to cryptic mutations: those found in only one cell in the sample. As noted in our methods section these mutations might effect our analysis of selection effects in non-cryptic mutations (Fig. 2b) but we attempt to mitigate this by eliminating mutations that are found in 3 or more individuals. We also seek to eliminate noise from PCR and sequencing errors resulting in erroneous mutation calling, while maintaining the signal we wish to see. From our theory in S1 we find that the ageing effect of interest causes dynamics in the mid to high-heteroplasmy ranges of the SFS, and so we apply conservative filters to both the depth required for variant calling, and the heteroplasmy of variants we consider to eliminate noise which could obscure the signal of interest. To this end we consider sites with > 200 reads aligning and take forward variants with heteroplasmy $> 10\,\%$. In choosing these thresholds we not only focus on the physiologically relevant high-heteroplasmy range in the SFS, we also work to exclude PCR errors with the following argument. From the UMI data we have available, we find that the average number of reads per UMI is 7, and so, if we set the threshold for read depth as N, it represents on average ${}^{N}\!/_{7}$ true initial RNA molecules. Assuming a lower bound of PCR efficiency of 90 %, we calculate the maximum heteroplasmy a mutation occurring in the first round of PCR (and hence initial heteroplasmy $h_0 = \frac{7}{1.9N}$) will reach after 37 rounds of PCR to be 11.5 % when our read threshold is set at $N = 200$. Any positions with a higher read depth, or any mutation that occurs after the first round of PCR will have a lower maximum possible heteroplasmy, and so, by combining a heteroplasmy threshold of 10 % with minimum read-depth 200, we exclude the vast majority of PCR errors. We do not consider bases with a sequencing error probability of > 0.001 and so by choosing our thresholds to be this conservative there is a $< 10^{-15}$ probability of an erroneous heteroplasmy call from sequencing error.

Corroboration: We give a recapitulation of our main results using snATAC-seq in the next-but-one section. Our choices are also corroborated by the close accord between the our mathematical models of the extended site frequency spectrum (SFS) for a population of mtDNA and the cryptic site frequency spectrum (cSFS) we obtain. We find that young individuals have very few high frequency mutations in their cSFS (e.g. Fig. 1g and consistent with theory Fig. 1d) and that the cSFS also evolves with individual age in accord with theory (e.g. Fig 1h, Supplementary Material S1). Beyond finding results

from the cSFS consistent with theory, our consistent results across species add further support. While our results on the dynamics and effects of cryptic mutations would be equally interesting if they were driven by mutations that only occurred in the mtRNA (and not in the mtDNA) it is, however, challenging to provide a theoretical account for the dynamics of the cSFS if it is not being driven by mutations in the mtDNA.

S6.3 The effect of UMI collapsing on heteroplasmy calls

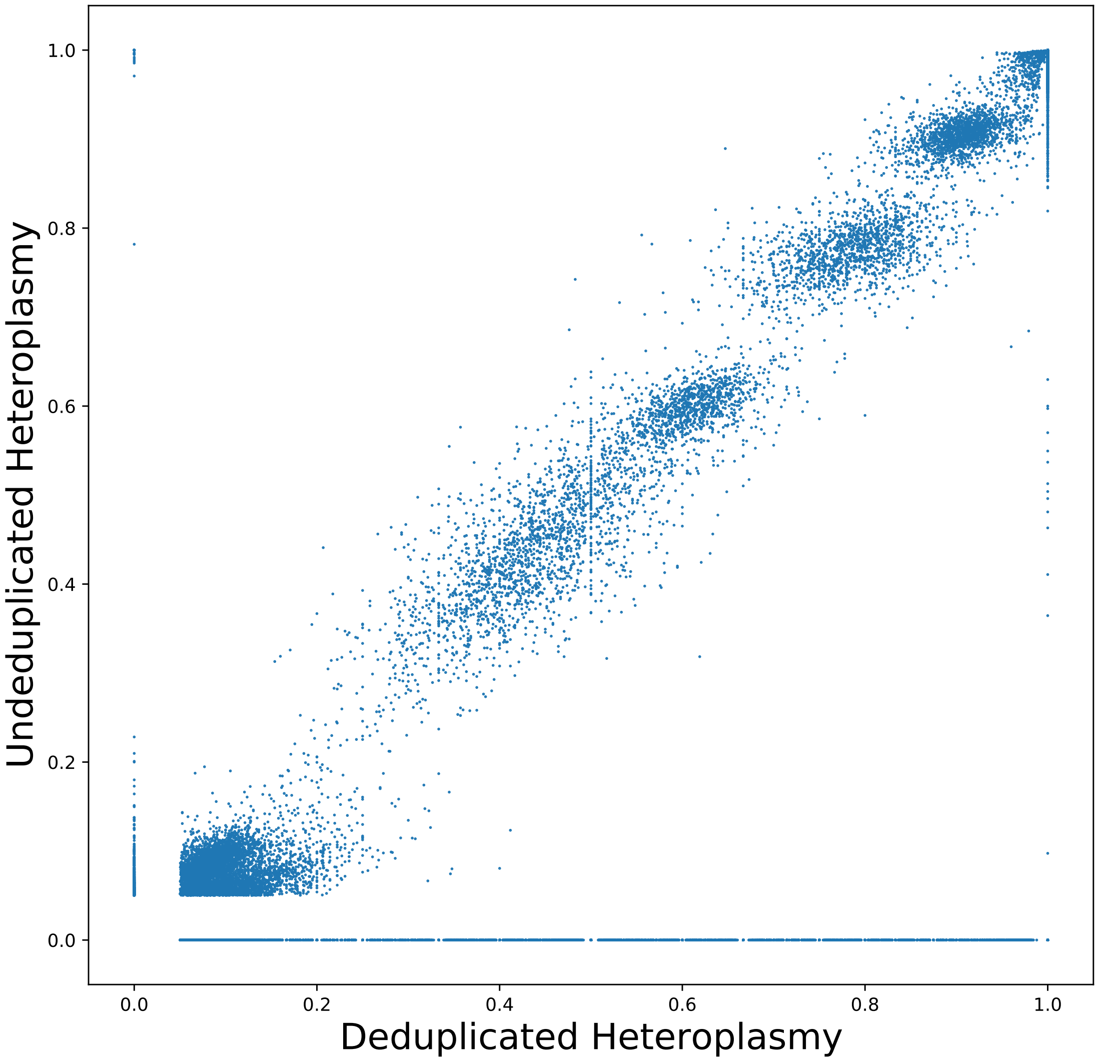

Supplementary Figure S18: **Heteroplasmy calling is in broad agreement both with and without UMI collapsing.** Here we called heteroplasmic variants using both raw reads and also by picking a representative read for each UMI in each cell with umi_tools dedup function (*38*). We called variants for the undeduplicated data at 200 read depth and deduplicated at 5 UMIs and estimated heteroplasmies for the Voigt data. This was done at a 5% heteroplasmy threshold. It can be seen that many variants are observed at the line y = 0 in the deduplicated data. These variants are likely attributable to the sequencing error caused by using so few UMIs and choosing a random representative. Therefore, deduplicated heteroplasmy calls at y=0 were excluded from regression analysis. We find excellent agreement between the heteroplasmies upon deduplicating and not deduplicating UMIs with $R^2 = 0.991$

S6.4 Mutations detected in snATAC data recapitulate results found from scRNA derived mutations

To further corroborate our findings from scRNA derived mutations, we reproduce key results of the main text using a 10x single-nucleus ATAC dataset (*18*) taken from various brain tissues of 6 cognitively healthy individuals with no neuropathological hallmarks of Alzheimer's. Though single-nucleus data attempts to exclude mitochondrial content, the process is not perfect and mitochondria which are associated with the peri-nuclear sheath can be captured (*39*, *40*). This results in much lower coverage of the mtDNA that our data used in the main text, and so we relax our depth threshold for variant calling to 10 reads, and raise our heteroplasmy threshold to 20 % to ensure at least two reads carry a mutation to bring forward in our analysis. With these depth and heteroplasmy thresholds, as well as our requirement for a site to be sequenced across multiple cell but only have a mutation in one, it is unlikely that any NUMTs sequenced will contribute to the cryptic mutations. We find that even with the extra noise introduce from snATAC, we replicate results regarding the evolution of the cSFS and selection of non-cryptic mutants from the main manuscript (Fig. 1f-h, 2b) as well as finding further evidence for tissue specific selection of non-synonymous cryptic mutations at heteroplasmies below $h < 20\,\%$ (Fig. 2d).

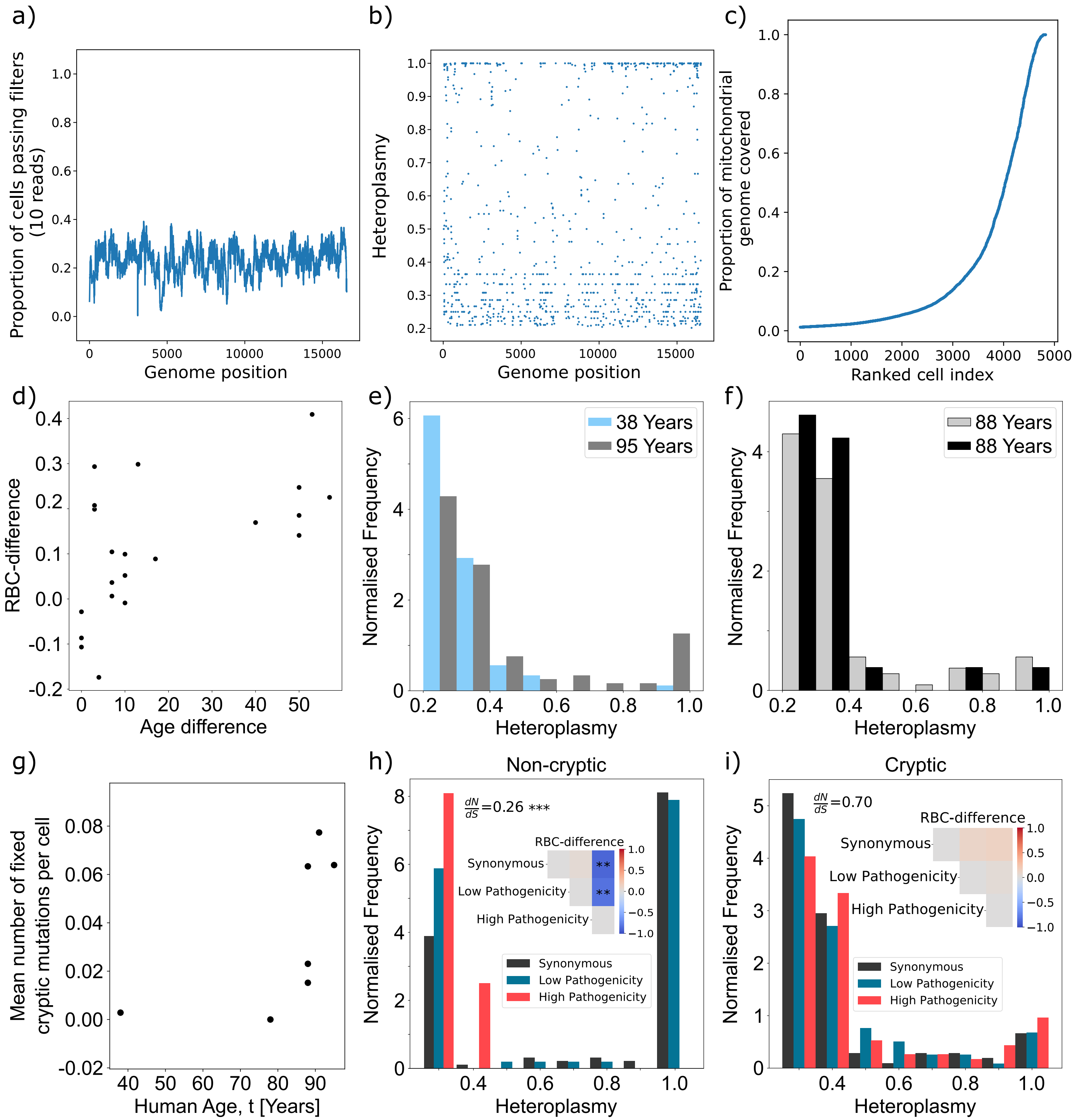

Supplementary Figure S19: **Results seen in the main manuscript are reproduced in a 10x single-nucleus ATAC dataset (*18*) derived from human brain tissue from 6 donors.** (a) At any specific position on the mitochondrial genome approximated 25 % of cells analysed pass our coverage criteria. (b) mutations are evenly distributed around the entire mitochondrial genome. (c) The proportion of the mitochondrial genome covered by each cell ordered by increasing coverage. (d) The RBC-difference (see Methods 2.6) between donors of different ages significantly increases with age (Pearson correlation $r \approx 0.56$ and $p < 0.01$). (e-f) We present two pairs of cSFSs taken from donors of differing ages (e) and donors of the same age (f) to see that, as in the cSFSs shown in the main manuscript, older individuals have a cSFS shifted to higher heteroplasmies than younger individuals. (g) There is a significant increase of cryptic homoplasmic (heteroplasmy > 0.9) mutations with age (Spearman correlation $r \approx 0.89$ and $p < 0.01$). (h) We also look at selection effects first on all non-cryptic mutations and find a significant shift in the non-synonymous/synonymous ratio below 1 (Fisher's exact test $p < 10^{-8}$) as well as in the SFS of both high and low pathogenicity mutations when compared to synonymous mutations ($p < 10^{-4}, 0.001$ respectively) (i) Cryptic mutations have a significant shift below 1 of their non-synonymous/synonymous ratio (Fisher's exact test $p < 0.05$), but as seen in mouse data (Fig. 2d) there is no evidence of a shift in the cSFS hinting at selection occurring on mutations with low heteroplasmy ($< 20\,\%$). Cell and mutation counts for all SFSs are found in table S5.

S7 Supplementary Tables

Figure	SFS label	Cells	Mutations
Fig. 1f	1 Month	93	206
Fig. 1f	54 Years	129	211
Fig. 2a	Synonymous	201	229
Fig. 2a	Low Pathogenicity	312	375
Fig. 2a	High Pathogenicity	290	337
Fig. 2b	Synonymous	2180	92
Fig. 2b	Low Pathogenicity	2166	48
Fig. 2b	High Pathogenicity	160	29
Fig. 2c	Synonymous	836	57
Fig. 2c	Low Pathogenicity	839	42
Fig. 2c	High Pathogenicity	478	19
Fig. 2d	Synonymous	585	709
Fig. 2d	Non-synonymous	1132	1797
Fig. 4a	Young Ad Libitum	50	50
Fig. 4a	Old Caloric Restricted	96	100
Fig. 4a	Old Ad Libitum	67	68
Fig. 4b	Young Ad Libitum	125	133
Fig. 4b	Old Caloric Restricted	145	154
Fig. 4b	Old Ad Libitum	58	58
Fig. S19e	38 Years	471	3862
Fig. S19e	95 Years	373	2985
Fig. S19f	88 Years (grey)	395	2674
Fig. S19f	88 Years (black)	125	706
Fig. S19h	Synonymous	1808	194
Fig. S19h	Low Pathogenicity	1493	180
Fig. S19h	High Pathogenicity	144	163
Fig. S19i	Synonymous	706	838
Fig. S19i	Low Pathogenicity	799	967
Fig. S19i	High Pathogenicity	724	849

Table S5: For every displayed normalised site frequency spectrum we give the total number of cells that the mutations were found in as well as the total number of mutations which make up the SFS (for non cryptic SFSs we count each unique mutation once per individual and take the average heteroplasmy it is found at).

Dataset	Figure	Cells carrying cryptic mutation	Cells without mutation
Enge (*14*)	Fig. 3a, c	774	1577
Muraro (*20*)	Fig. 3c	123	2302
Voigt (*19*)	Fig. 3c	236	5305
Tabula Muris Liver (*21*)	Fig. 3c	1022	500
Tabula Muris Pancreas (*21*)	Fig. 3c	541	1571
Ma BAT (*41*)	Fig. 3c	265	28900
Ma Liver (*41*)	Fig. 3c	180	20093
Samjic (*16*)	Fig. 4k	119	3640

Table S6: For each of the differential gene expression analyses presented in the main text we give the number of cells in each of the two classes

S8 Supplementary Figures

S8.1 Differing sequencing techniques have varying coverage of the mitochondrial genome resulting in fewer possible variant locations

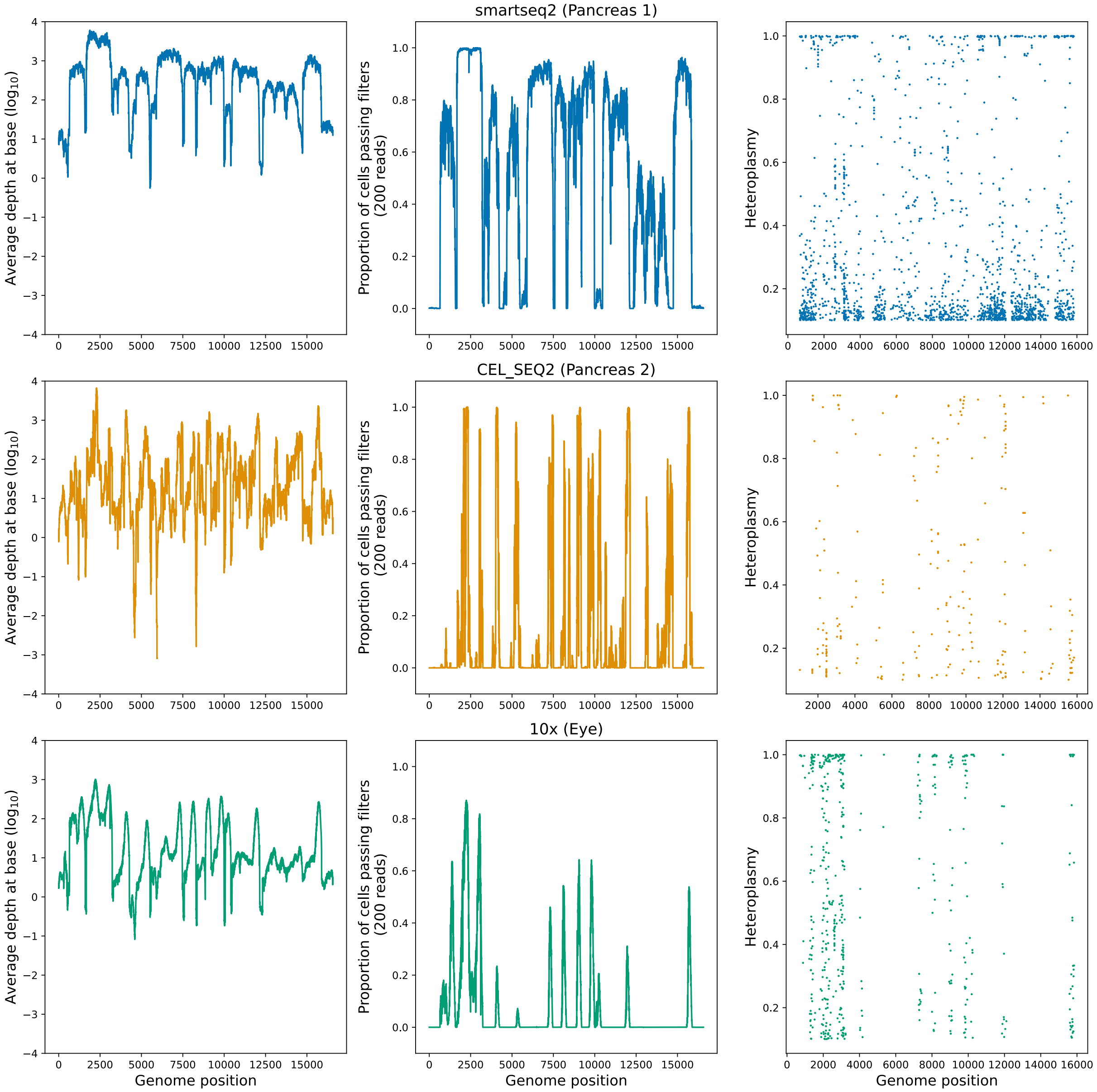

Supplementary Figure S20: **Comparison of coverage for different sequencing techniques.** Throughout the paper we use data from three different scRNA sequencing technique, both full length and 3'. We show how the coverage of the mitochondrial genome differs for each technique, and how this affects the number of potential mutations we can identify. As expected full length sequencing techniques provide the most coverage across the mitochondrial genome, but for all techniques we observe that mutations are spread evenly across the sequenced regions. We find that due to the 3' nature of the two lower coverage sequencing techniques (CELSEQ2 and 10x) their coverage overlaps consistently with at least 78 % of bases in our lowest coverage dataset being covered by the other technologies.

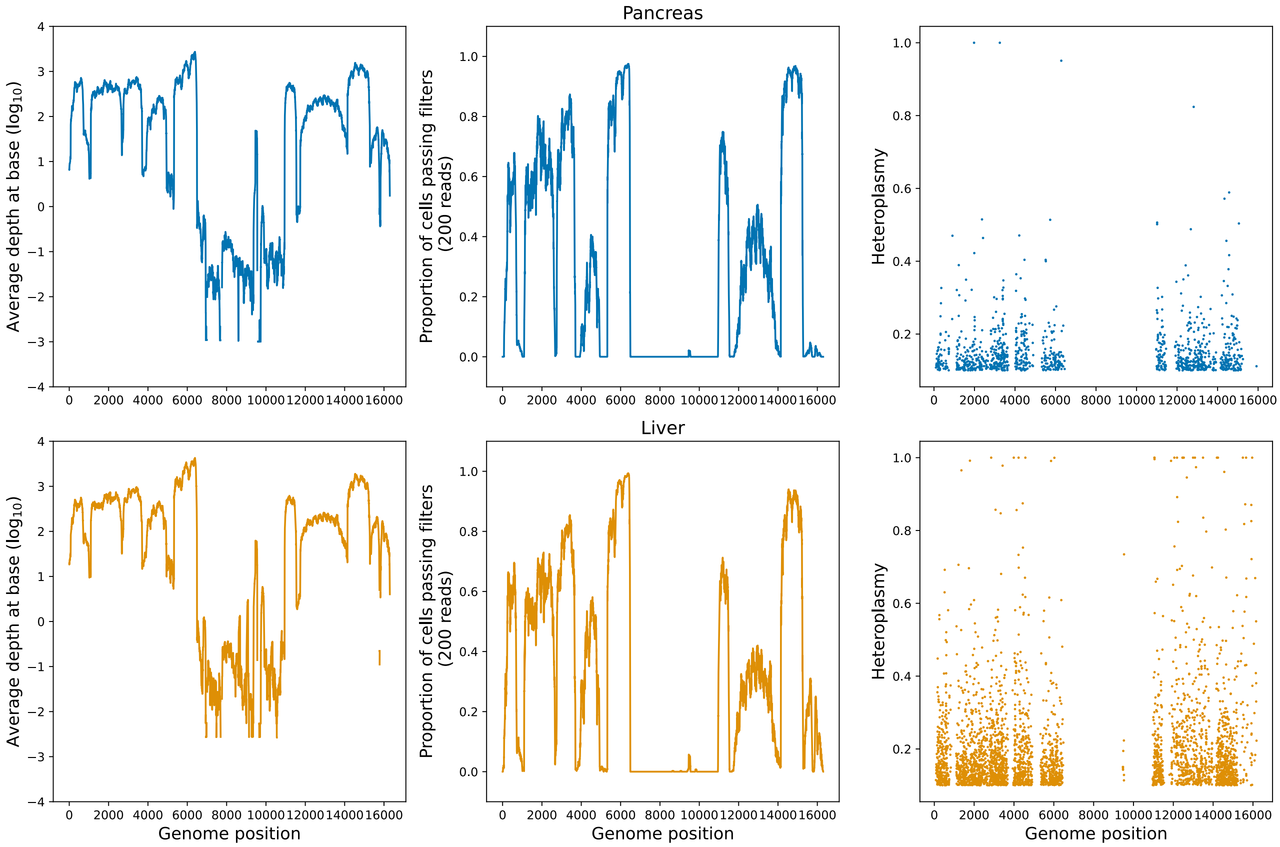

Supplementary Figure S21: **We show how the coverage of the Tabula Muris Senis dataset (*21*) differs to that of human datasets.** Notably there is a drop in coverage between 6500-11000bp of the mtDNA of mice due to a large NUMT in the reference genome and our exclusion of multimapped reads

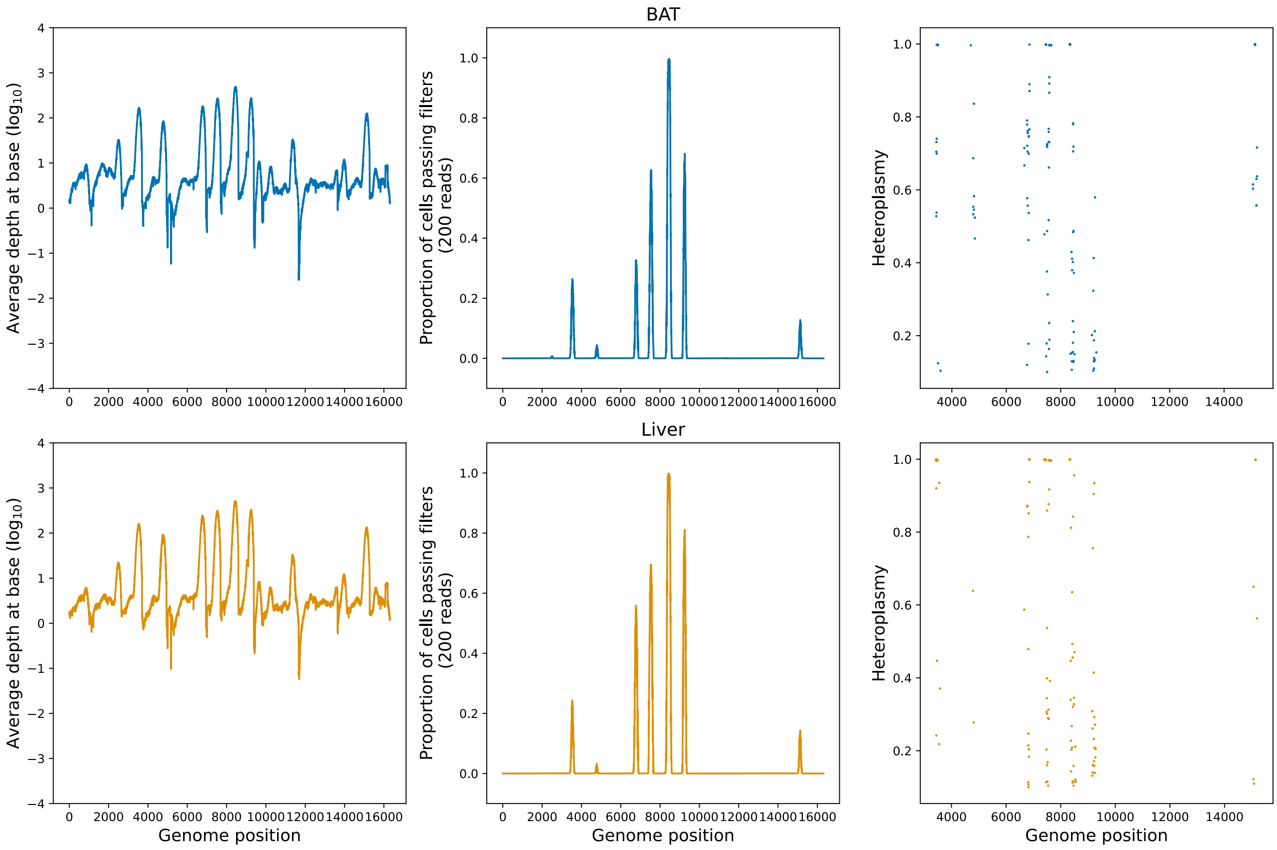

Supplementary Figure S22: **We also show the coverage of the rat caloric restriction dataset (*41*).** We find that the coverage of this dataset is an order of magnitude less than the equivalent 10x dataset in humans. In order to investigate caloric restriction, this dataset had variants called at a 5% heteroplasmy threshold, enabling greater quantities of variants to be detected in the dataset. Variant calling on both liver and brown adipose tissue at the 5% and 10% threshold had the mean heteroplasmy of mutations in young ad libitum and old calorically restricted mutations within 3% of each other, whilst the old ad libititum always had a mean heteroplasmy $>$ 5% larger than both young ad libitum or old calorically restricted. Combining p-values from Mann-Whitney U tests between groups for both tissues for each difference separately using Fisher's method gives the following p-values 0.381, 0.215, 0.131, for the parenthesised comparison pairs (young ad libitum, old calorie restricted), (young ad libitum, old ad libitum), (old calorie restricted, old ad libitum) respectively.

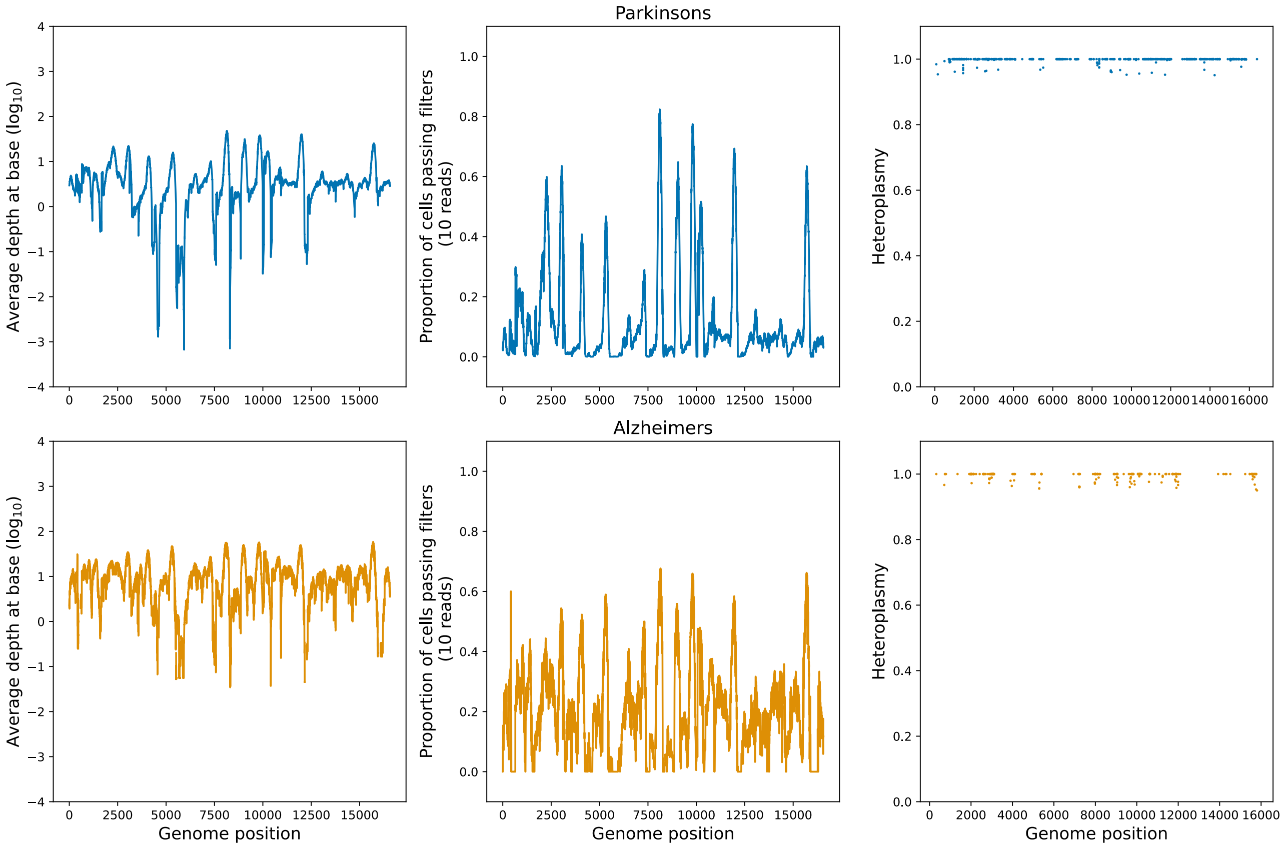

Supplementary Figure S23: **The coverage from two single-nucleus datasets (*16*, *25*) is consistently lower than that of single-cell experiments.** To be able to use this data we relax our read depth threshold to 10 reads, and only call variants if their heteroplasmy $h > 95\,\%$

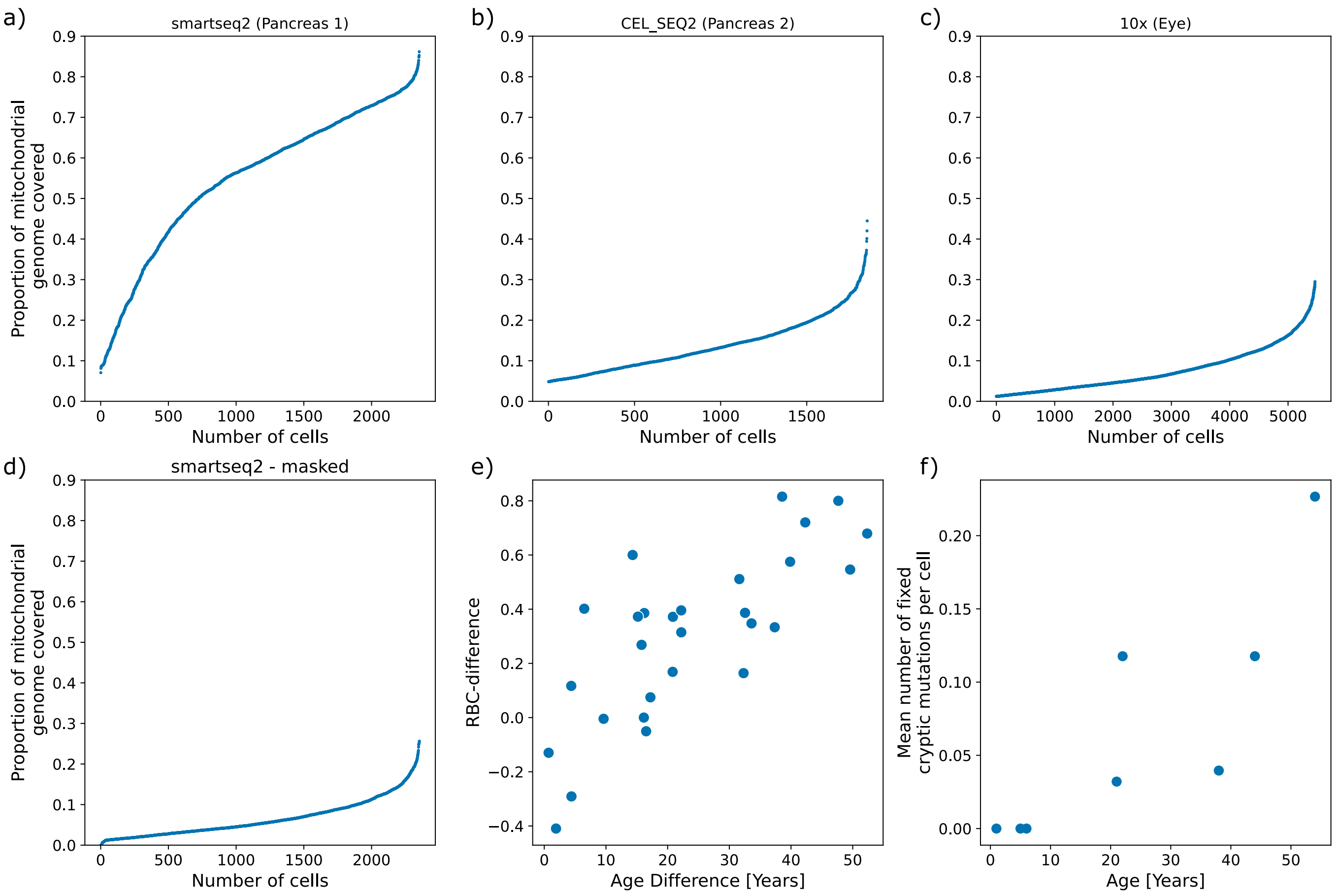

Supplementary Figure S24: **The heterogeneous coverage of different sequencing technologies does not affect our conclusions.** (a-c) The proportion of the mitochondrial genome covered by each cell, ordered by increasing coverage, for the three main human datasets. (d) We subset the bases observed in our dataset with the highest coverage by pairing cells at random from the 10x dataset with the smartseq2 and masking all bases which did not meet the filtering criteria in both of the paired cells. (e) With this new masked list of variants we examined the RBC difference between donors and found that the RBC-difference between donors increases with age difference (Spearman correlation $r \approx 0.69$ and $p < 0.001$). (f) We also found that this masked list of variants still showed an increase of homoplasmic mutations with age (Spearman correlation $r \approx 0.95$ and $p < 0.001$)

S8.2 Increasing age difference evolves the cSFS further apart

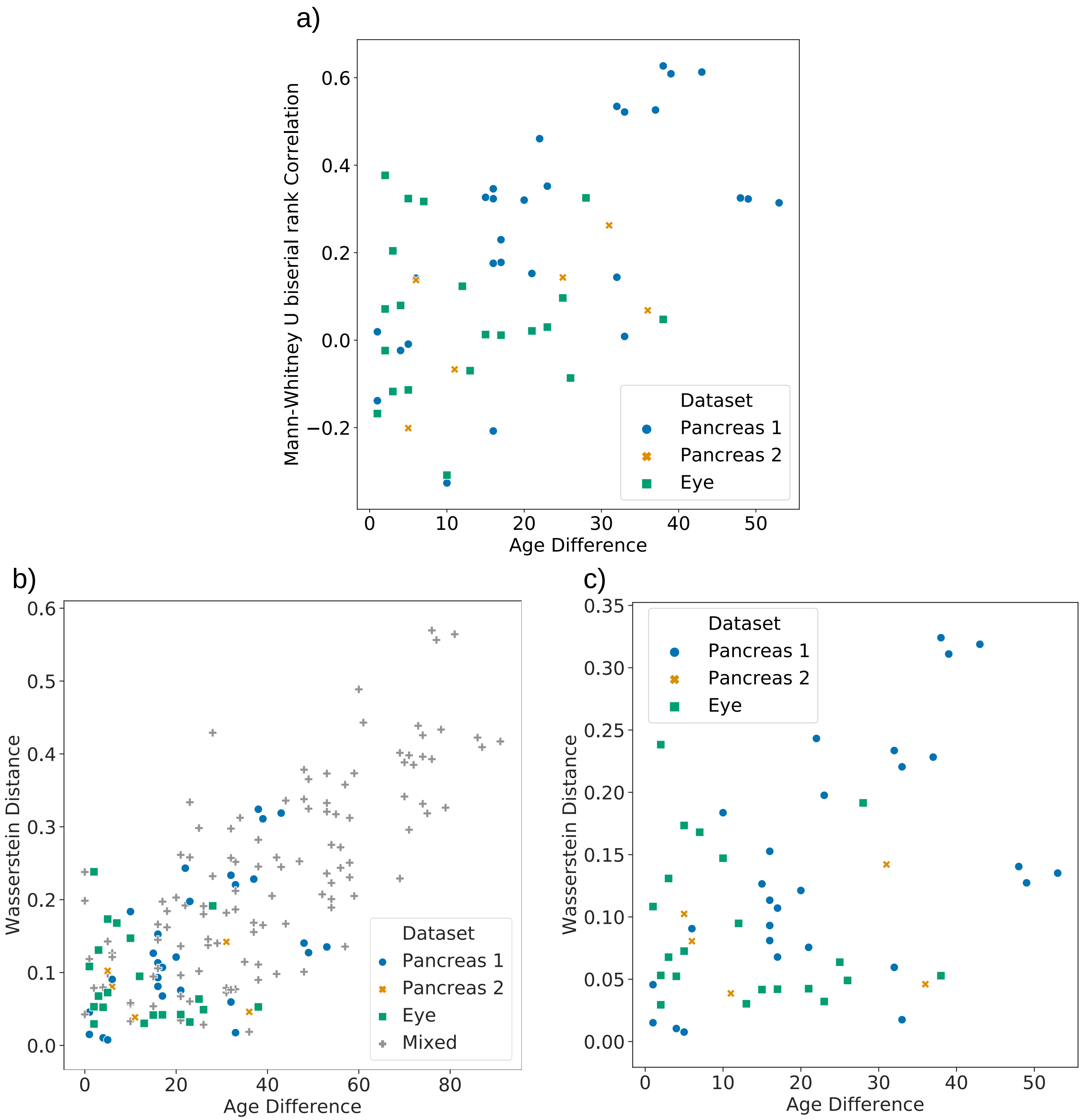

Supplementary Figure S25: **Using other metrics of distance between cSFSs we still find a significant correlation between distance between spectra and difference in age.** (a) When no comparisons between donors from different datasets is done, we still see a significant increase in the rank biserial correlation difference (RBC-difference, see methods 2.6) with difference in donor age (Spearman correlation $r \approx 0.52$ and $p < 10^{4}$). (b) We can compare the cSFS of donors using the Wasserstein distance as an alternative measure of distance and find that the Wasserstein distance also significantly increases with age (Spearman correlation $r \approx 0.70$ and $p < 10^{-25}$). (c) This significant increase also holds if we only consider comparisons of donors taken from the same datasets (Spearman correlation $r \approx 0.33$ and $p < 0.05$)

S8.3 Restricting to a single cell type leaves results unchanged

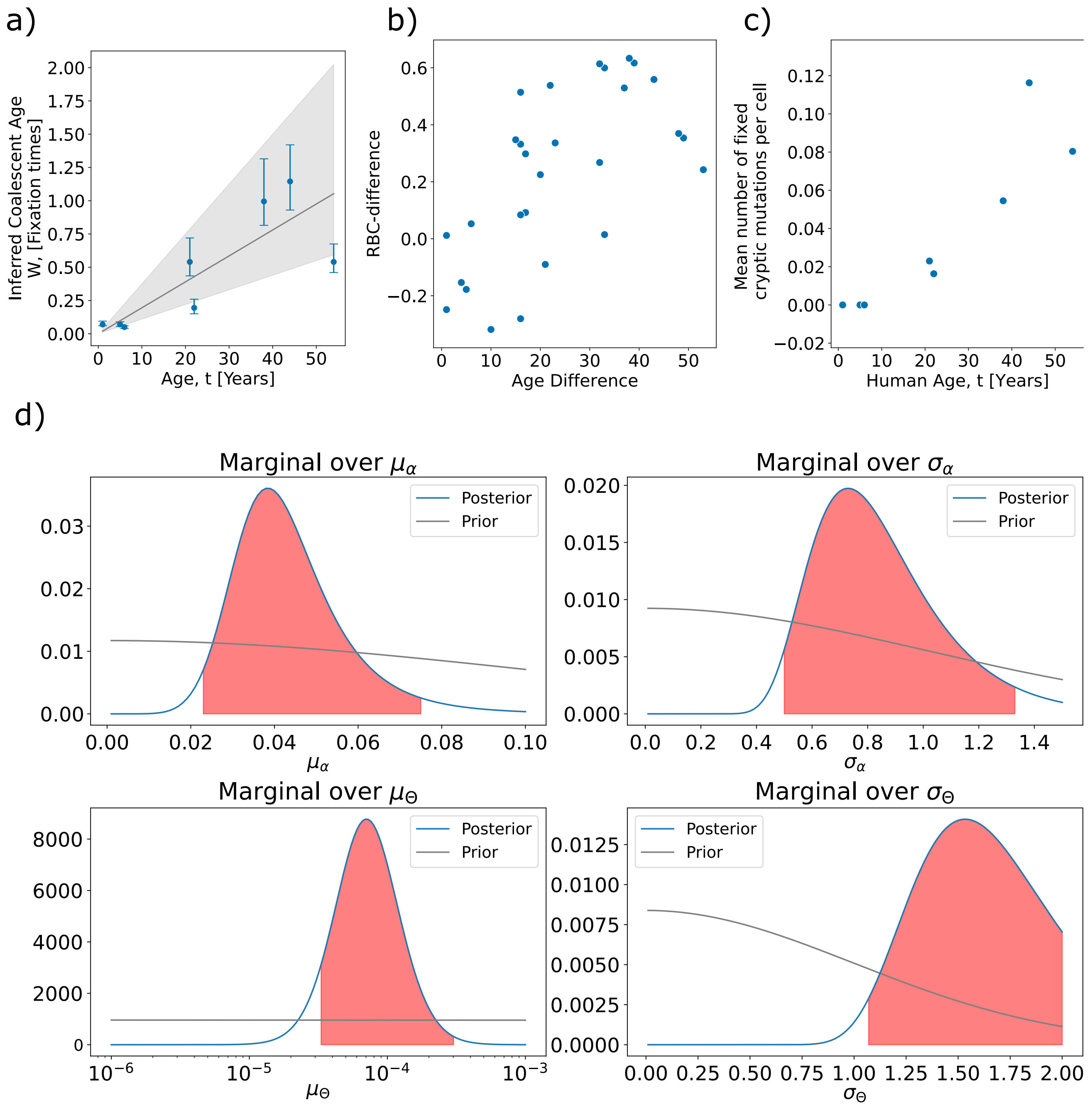

Supplementary Figure S26: **We restrict ourselves to only using the most abundant cell type of the Enge pancreas dataset (*14*) (alpha cells) and find results are unchanged.** (a) The inferred coalescent age using only alpha cells with the best fit and 95 % credible interval for the regression. (b) The RBC-difference between cSFSs increases with the age difference between the donors (Spearman Correlation $r \approx 0.64$ and $p < 10^{-3}$). (c) The number of homoplasmies increase with age (Spearman Correlation $r \approx 0.91$ and $p < 10^{-2}$). (d) The inferred posteriors of the hyperparameters are wider due to the reduced amount of data, and the inferred population mutation rate is higher, driven by the youngest donors.

S8.4 Mitochondrial ageing has multiple eras corresponding to mutations accumulating at different heteroplasmy levels

In the main manuscript we show that the model predicts an early life equilibration of low heteroplasmy mutations, a mid life accumulation of mid-high heteroplasmy mutations and a late life accumulation of homoplasmic mitochondrial mutations. Shown in figure S27 are these potential "mitochondrial eras", with all fits done using the MAP estimates from our Bayesian model (see Supplementary Discussion S2). The black line shows the Kullback–Leibler divergence of the out-of-equilibrium cSFS of heteroplasmies from its in equilibrium counterpart. Its fast decrease indicates that the majority of the cSFS, which is found at low heteroplasmies, equilibrates very quickly. The red line shows the relative probability of a heteroplasmic mutation being found at heteroplasmy $> 60\,\%$ (excluding homoplasmy) when the out-of-equilibrium SFS is compared to the equilibrium SFS ($60\,\%$ being an indicative heteroplasmy at which cellular dysfunction occurs). By 25 years old the relative probability is 0.25, increasing to 0.75 by 55 years. The blue line shows the mean number of homoplasmic mutations per cell, which undergoes non-linear dynamics up to around 55 years old, and then becomes a linear accumulation of mutations dependent only on the mutation and turnover rate of mtDNA. These eras align with early, mid and late life in humans.

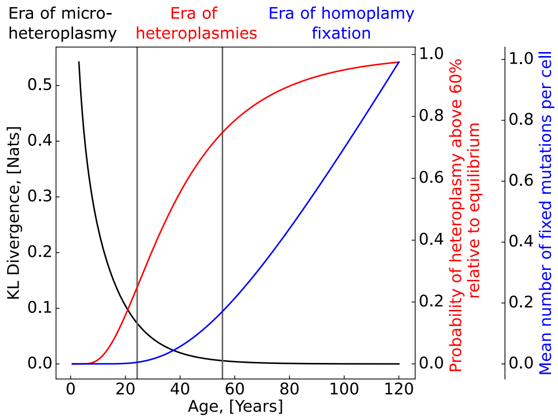

Era	Age range	Explanation
Era of microheteroplasmy	0–25	Fast equilibrium of low heteroplasmy mutations with little cell-to-cell variation of mtDNA mutant load. Era length (for reasonable mutation rates) dependent on population size and turnover rate.
Era of heteroplasmies	25–55	Longer timescale equilibriation of macroscopic heteroplasmies with marked cell-to-cell variation of mtDNA mutant load. Era length (for reasonable mutation rates) dependent on population size and turnover rate.
Era of homoplasmy fixation	55–	Continuous accumulation of homoplasmic mutations from the equilibrium heteroplasmic site frequency spectrum. Cell-to-cell variation of mtDNA mutant load converges as all cells accumulate an increasing number of homoplasmic mutations. Era length (for reasonable mutation rates) dependent on mutation rate and turnover rate.

Supplementary Figure S27: **Three eras of mtDNA mutation accumulation.** Table gives purely indicative ages. Notably it is only the very long term accumulation of homoplasmies, in late life, which resembles a linear accumulation of mutations. Turnover rate and cellular mtDNA population size define the equilibration phase (era of micro and macro heteroplasmies) whereas (for long times) mutation rate and turnover rates define the rate of accumulation of homoplasmic mutations (in manner independent of cellular mtDNA population size)

S8.5 Correlation between donor age and mitochondrial load as a function of the heteroplasmy thresholds

In the main manuscript, we demonstrated that the cellular mitochondrial load $\mu^{10\,\%}$ of cryptic, not synonymous mutations above a heteroplasmy of $10\,\%$ is positively correlated with the age t of its donor. This indicates that cells accumulate high-heteroplasmy cryptic mutations throughout ageing in accord with our theory (see SI-section S1). To test whether this result depends on the heteroplasmy threshold $h \in [0,1]$, we compute the Spearman correlation $Corr(\mu^{10\,\%}, t)$ for various heteroplasmy thresholds (see Fig. S28). We find that for all heteroplasmy thresholds $h > 10\,\%$, the correlation is significant at a threshold of $\sigma = 0.05$. Thus, we choose $h = 10\,\%$ as default heteroplasmy threshold to keep the most data. In Fig. S28, We observe that the p-value of correlation decreases for low thresholds because low-heteroplasmy mutations are present across all ages. For larger thresholds we filter out biologically significant mutations and therefore the correlation decreases, leading to an increase in the p-value with t. For $h \in \{10\,\%, 30\,\%, 40\,\%, 95\,\%\}$, we show the scatters for $(\mu^{h\,\%}, t)$ in Fig. S29. The correlation is still significant for $h = 95\,\%$, which is in accordance with the positive correlation between the mean number of fixed cryptic mutations per cell and the age of the donor (see Fig. 1i in main text).

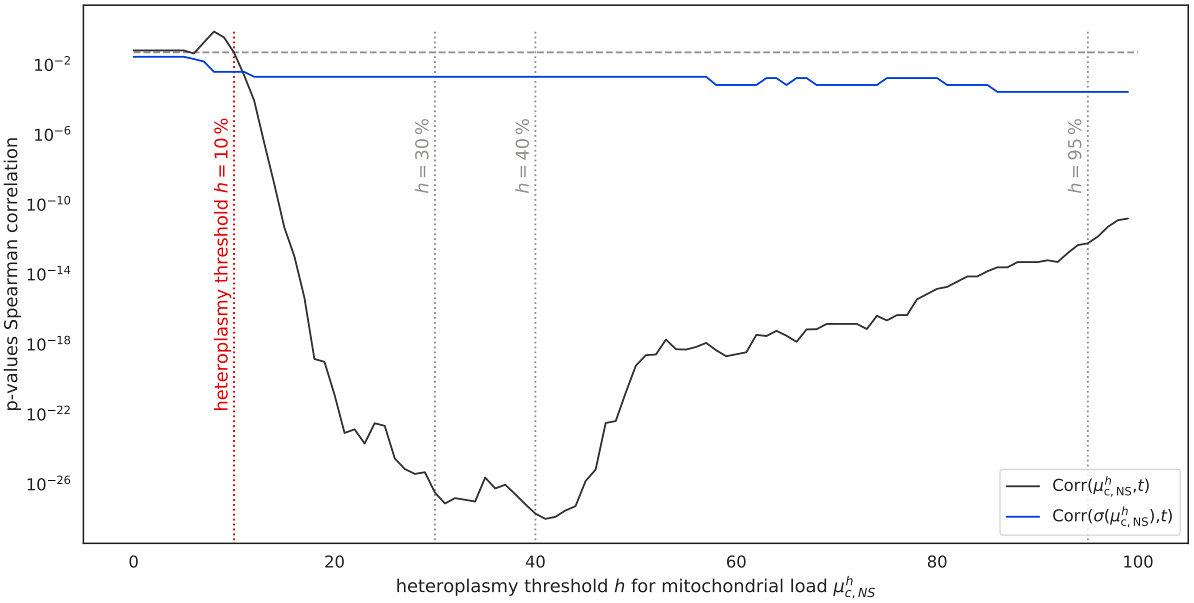

Supplementary Figure S28: **The correlation between mitochondrial load $\mu^{h\,\%}$ and age t is statistically significant for a wide range of heteroplasmy thresholds h for the full-length RNA-seq human pancreas data (Enge (*14*)).** This also holds for the correlation between the standard deviation $\sigma(\mu^{h\,\%})$ of the mitochondrial load and age t. The dashed horizontal line indicates a significance threshold of 0.05. We observe that the p-value of the correlation decreases for low thresholds because low-heteroplamsy mutations are present across all ages. For larger thresholds we filter out biologically significant mutations and therefore the correlation decreases. The correlations are still significant for $h = 1$ because the homoplasmic mutations increase with age, as show in Fig. 1i in the main text.

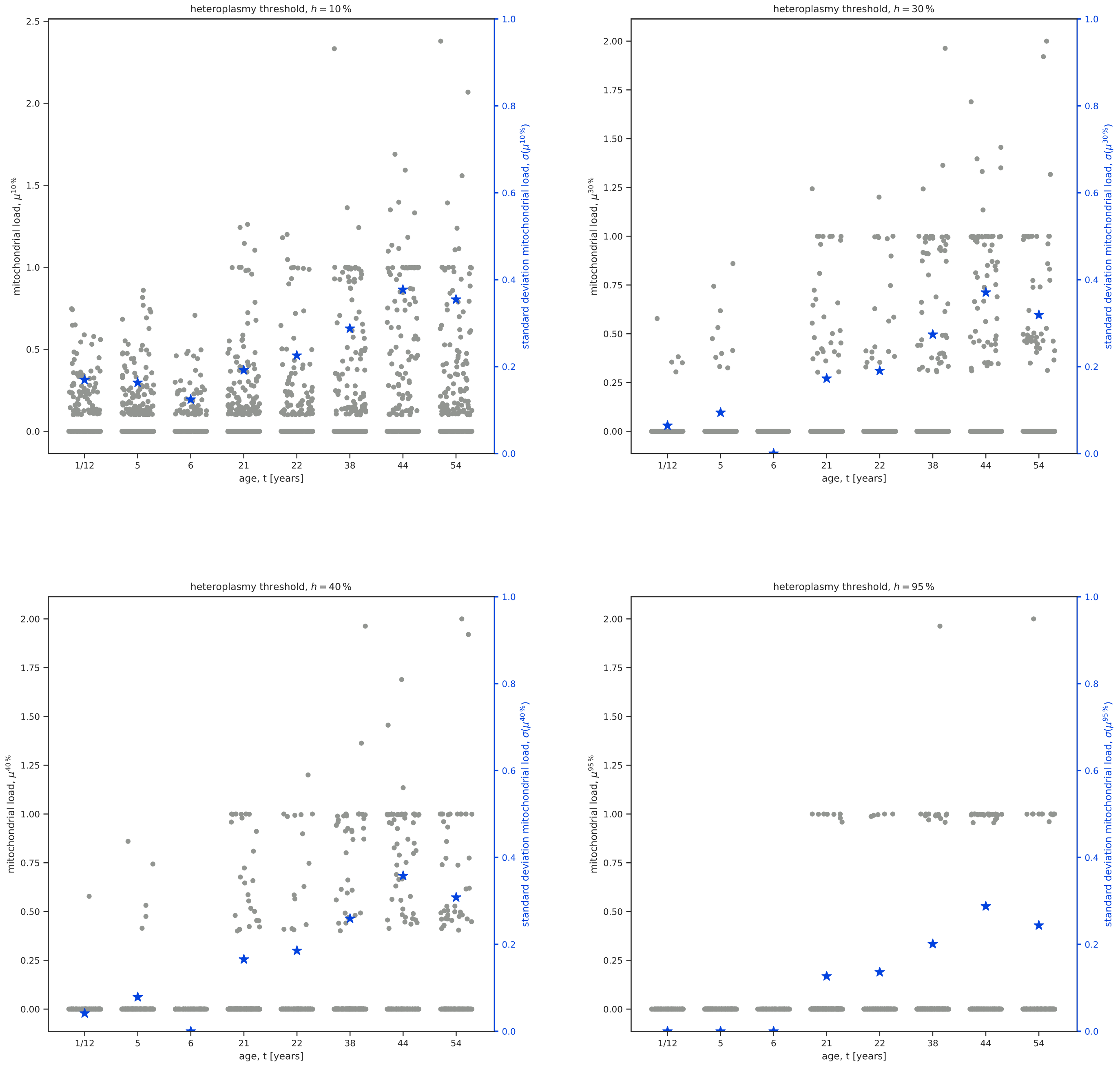

Supplementary Figure S29: **Mitochondrial load is correlated with age regardless of heteroplasmy threshold used.** Scatterplots of mitochondrial load $\mu^{h\,\%}$ versus donor age t for all cells in the full-length RNA-seq human pancreas data (Enge (*14*)). We give results for four heteroplasmy thresholds $h \in \{10\,\%, 30\,\%, 40\,\%, 95\,\%\}$ of which we use $h = 10\,\%$ in the main manuscript.

S8.6 Mammalian lung single-cell RNA data

In the main manuscript we show that homoplasmic mutations accumulate over an organisms lifespan. We look across 4 mammalian species (*42*) sequenced using 10x scRNA-seq and find that young pigs (15 weeks old), young mice (9 weeks old), and young rats (9 weeks old) have no homoplasmic mutations, whereas aged humans (76 and 88 years old) carried 0.14 cryptic homoplasmic mutations per cell. Due to the low depth of 10x scRNA-seq data we identify mutations at sites covered with a depth greater than 10 reads with a heteroplasmy $> 95\,\%$. We can relax this heteroplasmy threshold to examine the entire cSFS of these species and we find that not only do the humans carry more homoplasmic mutations, they also carry more mutations with heteroplasmies $> 10\,\%$ S30.

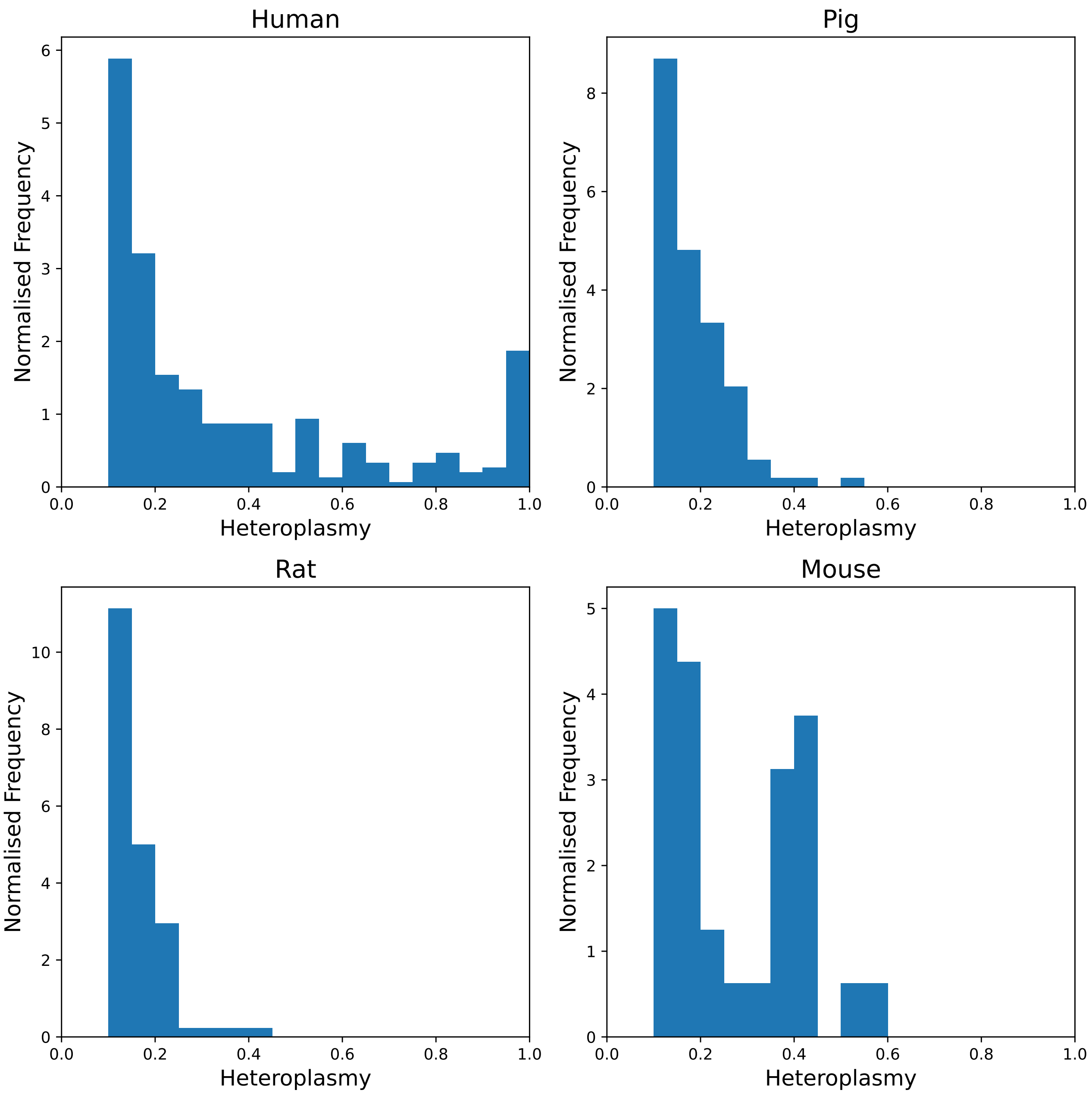

Supplementary Figure S30: **We examined scRNA data from multiple mammalian lungs to identify if species with different lifespans accumulate mutations at vastly different rate.** We found that aged humans (76 and 88 years old) have more cryptic mutations at both the homoplasmic (heteroplasmy $> 95\,\%$) and the heteroplasmic (heteroplasmy $> 10\,\%$) levels than young pigs (15 weeks old), mice (9 weeks old), and rats (9 weeks old). Performing pairwise Fisher's exact test for each species pair between the number of mutated and unmutated sites observed above the heteroplasmy thresholds, we found for against mice, rats and pigs, there were significantly more homoplasmic mutations in humans ($p < 0.005, 10^{-6}, 10^{-5}$ respectively). We found the same results when considering all cryptic mutations with heteroplasmy $> 10\,\%$ ($p < 0.05, 10^{-9}, 0.005$ against mice, rats and pigs respectively)

S8.7 Volcano plots for full-length human pancreas data at different heteroplasmy thresholds h

In the main manuscript, we show and discuss the differentially expressed genes (DEGs) in the full-length human pancreas data (*14*) at a heteroplasmy threshold $h = 10\,\%$. Here, we show DEGs for four heteroplasmy thresholds $h \in \{10\,\%, 30\,\%, 40\,\%, 95\,\%\}$ (see Fig. S31).

For all these heteroplasmy thresholds, we observe a large number of DEGs. Notably, see that different genes are covary with the presence of mtDNA mutations at different heteroplasmy thresholds h. This is in accord with the well-established notion of mitochondrial threshold effects, which mean that genetic effects manifest often only if they are present in sufficiently many mtDNA molecules (*43*, *44*). Nevertheless, we also find a substantial number of genes deferentially expressed across thresholds.

Surprisingly, we find that despite the small number of (almost) homoplasmic mtDNA mutations, we identify DEGs at a heteroplamsy threshold of 95 %. This indicates, that these mutations alter the gene expression substantially.

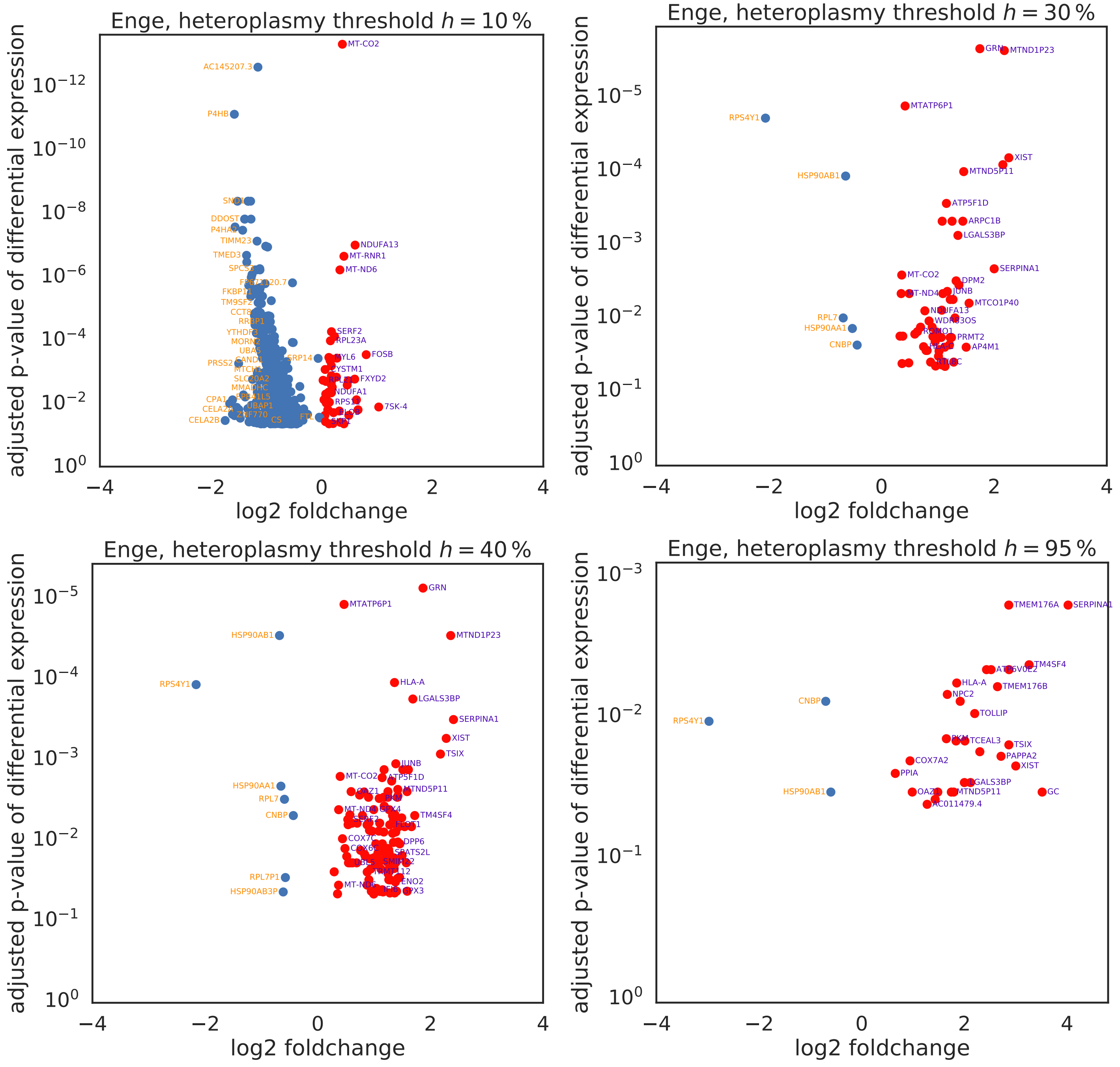

Supplementary Figure S31: **Differentially expressed genes are detected regardless of heteroplasmy threshold used.** Differentially expressed genes in the full-length human pancreas data for four heteroplasmy thresholds $h \in \{10\,\%, 30\,\%, 40\,\%, 95\,\%\}$.

S8.8 Volcano plots for all data sets

In the main manuscript, we show DEGs for the Enge data set (human pancreas). Here we show analogous plots for all data sets.

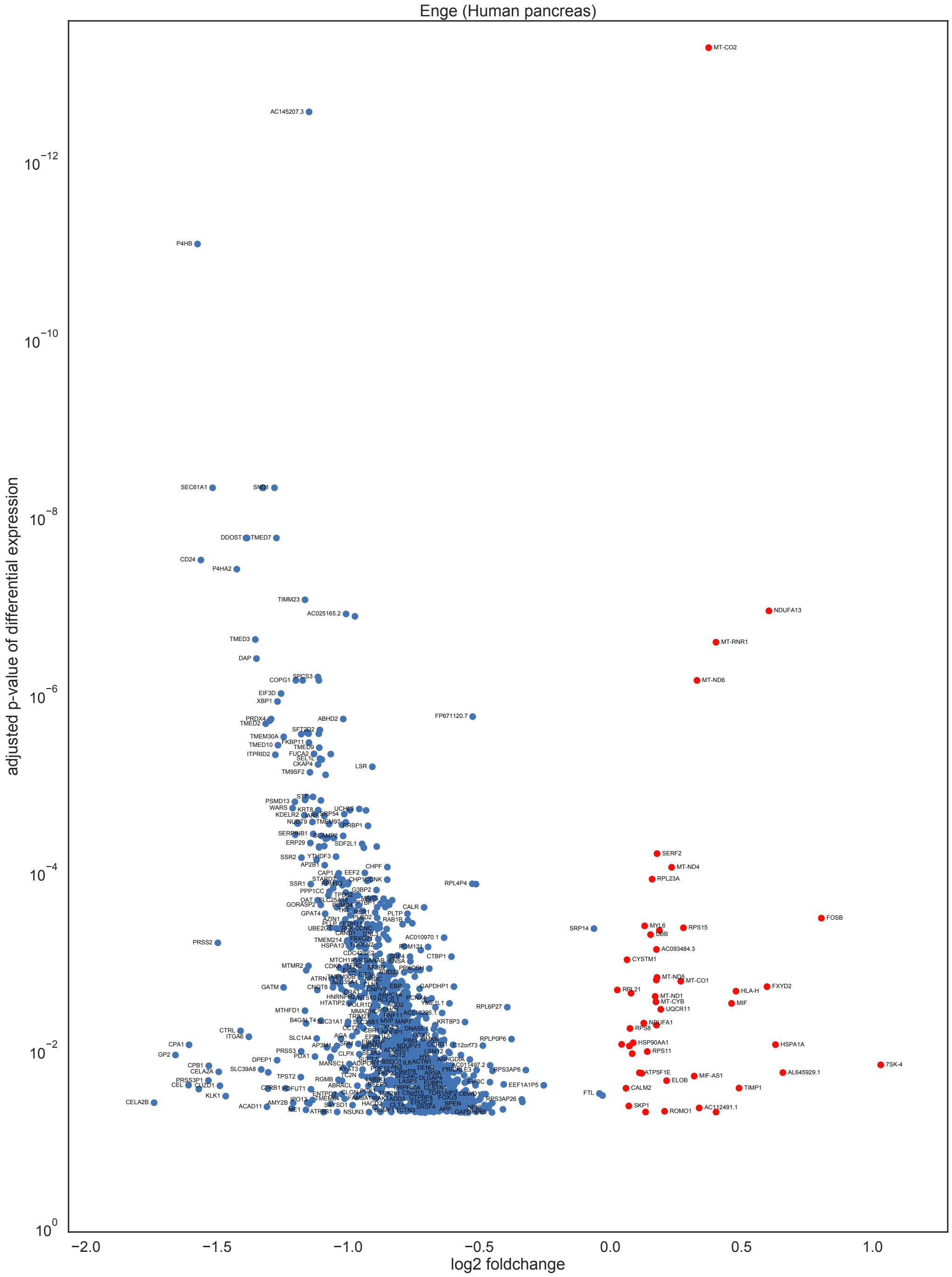

Supplementary Figure S32: **We show the 1342 differently expressed genes (DEGs) in the full-length human pancreas data (Enge (*14*)).** For each DEG, we show the log2-foldchange and the adjusted p-value of differential expression, as computed by a Wilcoxon rank sum test. We label selected genes.

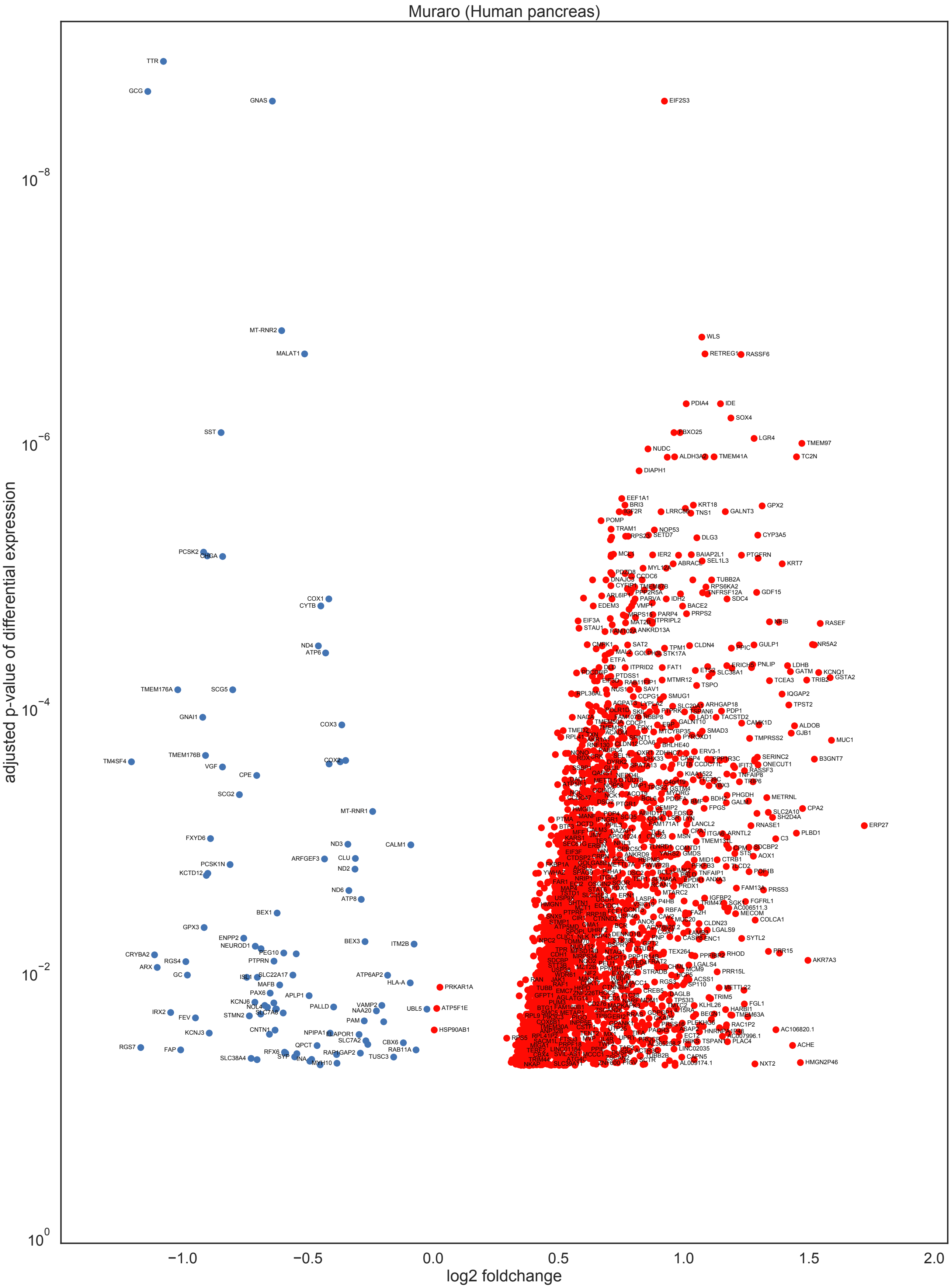

Supplementary Figure S33: **We show the 3319 differently expressed genes (DEGs) in the human pancreas data (Muraro (*20*)).** For each DEG, we show the log2-foldchange and the adjusted *p*-value of differential expression, as computed by a Wilcoxon rank sum test. We label selected genes.

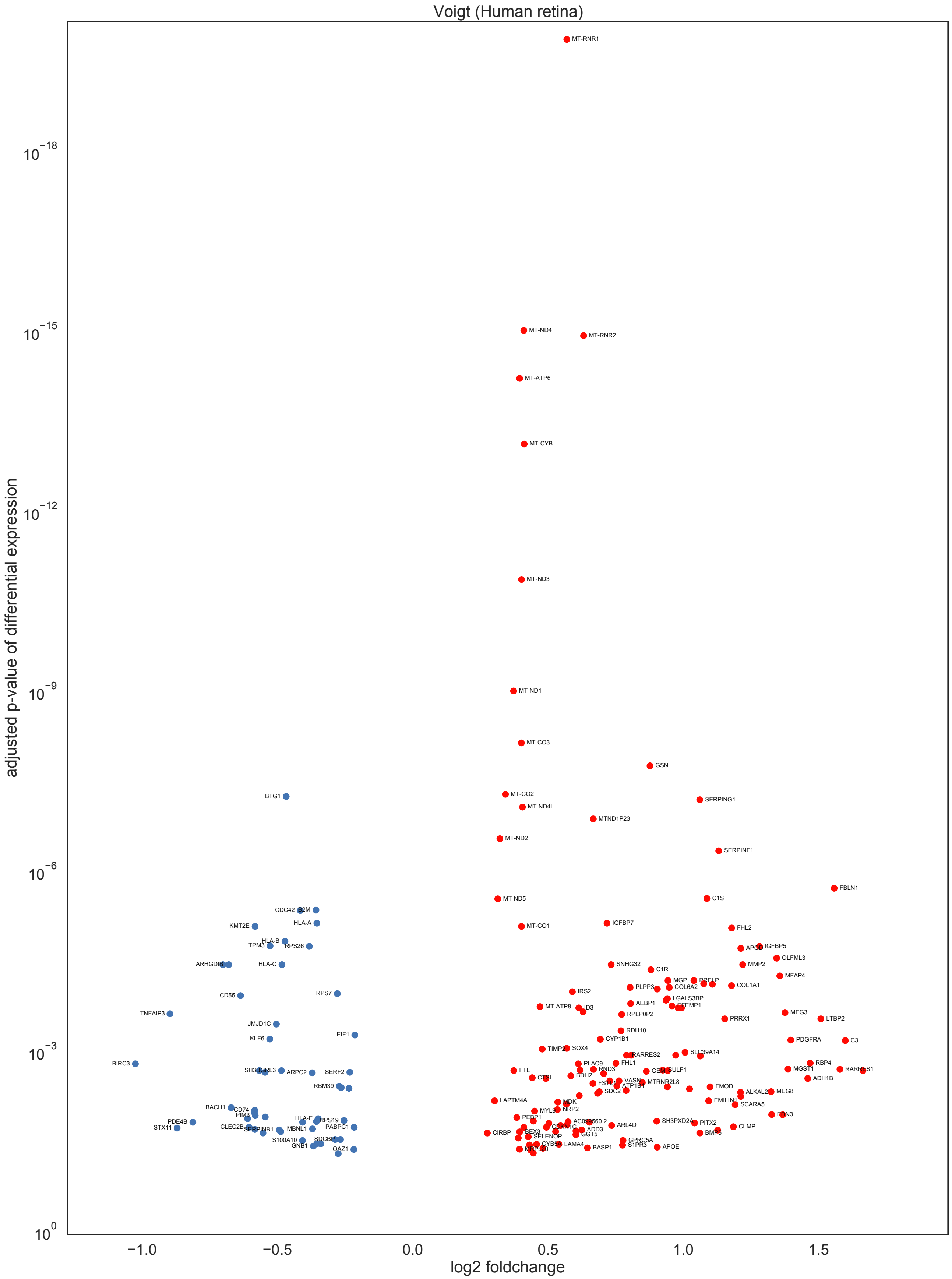

Supplementary Figure S34: **We show the 189 differently expressed genes (DEGs) in the human retina data (Voigt (*19*)).** For each DEG, we show the log2-foldchange and the adjusted *p*-value of differential expression, as computed by a Wilcoxon rank sum test. We label selected genes.

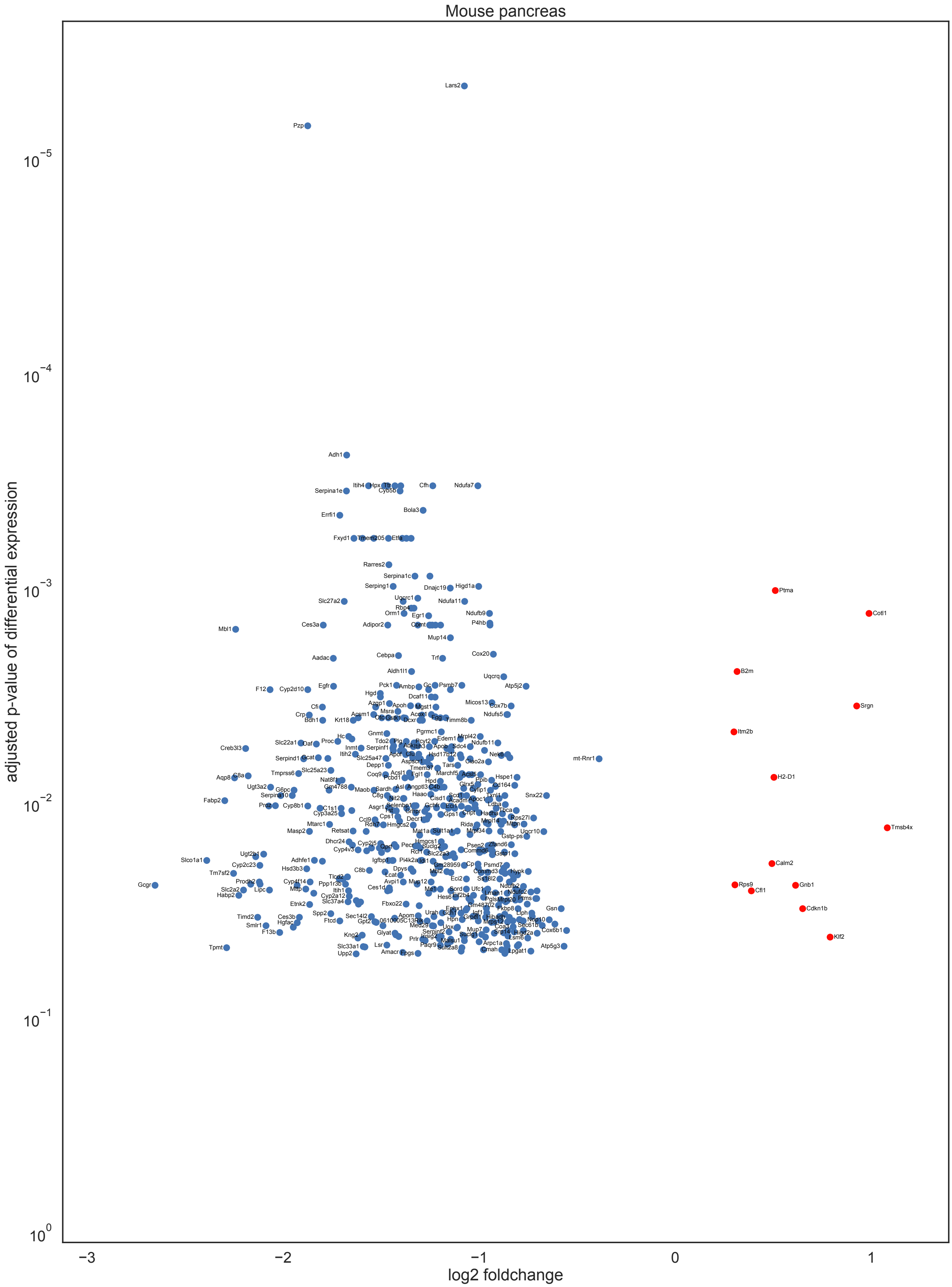

Supplementary Figure S35: **We show the 546 differently expressed genes (DEGs) in the mouse liver data (Tabula Muris (*21*)).** For each DEG, we show the log2-foldchange and the adjusted p-value of differential expression, as computed by a Wilcoxon rank sum test. We label selected genes.

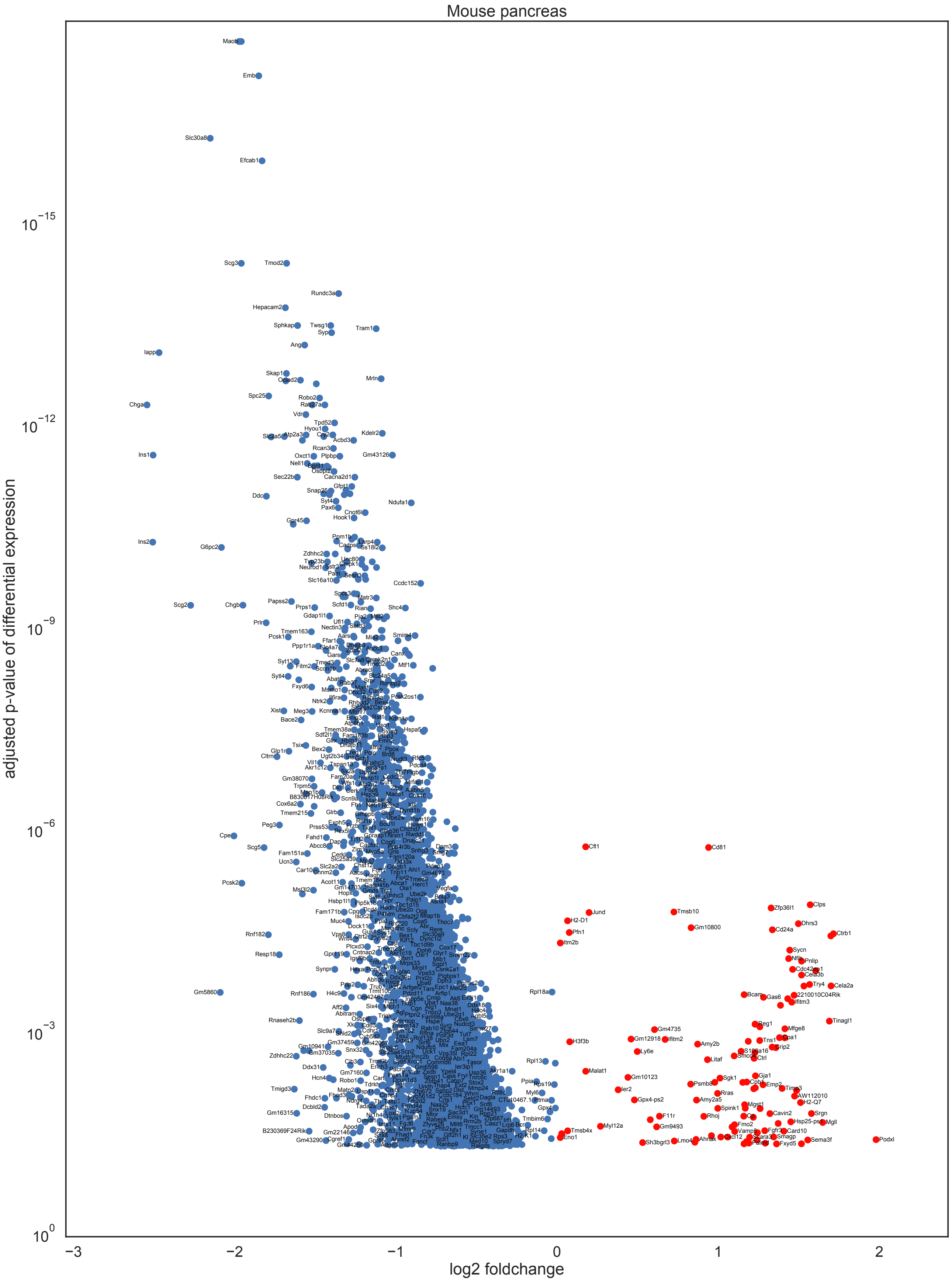

Supplementary Figure S36: **We show the 5430 differently expressed genes (DEGs) in the mouse pancreas data (Tabula Muris (*21*)).** For each DEG, we show the log2-foldchange and the adjusted *p*-value of differential expression, as computed by a Wilcoxon rank sum test. We label selected genes.

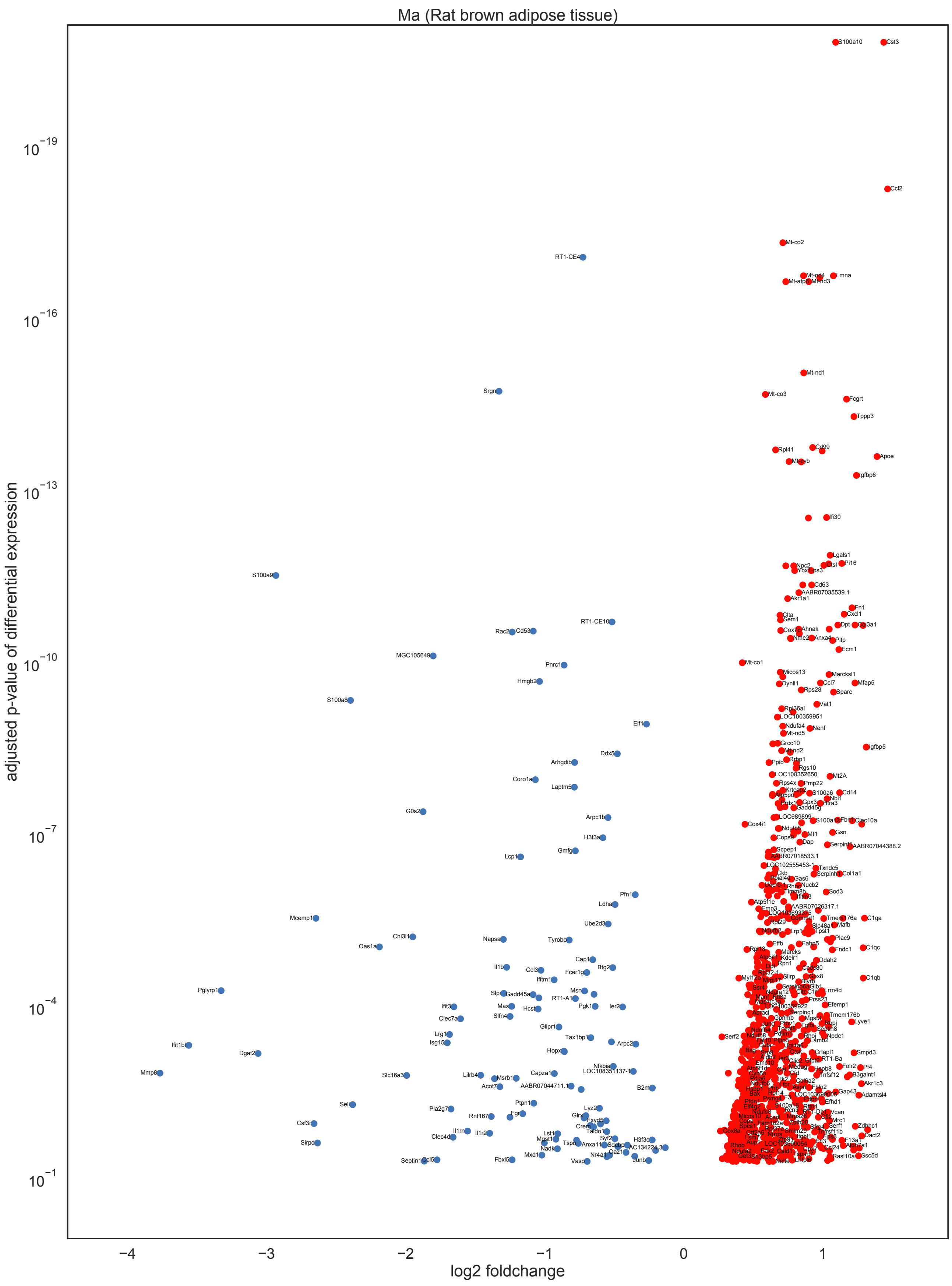

Supplementary Figure S37: **We show the 1691 differently expressed genes (DEGs) in the rat brown adipose tissue data (Ma *et al.* (*41*)).** For each DEG, we show the log2-foldchange and the adjusted *p*-value of differential expression, as computed by a Wilcoxon rank sum test. We label selected genes.

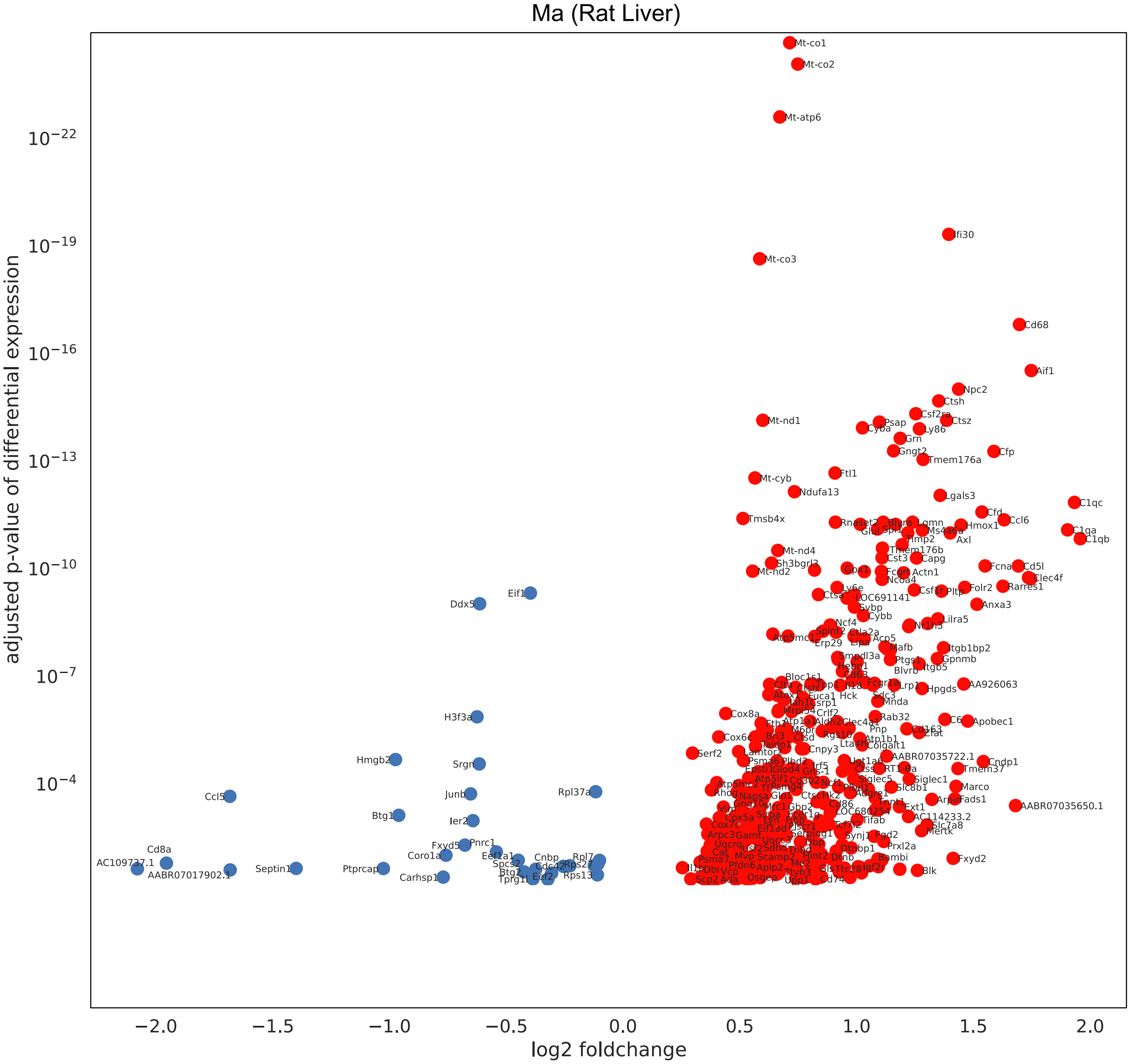

Supplementary Figure S38: **We show the 576 differently expressed genes (DEGs) in the rat liver data (Ma *et al.* (*41*)).** For each DEG, we show the log2-foldchange and the adjusted p-value of differential expression, as computed by a Wilcoxon rank sum test. We label selected genes.

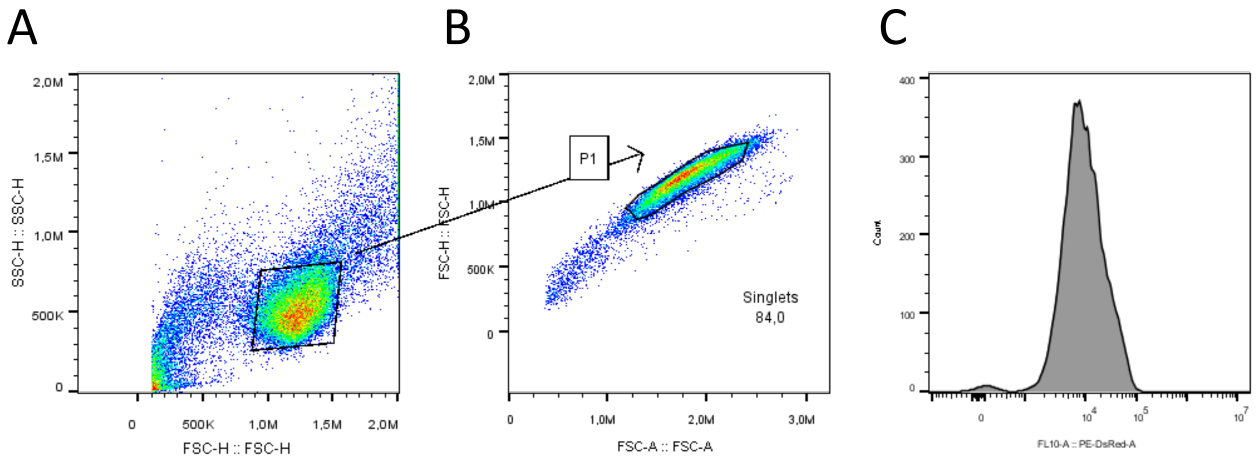

Supplementary Figure S39: **Flow cytometry scatter plots illustrate the gating strategy used to all the bioenergetic parameters.** Detection example of DCFDA that can be applied for the rest of FACS parameters (See Methods). a) Total population, at least 20000 events were recorded. b-c) Isolation of single cells and duplets exclusion. Selection of the population to be studied. The median of the fluorescence was used for comparisons.

S8.9 Processing pipeline

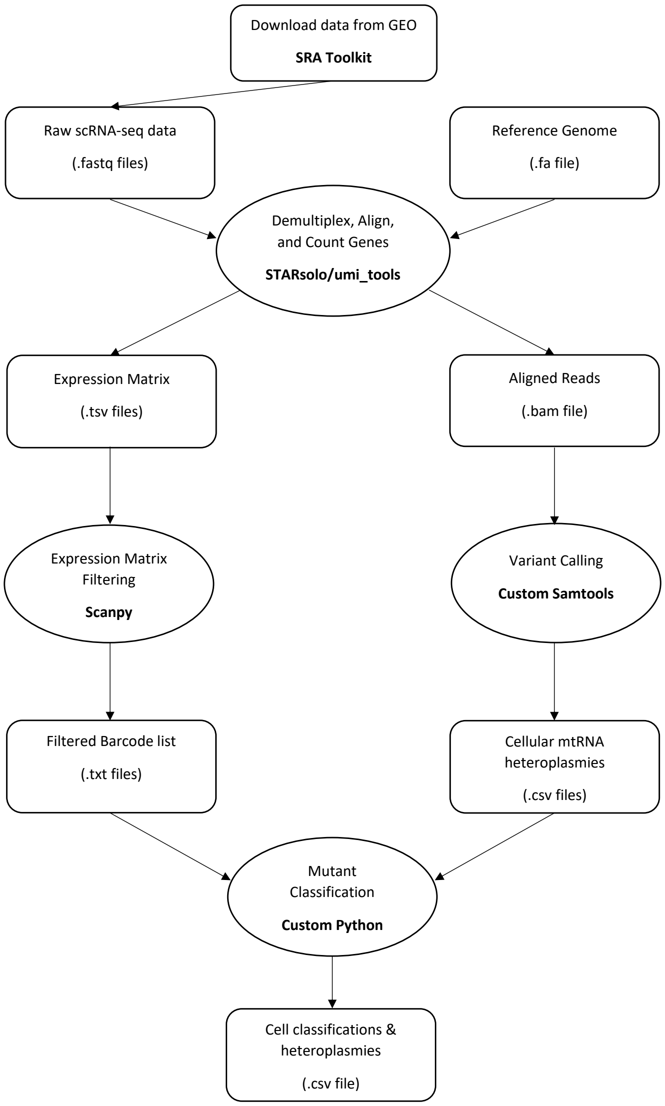

Supplementary Figure S40: **Directed acyclic graph representing the data processing pipeline.** After downloading the raw scRNA-seq data as .fastq files, we align them to a reference genome, which yields aligned reads in a .bam file and an expression matrix. Using custom Python tools, this gives us for each cell the heteroplasmy of mitochondrial mutations and also the gene-expression data. Then using custom Jupyter notebooks we perform quality control on the mitochondrial content of cells before assigning mutant classes to every heteroplasmy based on the number of cells from a donor it is found in.

S8.10 Differentially expressed genes for location-specific cryptic mtDNA mutations in human pancreas

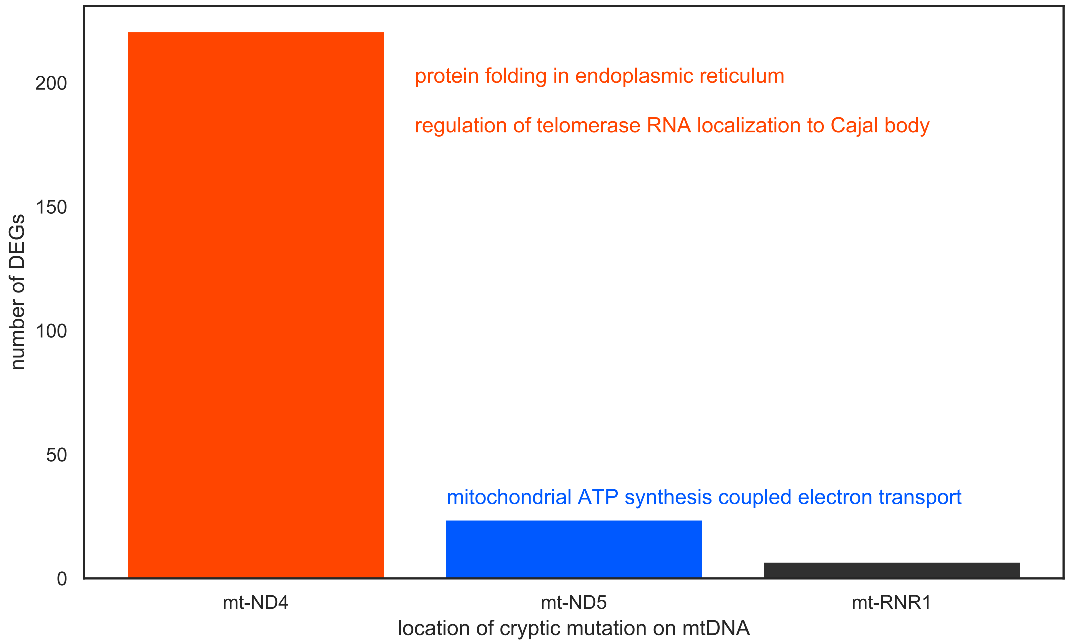

Supplementary Figure S41: **DEGs for cryptic mtDNA mutations are dominated by mutations at the mt-ND4, mt-ND5, and mt-RNR1 genes, each of which indicate distinct functional perturbations.** For the full-length human pancreas data (Enge), we compute DEGs at a $t = 10\,\%$ threshold for mutations that are located at each of the mitochondrial genes. For most mitochondrial genes, we do not find DEGs (not shown). For the mt-ND4, mt-ND5, and mt-RNR1 genes, however, we identify 220, 23, and 6 DEGs, respectively. A GO term-enrichment indicates that cryptic mt-ND4 mutations perturb 'protein folding in the ER' and 'regulation of telomerase RNA localization to Cajal body', which is consonant with the stress response that we discuss in the main manuscript. DEGs from cryptic mt-ND5 mutations in contrast are associated with various mitochondrial functions, particularly 'mitochondrial ATP synthesis coupled electron transport', indicating dysregulation of OXPHOS energy production. The six DEGs associated with cryptic mt-RNR1 mutations (TRAM1, CYSTM1, UBB, SSR2, AC093484.3, SF3B2) are not enriched for a GO term.

Supplementary References

1. R. Durrett, *Probability Models for DNA Sequence Evolution* (Springer-Verlag, ed. 2, 2008).
2. A. J. Berk, D. A. Clayton, *Journal of Molecular Biology* **86**, 801–824 (1974).
3. P. A. P. Moran, *Mathematical Proceedings of the Cambridge Philosophical Society* **54**, 60–71 (1958).
4. J. F. C. Kingman, *Journal of Applied Probability* **19**, 27–43 (1982).
5. P. Sjödin, I. Kaj, S. Krone, M. Lascoux, M. Nordborg, *Genetics* **169**, 1061–1070, ISSN: 0016-6731 (Feb. 2005).
6. I. Kaj, S. Krone, *Journal of Applied Probability* **40**, 33–48 (Mar. 2003).
7. A. Sano, A. Shimizu, M. Iizuka, *Theoretical Population Biology* **65**, 39–48, ISSN: 0040-5809 (2004).
8. J. Aryaman, C. Bowles, N. S. Jones, I. G. Johnston, *Genetics* **212**, 1429–1443 (2019).
9. R. A. Menzies, P. H. Gold, *Journal of Biological Chemistry* **246**, 2425–2429 (1971).
10. M. J. Fletcher, D. R. Sanadi, *Biochimica et Biophysica Acta* **51**, 356–360 (1961).
11. H. R. Lee, K. A. Johnson, *Journal of Biological Chemistry* **281**, 36236–36240 (2006).
12. C. Kukat *et al.*, *Proceedings of the National Academy of Sciences of the United States of America* **108**, 13534–13539 (2011).
13. A. Green, en-US-GB, (`http://spiral.imperial.ac.uk/handle/10044/1/114784`) (Sept. 2022).
14. M. Enge *et al.*, *Cell* **171**, 321–330 (2017).
15. R. Arrojo e Drigo *et al.*, *Cell Metabolism* **30**, 343–351.e3 (2019).
16. S. Smajić *et al.*, *Brain* **145**, 964–978, ISSN: 0006-8950 (2022).
17. A. Grubman *et al.*, *Nature Neuroscience* **22**, 2087–2097 (2019).
18. M. R. Corces *et al.*, *Nature Genetics* **52**, 1158–1168 (2020).
19. A. P. Voigt *et al.*, *Proceedings of the National Academy of Sciences of the United States of America* **116**, 24100–24107 (2019).
20. M. J. Muraro *et al.*, *Cell Systems* **3**, 385–394.e3, ISSN: 2405-4712 (2016).
21. The Tabula Muris Consortium, *Nature* **583**, 590–595 (2020).
22. J. Camunas-Soler *et al.*, *Cell Metabolism* **31**, 1017–1031.e4, ISSN: 1550-4131 (2020).
23. M. G. Pezet *et al.*, *Communications Biology* **4**, 1–12 (2021).
24. J. B. Stewart, P. F. Chinnery, *Nature Reviews Genetics* **16**, 530–542 (2015).
25. M. T. Lin, D. K. Simon, C. H. Ahn, L. M. Kim, M. F. Beal, *Human Molecular Genetics* **11**, 133–145 (2002).
26. N. Amodio *et al.*, *Journal of Hematology & Oncology* **11**, 1–19 (2018).
27. J. Yao *et al.*, *EMBO Molecular Medicine* **8**, 346–362 (2016).
28. P. J. Batista, H. Y. Chang, *Cell* **152**, 1298–1307 (2013).
29. J. Middeldorp, E. Hol, *Progress in Neurobiology* **93**, 421–443 (2011).
30. R. K. Leak, *Journal of Cell Communication and Signaling* **8**, 293–310 (2014).
31. L. S. Ludwig *et al.*, English, *Cell* **176**, 1325–1339.e22 (2019).
32. J. Xu *et al.*, *eLife* **8**, ed. by R. L. Levine, M. E. Bronner, R. L. Levine, e45105 (2019).
33. C. A. Lareau *et al.*, *Nature Biotechnology*, 1–11 (2020).
34. Q. R. Xing *et al.*, *Genome Research* **30**, 1027–1039 (2020).
35. *Bioinformatics* **29**, 15–21 (2013).
36. E. Pienaar, M. Theron, M. Nelson, H. J. Viljoen, *Computational Biology and Chemistry* **30**, 102–111 (2006).
37. T. Christofi, A. Zaravinos, *Journal of Translational Medicine* **17**, 319 (2019).
38. T. Smith, A. Heger, I. Sudbery, *Genome Research* **27**, 491–499 (2017).
39. R. Desai *et al.*, *Science Advances* **6**, Publisher: American Association for the Advancement of Science, eabc9955 (2020).
40. A. S. Marshall, N. S. Jones, *Biology* **10**, 503, ISSN: 2079-7737 (2021).
41. S. Ma *et al.*, *Cell* **180**, 984–1001 (2020).
42. M. S. B. Raredon *et al.*, *Science Advances* **5**, Publisher: American Association for the Advancement of Science, eaaw3851 (2019).
43. R. Rossignol *et al.*, *Biochemical Journal* **370**, 751–762 (2003).
44. S. P. Burr, M. Pezet, P. F. Chinnery, *Development, Growth & Differentiation* **60**, 21–32 (2018).
