# Supplementary material for: Cryptic mitochondrial DNA mutations coincide with mid-late life and are pathophysiologically informative in single cells across tissues and species
Source: Nat Commun. 2025 Mar 6;16:2250. doi: 10.1038/s41467-025-57286-8 (PMC11885543; doi:10.1038/s41467-025-57286-8)
Supplement: Supplementary file 2 — Reporting Summary [file 41467_2025_57286_MOESM2_ESM.pdf]

nature portfolio

Corresponding author(s): Nick S. Jones

Last updated by author(s): Dec 17, 2024

Reporting Summary

Nature Portfolio wishes to improve the reproducibility of the work that we publish. This form provides structure for consistency and transparency in reporting. For further information on Nature Portfolio policies, see our Editorial Policies and the Editorial Policy Checklist.

Statistics

For all statistical analyses, confirm that the following items are present in the figure legend, table legend, main text, or Methods section.

n/a	Confirmed	
☐	☒	The exact sample size (*n*) for each experimental group/condition, given as a discrete number and unit of measurement
☒	☐	A statement on whether measurements were taken from distinct samples or whether the same sample was measured repeatedly
☐	☒	The statistical test(s) used AND whether they are one- or two-sided<br>*Only common tests should be described solely by name; describe more complex techniques in the Methods section.*
☐	☒	A description of all covariates tested
☐	☒	A description of any assumptions or corrections, such as tests of normality and adjustment for multiple comparisons
☐	☒	A full description of the statistical parameters including central tendency (e.g. means) or other basic estimates (e.g. regression coefficient) AND variation (e.g. standard deviation) or associated estimates of uncertainty (e.g. confidence intervals)
☐	☒	For null hypothesis testing, the test statistic (e.g. *F*, *t*, *r*) with confidence intervals, effect sizes, degrees of freedom and *P* value noted<br>*Give P values as exact values whenever suitable.*
☐	☒	For Bayesian analysis, information on the choice of priors and Markov chain Monte Carlo settings
☐	☒	For hierarchical and complex designs, identification of the appropriate level for tests and full reporting of outcomes
☐	☒	Estimates of effect sizes (e.g. Cohen's *d*, Pearson's *r*), indicating how they were calculated

Our web collection on statistics for biologists contains articles on many of the points above.

Software and code

Policy information about availability of computer code

Data collection	No software used for data collection.
Data analysis	STAR aligner (version 2.7.5c)<br>CEL-seq2 (no version number)<br>UMI-tools version 1.0.1<br>Scanpy version 1.7.2<br>scPPIN (version 0.3)<br>Various standard python libraries (e.g., numpy version 1.20.3, pandas version 1.2.4)<br><br>Detailed custom code and scripts are available on GitHub https://github.com/SystemsAndSignalsGroup/Mito-Ageing.

For manuscripts utilizing custom algorithms or software that are central to the research but not yet described in published literature, software must be made available to editors and reviewers. We strongly encourage code deposition in a community repository (e.g. GitHub). See the Nature Portfolio guidelines for submitting code & software for further information.

Data

Policy information about availability of data

All manuscripts must include a data availability statement. This statement should provide the following information, where applicable:

- Accession codes, unique identifiers, or web links for publicly available datasets
- A description of any restrictions on data availability
- For clinical datasets or third party data, please ensure that the statement adheres to our policy

All analysed data is publicly available from the Gene Expression Omnibus (GEO) website or Amazon Web Services (AWS).

GEO accession codes:
GSE81547, GSE85241, GSE135922, GSE147672, GSE133747, GSE65360, GSE137869, GSE157783, GSE138852, GSE124742.

https://tabula-muris.ds.czbiohub.org/

Research involving human participants, their data, or biological material

Policy information about studies with human participants or human data. See also policy information about sex, gender (identity/presentation), and sexual orientation and race, ethnicity and racism.

Reporting on sex and gender	Metadata on sex is available from the original data sources
Reporting on race, ethnicity, or other socially relevant groupings	*Please specify the socially constructed or socially relevant categorization variable(s) used in your manuscript and explain why they were used. Please note that such variables should not be used as proxies for other socially constructed/relevant variables (for example, race or ethnicity should not be used as a proxy for socioeconomic status).*<br>*Provide clear definitions of the relevant terms used, how they were provided (by the participants/respondents, the researchers, or third parties), and the method(s) used to classify people into the different categories (e.g. self-report, census or administrative data, social media data, etc.)*<br>*Please provide details about how you controlled for confounding variables in your analyses.*
Population characteristics	Age, diabetic status, Alzheimers status, Parkinsons Status
Recruitment	N/A
Ethics oversight	N/A

Note that full information on the approval of the study protocol must also be provided in the manuscript.

Field-specific reporting

Please select the one below that is the best fit for your research. If you are not sure, read the appropriate sections before making your selection.

☒ Life sciences ☐ Behavioural & social sciences ☐ Ecological, evolutionary & environmental sciences

For a reference copy of the document with all sections, see nature.com/documents/nr-reporting-summary-flat.pdf

Life sciences study design

All studies must disclose on these points even when the disclosure is negative.

Sample size	No sample-size calculation was performed.
Data exclusions	We excluded single cells if they have a low number of mitochondrial reads aligned to them. Furthermore, we filter each expression matrix using three covariates: the total counts per cell, total genes per cell, and the percentage of reads aligned to the mitochondrial genome.
Replication	N/A
Randomization	N/A
Blinding	N/A

Reporting for specific materials, systems and methods

We require information from authors about some types of materials, experimental systems and methods used in many studies. Here, indicate whether each material, system or method listed is relevant to your study. If you are not sure if a list item applies to your research, read the appropriate section before selecting a response.

Materials & experimental systems

n/a	Involved in the study	
	☒	Antibodies
	☒	Eukaryotic cell lines
☒		Palaeontology and archaeology
☒		Animals and other organisms
☒		Clinical data
☒		Dual use research of concern
☒		Plants

Methods

n/a	Involved in the study	
☒		ChIP-seq
	☒	Flow cytometry
☒		MRI-based neuroimaging

Antibodies

Antibodies used

4EBP1, ab32130. ABCAM 1:500
EIF2A 9722. CELL SIGNALING 1:500
Phospho-EIF2A (Ser51) (D9G8), 3398. CELL SIGNALING 1:500
B-Actin a1978. Validation by manufacters (correct size) and has been referenced in 2883 publications. SIGMA 1:500

Validation

4EBP1, ab32130. Validation by manufacters (correct size) and has been referenced in 6 publications.
Phospho-4E-BP1 (s65) 9451. Validation by manufacters (The cells were starved for 24 hours in serum-free medium and underwent a 1 hour amino acid deprivation. Amino acids were replenished for 1 hour. Cells were then either untreated (-) or treated with 100 nM insulin (+) for 30 minutes) and has been referenced in 368 publications.
EIF2A 9722. Validation by manufacters (correct size) and has been referenced in 571publications.
Phospho-EIF2A (Ser51) (D9G8), 3398. Validation by manufacters (correct size and untreated or thapsigargin-treated (300 nM) and has been referenced in 571 publications.
B-Actin a1978. Validation by manufacters (correct size) and has been referenced in 2883 publications.
Vinculin. V4505. Validation by manufacters (correct size) and has been referenced in 283 publications.

Eukaryotic cell lines

Policy information about cell lines and Sex and Gender in Research

Cell line source(s)

Transmitochondrial cybrids previously built using rho0 cellosaurus (CVCL_U293) osteosarcoma and their mitochondrial sequence was previously sequenced (Gomez-Duran, 2012, Martínez, 2015)

Authentication

The original cell line ho0 cellosaurus (CVCL_U293) osteosarcoma used to built the transmitochondrial cybrids was autheticated (Chomyn 1994)

Mycoplasma contamination

Cell lines were routinely checked for mycoplasma

Commonly misidentified lines
(See ICLAC register)

No common misidentified cell lines were used in this study

Plants

Seed stocks

N/A

Novel plant genotypes

N/A

Authentication

N/A

Flow Cytometry

Plots

Confirm that:

☐ The axis labels state the marker and fluorochrome used (e.g. CD4-FITC).

☐ The axis scales are clearly visible. Include numbers along axes only for bottom left plot of group (a 'group' is an analysis of identical markers).

☐ All plots are contour plots with outliers or pseudocolor plots.

☒ A numerical value for number of cells or percentage (with statistics) is provided.

Methodology

Sample preparation	Samples were prepared accordingly to the described methods
Instrument	Beckman-Coulter,Cytoflex-S
Software	FlowJo Software
Cell population abundance	20000 events were recorded
Gating strategy	20000 events were recorded and doublet discrimination was carried using FCS Height and Area

☒ Tick this box to confirm that a figure exemplifying the gating strategy is provided in the Supplementary Information.
